# Lineage-specific regulatory changes in hypertrophic cardiomyopathy unraveled by single-nucleus RNA-seq and spatial transcriptomics

**DOI:** 10.1038/s41421-022-00490-3

**Published:** 2023-01-17

**Authors:** Xuanyu Liu, Kunlun Yin, Liang Chen, Wen Chen, Wenke Li, Taojun Zhang, Yang Sun, Meng Yuan, Hongyue Wang, Yunhu Song, Shuiyun Wang, Shengshou Hu, Zhou Zhou

**Affiliations:** 1 State Key Laboratory of Cardiovascular Disease, National Center for Cardiovascular Diseases, Fuwai Hospital, the Chinese Academy of Medical Sciences, Beijing, China; 2 Center of Laboratory Medicine, Beijing Key Laboratory for Molecular Diagnostics of Cardiovascular Diseases, Beijing, China; 3 Department of Cardiovascular Surgery, Fuwai Hospital, Beijing, China; 4 Department of Pathology, Fuwai Hospital, Beijing, China

**Keywords:** Transcriptomics, Mechanisms of disease

## Abstract

Hypertrophic cardiomyopathy (HCM) is the most common cardiac genetic disorder characterized by cardiomyocyte hypertrophy and cardiac fibrosis. Pathological cardiac remodeling in the myocardium of HCM patients may progress to heart failure. An in-depth elucidation of the lineage-specific changes in pathological cardiac remodeling of HCM is pivotal for the development of therapies to mitigate the progression. Here, we performed single-nucleus RNA-seq of the cardiac tissues from HCM patients or healthy donors and conducted spatial transcriptomic assays on tissue sections from patients. Unbiased clustering of 55,122 nuclei from HCM and healthy conditions revealed 9 cell lineages and 28 clusters. Lineage-specific changes in gene expression, subpopulation composition, and intercellular communication in HCM were discovered through comparative analyses. According to the results of pseudotime ordering, differential expression analysis, and differential regulatory network analysis, potential key genes during the transition towards a failing state of cardiomyocytes such as *FGF12*, *IL31RA*, and *CREB5* were identified. Transcriptomic dynamics underlying cardiac fibroblast activation were also uncovered, and potential key genes involved in cardiac fibrosis were obtained such as *AEBP1*, *RUNX1*, *MEOX1*, *LEF1*, and *NRXN3*. Using the spatial transcriptomic data, spatial activity patterns of the candidate genes, pathways, and subpopulations were confirmed on patient tissue sections. Moreover, we showed experimental evidence that in vitro knockdown of *AEBP1* could promote the activation of human cardiac fibroblasts, and overexpression of *AEBP1* could attenuate the TGFβ-induced activation. Our study provided a comprehensive analysis of the lineage-specific regulatory changes in HCM, which laid the foundation for targeted drug development in HCM.

## Introduction

Hypertrophic cardiomyopathy (HCM) is the most common cardiac genetic disorder with an estimated prevalence of 1 in 200^1^. HCM is the leading cause of sudden cardiac deaths (SCDs) in young people, accounting for 36% of SCDs in young athletes^2^. HCM is characterized by an increased left ventricular wall thickness in the absence of an associated cardiac or systemic disease^3^. Cardiomyocyte hypertrophy and disarray, and cardiac fibrosis are the key histopathological hallmarks of HCM^4^. Pathological cardiac remodeling occurs in the myocardium of HCM patients^5^, manifesting as cardiomyocyte dysfunction, escalated fibroblast activation (fibrosis), chronic inflammation, and cell death. If left untreated, pathological cardiac remodeling may lead to adverse events, including heart failure, arrhythmias, and death. In recent years, significant efforts have been made to develop therapeutic agents for HCM. MYK-461, for instance, inhibits cardiac myosin ATPase^6^. However, effective targeted drugs for HCM are still very limited. An in-depth elucidation of the cellular and molecular changes in pathological cardiac remodeling of HCM is pivotal for developing medical therapies to successfully prevent or mitigate the HCM progression.

Previous studies have employed bulk RNA-seq to explore the transcriptomic alterations in the cardiac tissue of HCM at the tissue level^7,8^. However, cell-type-specific changes could not be detected from bulk data. Single-cell or single-nucleus RNA-seq (snRNA-seq) can overcome this limitation and allows for unbiased dissection of the cellular changes at unprecedented resolution. snRNA-seq has been successfully applied to dissect the heterogeneity of the adult human heart under healthy conditions^9^. However, there is still a lack of research to explore the transcriptomic changes in cardiac interventricular septum (IVS) under the diseased condition of HCM at single-nucleus resolution. The recent advent of spatially resolved transcriptomics has greatly expanded our scope and power to study the pathogenesis mechanism of diseases by providing spatial information of gene expression that is lost in single-cell/nucleus data^10^. Integrated analysis of snRNA-seq and spatial transcriptomic data would profoundly improve our knowledge regarding the cellular and molecular changes of HCM.

In this study, we performed snRNA-seq of the cardiac tissues from HCM patients and healthy donors. We also conducted spatial transcriptomic assays on cardiac tissue sections from HCM patients. Comparative analyses were performed to explore lineage-specific changes in gene expression, subpopulation composition, and intercellular communication in HCM cardiac tissues. Potential key genes during the transition towards a failing state of cardiomyocytes or during the activation of fibroblasts were prioritized.

We showed experimental evidence that in vitro knockdown of *AEBP1* could promote the activation of human cardiac fibroblasts, and overexpression of *AEBP1* could attenuate the TGFβ-induced activation, which suggests that *AEBP1* may function as a transcription repressor in cardiac fibroblast activation. We hope that our study will expedite the therapeutic development for mitigating the progression to heart failure or attenuating cardiac fibrosis in HCM.

## Results

### Single-nucleus and spatial transcriptomic sequencing of the cardiac IVS tissues from HCM patients and healthy donors

**Fig. 1:**
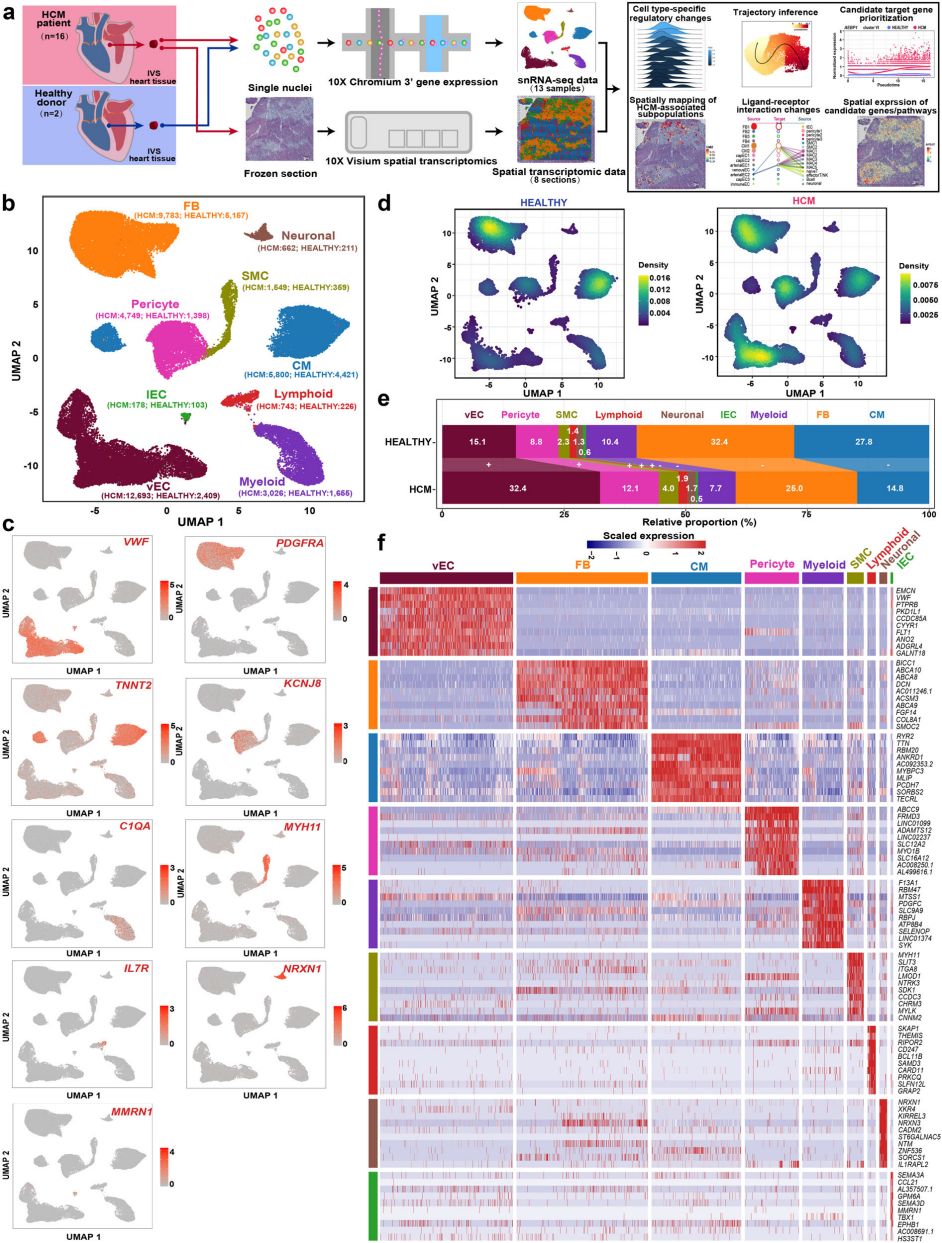
The changes in relative proportion for each cell type of human cardiac tissues in HCM. **a** Schematic representation of the overall experimental procedure. The cardiac IVS tissues of HCM patients who underwent surgical myectomy were collected for snRNA-seq (*n* = 10; 10 samples) and spatial transcriptomic assays (*n* = 6; 8 tissue sections). As a control, cardiac IVS tissues from healthy heart transplant donors (*n* = 2; 3 samples; the samples HEALTHY1A and HEALTHY1B were from the same donor) were subjected to snRNA-seq. **b** Unbiased clustering of 55,122 nuclei from all 13 samples identifies 9 major cell types. The nucleus count is indicated by the number in parenthesis. **c** UMAP plot showing the expression of the established marker genes for each cell type. **d** Comparison of the nucleus densities in the UMAP space between the two conditions reveals remarkable changes in the relative proportion of cell types in HCM. Nuclei were randomly sampled in equal numbers for each group (*n* = 15,939). **e** Relative proportion of each cell type in each condition. +: expansion; –: contraction. **f** Heatmap showing the molecular signature of each lineage. CM cardiomyocyte, FB fibroblast, lEC lymphatic endothelial cell, SMC smooth muscle cell, vEC vascular endothelial cell.

The cardiac IVS tissues of HCM patients who underwent surgical myectomy were collected for snRNA-seq (*n* = 10; 10 samples) and spatial transcriptomic assays (Fig. 1a; *n* = 6; 8 tissue sections). We also performed snRNA-seq for a control group (referred to as HEALTHY), which comprised cardiac IVS tissues from healthy donors of heart transplants (*n* = 2; 3 samples). The control group was ethnicity-, age-, and sex-matched with the HCM group (Chinese, male). The detailed demographic and clinical information of the enrolled subjects are outlined in Supplementary Table S1. All samples were sequenced individually. After quality control, a total of 55,122 nuclei (HCM: 39,183; HEALTHY: 15,939) were obtained (Supplementary Table S2). For the spatial transcriptomic data, 2927 to 4849 spots were detected (Supplementary Table S3). A web-based interface (http://snsthcm.fwgenetics.org/) was established for all datasets, which allowed for interactive examination of the expression of any gene or the activity of any pathway for both the snRNA-seq and spatial transcriptomic data.

### Expansion of vascular-related lineages and contraction of cardiomyocytes and fibroblasts in HCM

According to the expression of established markers for each lineage^9,11^ (Fig. 1b, c), 9 cell types were identified by joint clustering of the snRNA-seq data from both conditions: vascular endothelial cells (vECs, marked by *VWF*), fibroblasts (FBs, marked by *PDGFRA*), cardiomyocytes (CMs, marked by *TNNT2*), pericytes (marked by *KCNJ8*), myeloid cells (marked by *C1QA*), smooth muscle cells (SMCs, marked by *MYH11*), lymphoid cells (marked by *IL7R*), neuronal cells (marked by *NRXN1*), and lymphatic endothelial cells (lECs, marked by *MMRN1*). Comparing nucleus densities in the uniform manifold approximation and projection (UMAP) space between the two conditions revealed remarkable changes in the relative proportion of cell types in HCM, particularly for vECs, pericytes, and cardiomyocytes (Fig. 1d; Supplementary Figs. S1 and S2). Furthermore, we quantified the changes in cellular composition between the two conditions (Fig. 1e). Vascular-related lineages, including vECs, pericytes, and SMCs were greatly expanded. Cardiomyocytes and fibroblasts were greatly contracted, which potentially reflects the increased cell death in HCM. The distinct molecular signatures of each lineage are shown in Fig. 1f.

### Cardiomyocyte-specific regulatory changes in the pathological cardiac remodeling of HCM

**Fig. 2:**
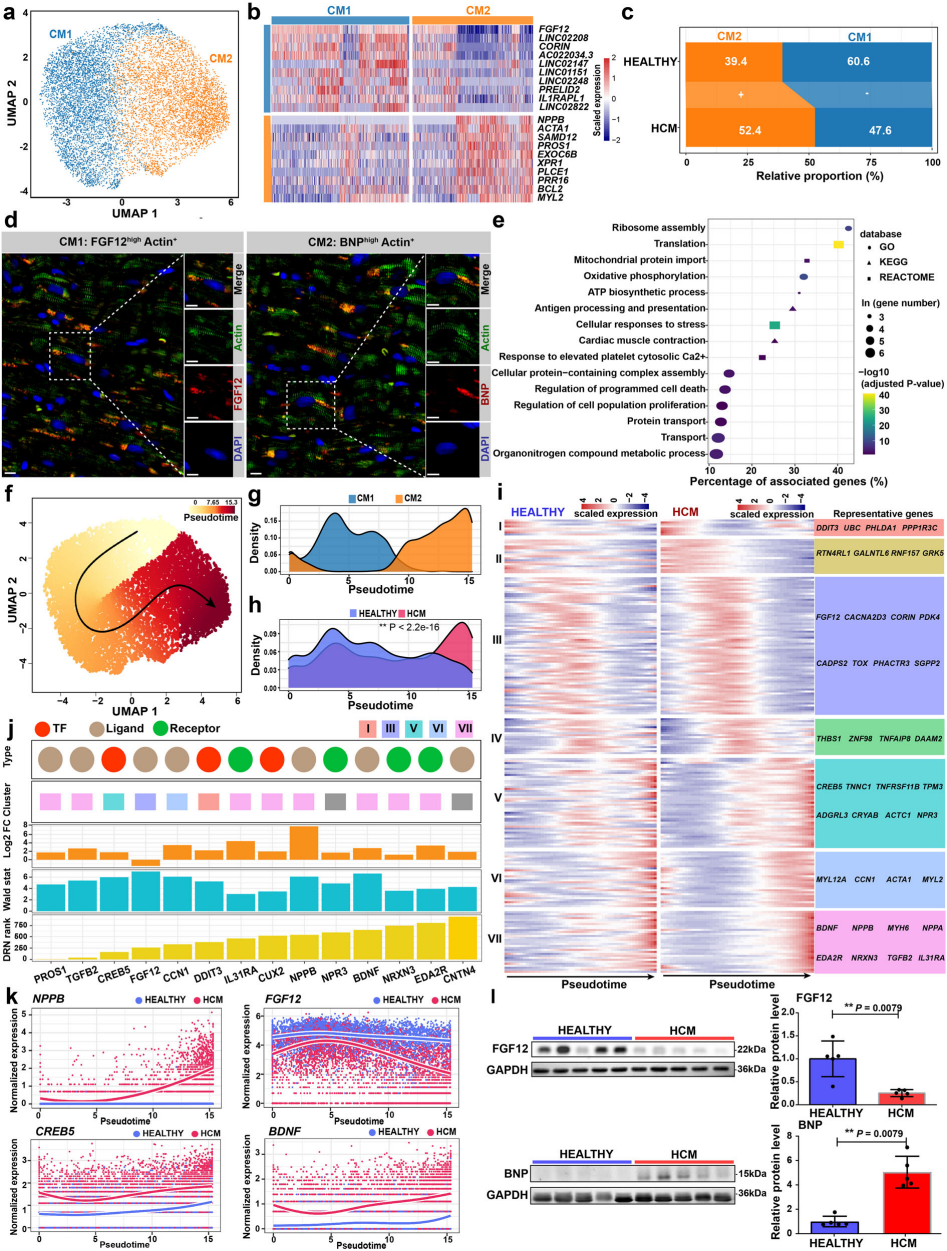
Cardiomyocyte-specific regulatory changes in the pathological remodeling of HCM. **a** UMAP plot showing the cardiomyocyte subpopulations. **b** Heatmap showing the molecular signatures of each subpopulation. **c** Relative proportion of each subpopulation in the cardiomyocytes from each condition. +: expansion; –: contraction. **d** Immunofluorescence staining confirmed the presence of the subpopulations in cardiac tissues from HCM patients. Cardiomyocytes are marked by Actin. BNP is encoded by the gene *NPPB*. Scale bar: 10 μm. **e** Representative terms enriched in the significantly upregulated genes in cardiomyocytes from HCM patients compared with those from healthy donors. Adjusted *P*-value < 0.05, hypergeometric test. **f** Cellular trajectory reconstructed for the transition towards failing cardiomyocytes using Slingshot. The arrow shows the direction of cellular state changes. **g** Density curves showing the distributions of the two subpopulations along the trajectory. **h** Density curves showing the distributions of the cardiomyocytes from two different conditions along the trajectory. ^**^P-value < 2.2e-16, Kolmogorov–Smirnov test. **i** Heatmaps showing the expression dynamics of the 216 genes with significantly different patterns along the trajectory between the two conditions. These genes were detected by differential expression pattern analysis using the “conditionTest” function of tradeSeq and were categorized into 7 gene clusters by hierarchical clustering. The significance threshold was set to be adjusted *P*-value < 0.05. **j** The potential key genes that were prioritized based on the results of three independent analyses including the difference in expression patterns, the fold change of expression levels, and the centrality change in GRNs. DRN rank: the gene ranking based on the centrality change in GRNs obtained by differential regulatory network analysis. Log2FC: log2 fold change of the expression levels in cardiomyocytes. Wald stat: the natural logarithm of the statistics of differential expression pattern analysis. Only genes encoding TFs, ligands, and receptors were considered. **k** Smoothed expression curves of representative candidate genes along the trajectory under both conditions. **l** Western blot assays confirmed that the protein levels of FGF12 and BNP were significantly changed in cardiac tissues from HCM patients (*n* = 5) compared with those from healthy donors (*n* = 5). ^**^P < 0.01, Wilcoxon ra*n*k-sum test.

Unbiased clustering grouped the cardiomyocytes into two subpopulations: CM1 and CM2 (Fig. 2a; Supplementary Table S4). CM2 expressed high levels of maladaptive markers for the reactivation of the fetal gene program such as *NPPB* (encoding natriuretic peptide B, a clinical biomarker for heart failure) and *ACTA1* (encoding skeletal α-actin)^12^, which thus denotes a subpopulation of cardiomyocytes towards a failing state (Fig. 2b). CM1 expressed high levels of *FGF12* and *CORIN*, which may represent cardiomyocytes in a relatively homeostatic or compensatory hypertrophy state. Consistent with this, CM2 was expanded in HCM, while CM1 was contracted (Fig. 2c). Immunofluorescence staining confirmed the presence of these two subpopulations on tissue sections from HCM patients (Fig. 2d) and healthy donors (Supplementary Fig. S3). Next, we applied DEsingle^13^ to detect the differentially expressed genes based on the snRNA-seq data in HCM versus HEALTHY for each lineage (Supplementary Table S5). For cardiomyocytes, 2021 genes and 486 genes were significantly upregulated and significantly downregulated, respectively (the absolute of log2 fold change >1, adjusted *P*-value < 0.05). In line with the pathological hypertrophy phenotype of HCM, the upregulated genes were enriched for terms associated with cell growth and protein synthesis (e.g., “Ribosome assembly” and “Translation”), energy metabolism (e.g., “Oxidative phosphorylation”), stress response (e.g., “Cellular responses to stress”), immune response (e.g., “Antigen processing and presentation”), cell death (e.g., “Regulation of programmed cell death”), metabolic reprogramming (e.g., “Organonitrogen compound metabolic process”), and contraction (e.g., “Cardiac muscle contraction”; Fig. 2e; Supplementary Table S6). The differentially regulated pathways were supported by gene set enrichment analysis (GSEA; Supplementary Table S7). Using the method implemented in bigScale2^14^, gene regulatory networks (GRNs) for each lineage were built separately for each condition (Supplementary Fig. S4). Moreover, we performed a comparative analysis of the GRNs between HCM and HEALTHY (differential regulatory networks analysis; DRN analysis) for each lineage, which allowed for gene ranking according to the changes in centrality, i.e., biological importance in the GRN (Supplementary Table S8). The representative genes with great changes in centrality were identified, such as *CRYAB* (Crystallin Alpha B), *EIF1* (Eukaryotic Translation Initiation Factor 1), *S100A1* (S100 Calcium Binding Protein A1), *PROS1* (Protein S), and *CREB5* (CAMP Responsive Element Binding Protein 5).

### Transcriptomic dynamics during the transition towards a failing state of cardiomyocytes in HCM

To decipher the transcriptomic dynamics during the transition towards a failing state of cardiomyocytes in HCM, we reconstructed the trajectory through the pseudo-temporal ordering of the nuclei of cardiomyocytes using Slingshot^15^ (Fig. 2f). The failing cardiomyocytes of CM2 were ordered at a relatively later stage along pseudotime trajectory (Fig. 2g). Significant differences existed between the pseudotime distributions of the two conditions (Fig. 2h; *P*-value < 2.2e-16, Kolmogorov–Smirnov test). By using tradeSeq^16^, the genes exhibiting significantly different expression patterns along the trajectory between the two conditions were identified and clustered into 7 gene clusters (Fig. 2i; Supplementary Table S9; adjusted *P*-value < 0.05). Notably, the maladaptive markers, *NPPB* and *NPPA*, were within the last gene cluster (VII).

Subsequently, we prioritized the potential key genes according to the results of three independent analyses, including the difference in expression patterns along the trajectory (adjusted *P*-value < 0.05), the fold change of expression levels between conditions (the absolute of log2 fold change > 1), and the centrality change in GRNs (DRN rank <1000). Only genes encoding Transcription factors (TFs), ligands, and receptors were considered. 14 candidate genes were prioritized (Fig. 2j). For most of the candidate genes, the differential expression was supported by pseudobulk RNA-seq analysis (adjusted *P*-value < 0.05, Supplementary Table S10). The roles of most genes in the transition of cardiomyocytes towards the failing state in HCM have not been recognized previously, such as *FGF12* (fibroblast growth factor 12), *CREB5*, and *BDNF* (brain-derived neurotrophic factor) (Fig. 2k). Regulon analysis by using SCENIC^17^ confirmed that the regulon activities of three prioritized TFs including *CREB5*, *CUX2*, and *DDIT3* were significantly higher in cardiomyocytes from HCM (adjusted *P*-value < 0.05, Wilcoxon rank-sum test; Supplementary Fig. S5 and Table S11). Notably, according to the results of bulk RNA-seq^7^ previously performed by our lab, some of the genes such as *FGF12*, *IL31RA*, and *PROS1* were significantly upregulated in the cardiac tissues of HCM (*q*-value < 0.05; Supplementary Fig. S6). Western blot assays confirmed that the protein levels of FGF12 and BNP (encoded by *NPPB*) were significantly changed in cardiac tissues from HCM patients (*n* = 5) versus healthy donors (*n* = 5; *P*-value < 0.05, Wilcoxon rank-sum test; Fig. 2l). In addition, we reanalyzed a published snRNA-seq dataset of cardiac tissues from dilated cardiomyopathy (DCM) patients and healthy donors^18^. We found that most of the key genes dysregulated in the cardiomyocytes of HCM such as *PROS1*, *FGF12*, *CREB5*, *TGFB2*, and *NPPB* were also dysregulated in DCM (Supplementary Fig. S7).

### Fibroblast-specific regulatory changes in the pathological cardiac remodeling of HCM

**Fig. 3:**
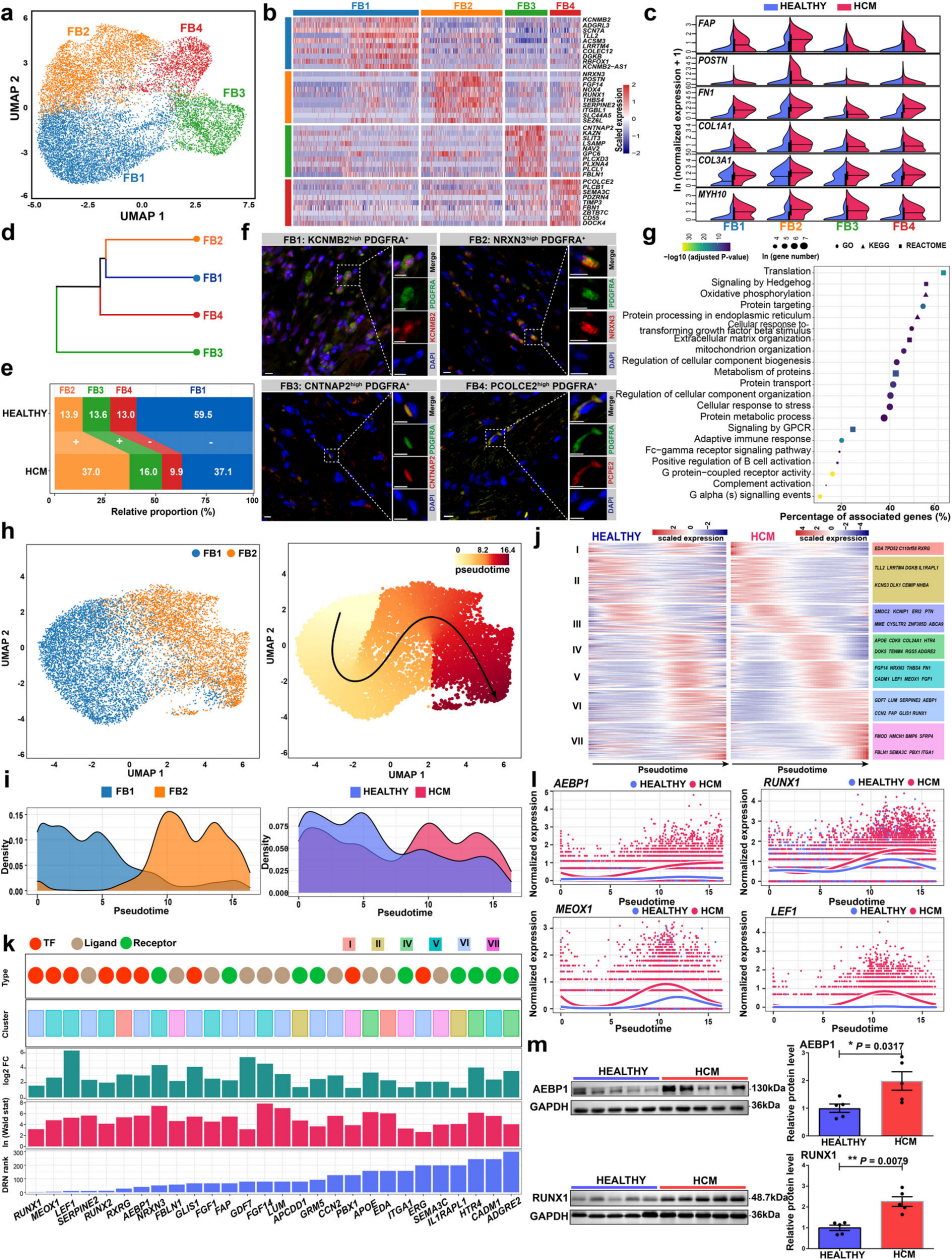
Fibroblast-specific regulatory changes in the pathological remodeling of HCM. **a** UMAP plot showing the fibroblast subpopulations. **b** Heatmap showing the molecular signature of each subpopulation. **c** Split violin plots showing the expression of the markers for activated fibroblasts. **d** Hierarchical clustering of the subpopulations. **e** Relative proportion of each subpopulation in the fibroblasts from each condition. +: expansion; –: contraction. **f** Immunofluorescence staining confirmed the presence of the four subpopulations in cardiac tissues from HCM patients. Fibroblasts are marked by PDGFRA. Scale bar: 10 μm. **g** Representative terms enriched in the upregulated genes in fibroblasts from HCM patients than those from healthy donors. Adjusted *P*-value < 0.05, hypergeometric test. **h** UMAP plot showing the subpopulations FB1 and FB2 (left panel), and the cellular trajectory reconstructed for fibroblast activation using Slingshot (right panel). The arrow shows the direction of cellular state changes. **i** Density curves showing the distributions of the two fibroblast subpopulations (left panel) and the fibroblasts from different conditions along the trajectory (right panel). ^**^P-value < 2.2e-16, Kolmogorov-Smirnov test. **j** Heatmaps showing the expression dynamics of the 432 genes with significantly different patterns along the trajectory between the two conditions. These genes were detected by differential expression pattern analysis using the “conditionTest” function of tradeSeq and were categorized into 7 gene clusters by hierarchical clustering. The significance threshold was set to an adjusted *P*-value < 0.05. **k** The potential key genes that were prioritized based on the results of three independent analyses including the difference in expression patterns, the fold change of expression levels, and the centrality change in GRNs. **l** Smoothed expression curves of representative candidate genes along the trajectory in both conditions. **m** Western blot assays confirmed that the protein levels of AEBP1 and RUNX1 were significantly changed in cardiac tissues from HCM patients (*n* = 5) compared with those from healthy donors (*n* = 5). ^*^P < 0.05, ^**^P < 0.01, Wilcoxon rank-sum test.

Four fibroblast subpopulations were identified through unbiased clustering: *KCNMB2* ^high^ FB1, *NRXN3* ^high^ FB2, *CNTNAP2* ^high^ FB3, and *PCOLCE2* ^high^ FB4 (Fig. 3a, b; Supplementary Table S4). Notably, FB2 expressed the highest levels of markers for activated fibroblasts (previously referred to as myofibroblasts^19^), including *FAP*, *POSTN*, *FN1*, *COL1A1*, *COL3A1*, and *MYH10*^20^, thus representing an activated state of fibroblasts (Fig. 3c). Hierarchical clustering revealed a close relationship between FB1 and FB2 (Fig. 3d). FB1 highly expressed markers for a basal transcriptomic program of cardiac fibroblasts that were previously described such as *SCN7A*, *ADGRL3*, and *TLL2*^9^, thus representing a basal state of fibroblasts (Fig. 3b). Consistent with this, FB2 was greatly expanded in HCM versus HEALTHY, while FB1 was greatly contracted (Fig. 3e). Immunofluorescence staining confirmed the presence of the four subpopulations on tissue sections from HCM patients (Fig. 3f) and healthy donors (Supplementary Fig. S8). Next, differentially expressed genes in fibroblasts were identified between the two conditions (Supplementary Table S5). In line with the fibrosis that occurred in HCM, fibrogenesis-associated terms such as “Extracellular matrix organization” and “Cellular response to transforming growth factor-beta stimulus” were found to be enriched in the upregulated genes (Fig. 3g; Supplementary Table S6). In addition, the upregulated genes were also enriched for terms related to protein translation and processing, energy metabolism, stress response, as well as immune response. Notably, Hedgehog and G protein-coupled receptor (GPCR) signaling were also enriched (Fig. 3g), which was consistent with their roles in fibrogenesis reported in other tissues and disease conditions^21,22^. In addition, *ADAM19* (ADAM metallopeptidase domain 19), *RUNX1* (RUNX family transcription factor 1), *CTIF* (cap-binding complex dependent translation initiation factor), *MEOX1* (mesenchyme homeobox 1), and *FGF7* (fibroblast growth factor 7) were identified as the top five genes with great changes in centrality via DRN analysis (Supplementary Fig. S9 and Table S8).

### Transcriptomic dynamics during the activation of cardiac fibroblasts in HCM

To decipher the transcriptomic dynamics during the activation of cardiac fibroblasts in HCM, we reconstructed the trajectory of fibroblast activation through the pseudo-temporal ordering of the FB1 and FB2 nuclei (Fig. 3h). Activated fibroblasts FB2 were ordered at the end of the trajectory (Fig. 3i). The fibroblasts from different conditions had significantly different pseudotime distributions (Fig. 3i; *P*-value < 2.2e-16, Kolmogorov–Smirnov test). Next, the genes exhibiting significantly different expression patterns along the trajectory between the two conditions were identified and clustered into 7 gene clusters (Fig. 3j; Supplementary Table S9; adjusted *P*-value < 0.05). The potential key genes were then prioritized according to the above-mentioned criteria. We prioritized 28 candidate genes, as shown in Fig. 3k. Notably, TF genes such as *AEBP1* (AE Binding Protein 1), *RUNX1*, *MEOX1*, and *LEF1* (lymphoid enhancer-binding factor 1) were significantly more upregulated along the trajectory of fibroblast activation in HCM versus HEALTHY (Fig. 3l). Regulon analysis confirmed that the regulon activities of four prioritized TFs including *LEF1*, *RUNX1*, *RUNX2*, and *PBX1* were significantly higher in fibroblasts from HCM (adjusted *P*-value < 0.05, Wilcoxon rank-sum test; Supplementary Fig. S10 and Table S11). Moreover, the bulk RNA-seq results^7^ showed that some of the genes such as *AEBP1*, *LEF1*, *NRXN3*, and *GLIS1* were significantly upregulated in the cardiac tissues of HCM (Supplementary Fig. S6). Western blot assays confirmed that the protein levels of AEBP1 and RUNX1, two representative candidate TFs, were significantly higher in cardiac tissues from HCM patients (*n* = 5) than healthy donors (*n* = 5; *P*-value < 0.05, Wilcoxon rank-sum test; Fig. 3m). In addition, we found that most of the key genes dysregulated in the fibroblasts of HCM such as *AEBP1*, *MEOX1*, *NRXN3*, *LEF1*, and *RUNX1* were also dysregulated in DCM (Supplementary Fig. S7).

### The immune and vascular lineage subpopulations and their changes in relative proportion in HCM

**Fig. 4:**
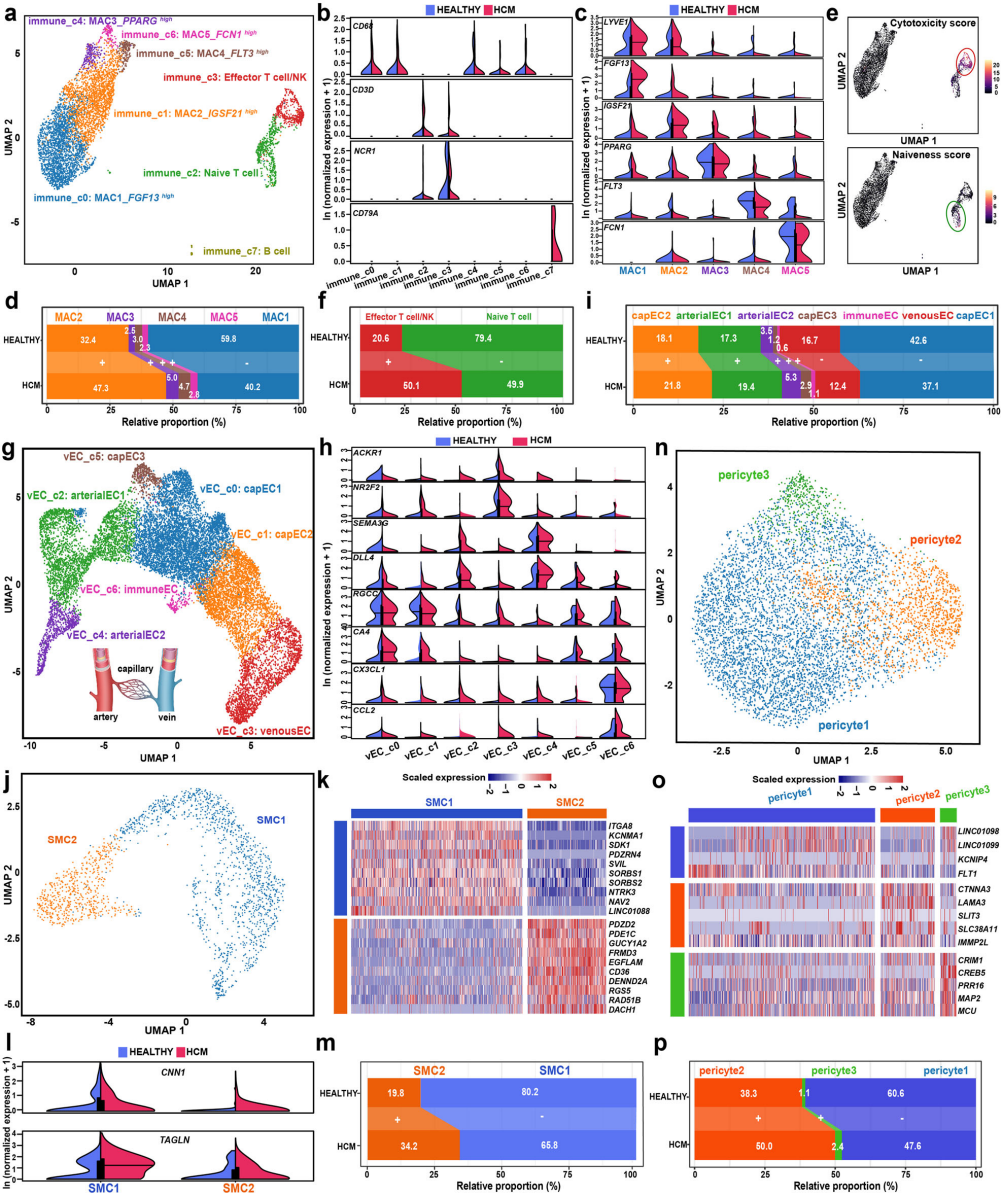
The immune and vascular lineage subpopulations and their changes in relative proportion in HCM. **a** UMAP plot showing the subpopulations of the immune lineage. **b** Expression of established markers for macrophages (*CD68*), T cells (*CD3D*), Natural killer cells (*NCR1*), and B cells (*CD79A*) in each immune subpopulation. **c** Expression of the marker for each of the five macrophage subpopulations. **d** Relative proportion of each subpopulation in macrophages of each condition. +: expansion; -: contraction. **e** UMAP plot showing the cytotoxicity and naiveness scores for each immune nucleus. The cytotoxicity and naiveness scores were calculated by summing the expression of previously reported signatures for T cell cytotoxicity (*PRF1*, *IFNG*, *GNLY*, *NKG7*, *GZMB*, *GZMA*, *GZMH*, *KLRK1*, *KLRB1*, *KLRD1*, *CTSW*, and *CST7*) and naiveness (*TCF7*, *SELL*, *LEF1*, and *CCR7*)^51^.**f** Relative proportion of each subpopulation of T/NK cells in each condition. **g** UMAP plot showing the subpopulations of the vECs. **h** Expression of the established markers for venous Ecs (*ACKR1* and *NR2F2*), arterial Ecs (*SEMA3G* and *DLL4*), capillary Ecs (*RGCC* and *CA4*), and immune Ecs (*CX3CL1* and *CCL2*). **i** Relative proportion of each subpopulation in the vECs from each condition. **j** UMAP plot showing the subpopulations of the SMCs. **k** Molecular signature for each SMC subpopulation. **l** Expression of contractile markers *CNN1* and *TAGLN* in each SMC subpopulation. **m** Relative proportion of each subpopulation in the SMCs from each condition. **n** UMAP plot showing the subpopulations of the pericytes. **o** Molecular signature for each pericyte subpopulation. **p** Relative proportion of each subpopulation in the pericytes from each condition. MAC: macrophage; SMC: smooth muscle cell; vEC: vascular endothelial cell.

Unbiased clustering identified 8 immune subpopulations (Fig. 4a). Immune_c0, c1, c4, c5, and c6 expressed high levels of *CD68* (Fig. 4b), thus representing five macrophage subpopulations (referred to as MAC1-5 hereafter). Figure 4c shows that *FGF13* ^high^ MAC1 and *IGSF21* ^high^ MAC2 expressed high levels of *LYVE1*, indicating that they were vessel-associated resident macrophages with M2-like phenotypes^23^. *FCN1*, which marks proinflammatory macrophages^24^, was found in high levels in MAC5. The cytokines that are well known to be pro-inflammatory, such as *IL1B* and *TNF*, exhibited the highest expression in the *FCN1* ^high^ macrophage subcluster (Supplementary Fig. S11). Comparative analysis of the relative proportion between conditions revealed an expansion of MAC2 and a contraction of MAC1 (Fig. 4d) in HCM, implying that MAC2 was more activated than MAC1. Immunofluorescence staining confirmed the presence of MAC2 under both conditions (IGSF21 ^high^ CD68^+^, Supplementary Fig. S12). Functional enrichment analysis supported macrophage immune activation in HCM (Supplementary Fig. S13a). Besides a small cluster of the nuclei of B cells (marked by *CD79A*), two other closely related lymphoid lineage subpopulations were identified: immune_c2 and immune_c3. Immune_c2 expressed high levels of the T cell marker *CD3D* (Fig. 4b) and exhibited high naiveness scores (Fig. 4e), indicating that it represented nuclei of naive T cells. Immune_c3 expressed high levels of the T cell marker *CD3D* and the natural killer (NK) cell marker *NCR1*, and exhibited high cytotoxicity scores (Fig. 4e), indicating that it represented a mixture of the nuclei of effector T/NK cells. As expected, we observed an expansion of the effector T/NK nuclei and a contraction of the naive T nuclei (Fig. 4f).

A total of 7 subpopulations within the vEC lineage that were aligned consecutively in the UMAP space were identified (Fig. 4g). Based on the established markers^9^, and from the left to the right of UMAP1, the subclusters were assigned to arterial ECs (marked by *SEMA3G* and *DLL4*; arterial EC2 and arterial EC1), capillary ECs (marked by *RGCC* and *CA4*; capEC3, capEC1, immune EC and capEC2) and venous ECs (marked by *ACKR1* and *NR2F2*; venousEC; Fig. 4h). Except for capEC1 and venousEC, most subpopulations were expanded (Fig. 4i). Two subpopulations of SMCs with distinct expression profiles were identified: SMC1 and SMC2 (Fig. 4j, k). SMC2 expressed lower levels of contractile markers such as *CNN1* and *TAGLN* than SMC1 (Fig. 4l) and was closely linked to pericytes in the UMAP space (Supplementary Fig. S14). These findings imply that SMC2 may represent modulated SMCs of the small vasculature in diseased conditions. Consistent with this, an expansion of SMC2 was observed (Fig. 4m). Immunofluorescence staining confirmed the presence of SMC2 under both conditions (*RGS5*^*high*^*MYH11*^+^, Supplementary Fig. S12). Three subpopulations of pericytes were identified: pericyte1, pericyte2, and pericyte3 (Fig. 4n, o), and pericyte2 was found to be significantly expanded in HCM (Fig. 4p). The representative pathways upregulated in each of the three types of vascular lineage are shown in Supplementary Fig. S13. Notably, energy metabolism and immune response-related pathways were upregulated in all three cell types, as they were in cardiomyocytes and fibroblasts.

### Changes in intercellular communication in HCM cardiac tissue inferred from the snRNA-seq data

**Fig. 5:**
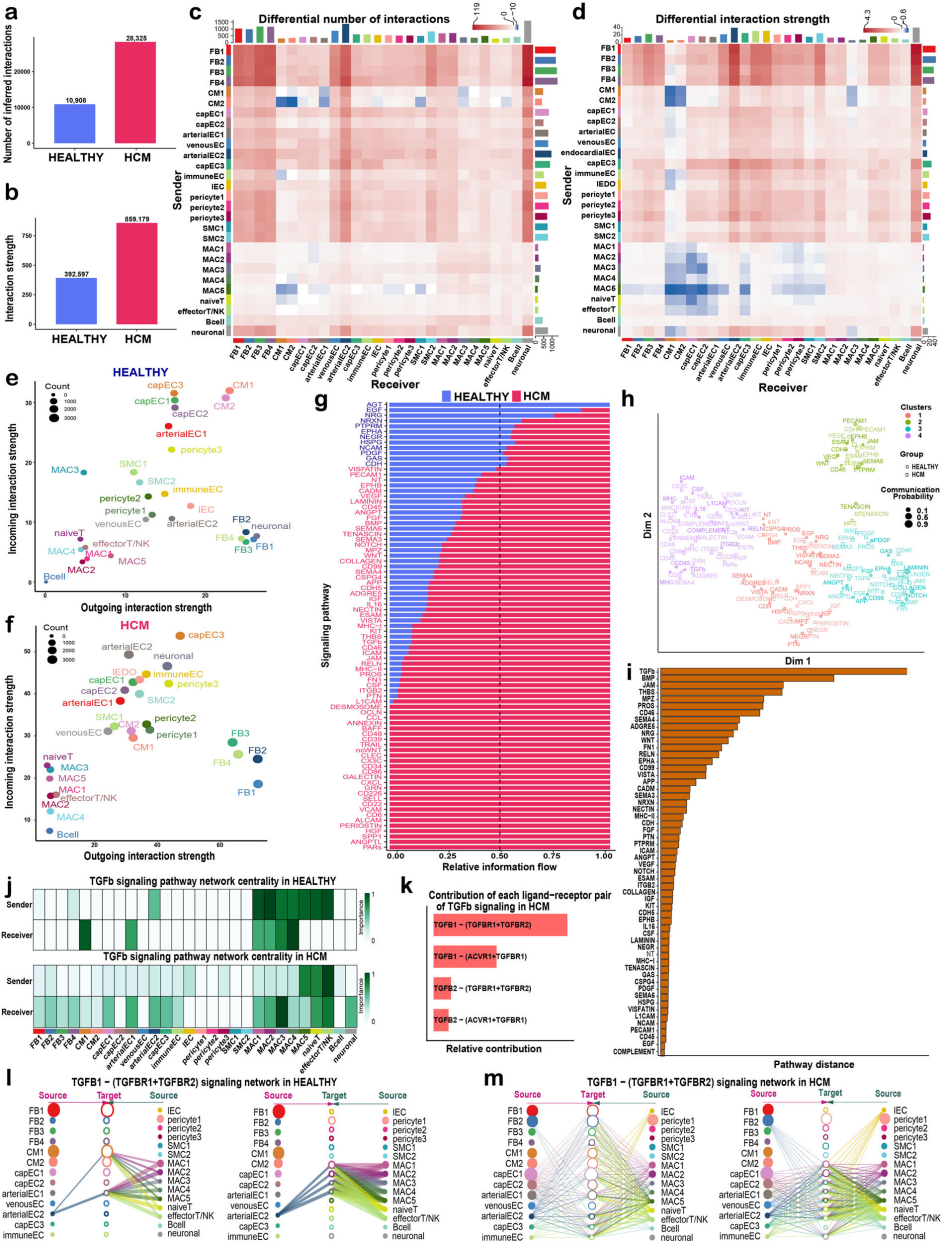
Intercellular communication changes in HCM cardiac tissues inferred from the snRNA-seq data. **a** Bar plot showing the total number of ligand-receptor interactions among the subpopulations of the cardiac tissues in both conditions. **b** Bar plot showing the total interaction strength among the subpopulations of the cardiac tissues in both conditions. The total interaction strength was calculated by summing the communication probability of all inferred interactions. **c** Heatmap showing the differential number of interactions among subpopulations in HCM versus HEALTHY. In the color bar, red represents an increase in the number of interactions and blue represents a decrease in the number of interactions. The top bar plot shows the sum of the changes in the number of incoming signals for each subpopulation. The right bar plot shows the sum of the changes in the number of outgoing signals for each subpopulation. **d** Heatmap showing the differential interaction strength among subpopulations in HCM versus HEALTHY. **e** Bubble plot showing the incoming and outgoing interaction strength for each subpopulation in HCM. The dot size represents the count of interactions. **f** Bubble plot showing the incoming and outgoing interaction strength for each subpopulation in HEALTHY. **g** Relative information flow for each signaling pathway in both conditions. The information flow is defined by the sum of the communication probability among all pairs of subpopulations. **h** Joint manifold learning of the HCM and HEALTHY communication networks and grouping the signaling pathways based on functional similarity. A high degree of functional similarity means that the major senders and receivers are similar. **i** The Euclidean distance of each pathway in the learn joint manifold. A larger distance means a larger difference in functional similarity (i.e., similarity in senders and receivers) between the two conditions. Only overlapping pathways between the two conditions are shown. **j** The major senders and receivers of the TGFβ signaling pathway inferred through network centrality analysis in HEALTHY (upper panel) and HCM (lower panel). **k** Relative contribution of each ligand-receptor pair to the overall signal of the TGFβ pathway in HCM. **l** Hierarchical plot showing the inferred communication network for TGFB1-(TGFBR1 + TGFBR2) signaling in HEALTHY. **m** Hierarchical plot showing the inferred communication network for TGFB1-(TGFBR1 + TGFBR2) signaling in HCM. In **l** and **m**, open and solid circles represent target and source, respectively. Edge width represents the interaction strength and circle size is proportional to the number of nuclei in each subpopulation. Edges are color-coded based on the signal source.

Intercellular communication in HCM has primarily been characterized in vitro using coculture methods in previous studies. We applied CellChat^25^ to infer ligand-receptor interactions among subpopulations in vivo for each condition based on the snRNA-seq data (Supplementary Table S12). The inferred total number (Fig. 5a) and strength of interactions (Fig. 5b) were significantly increased in HCM, reflecting an enhanced intercellular communication in diseased conditions as has been reported in other diseases^26^. The number (Fig. 5c) and strength (Fig. 5d) of interactions for both outgoing and incoming signals increased significantly in fibroblast subpopulations, confirming their central roles in the pathological remodeling of HCM. Notably, neuronal cells exhibited significantly enhanced incoming signals from other lineages, e.g., fibroblasts. Remarkably, cardiomyocytes, particularly the failing subpopulation CM2, demonstrated decreased communication between themselves (autocrine) and with some other lineages (paracrine), such as macrophages. Comparing the relative positions of cardiomyocytes in the 2D signal space between HEALTHY (Fig. 5e) and HCM (Fig. 5f) also suggested a substantial change in communication.

Next, we compared the relative information flow for each signaling pathway between two conditions (Fig. 5g), and identified pathways that were greatly enhanced in HCM (e.g., PTN, ITGB2, CSF, PROS, ICAM, CD46, TGFb, MHC-1, ESAM, and WNT) or specific to the HCM condition (e.g., PARs, ANGPTL, and SPP1). The signaling pathways were grouped based on functional similarity (i.e., similarity in senders and receivers) using joint manifold learning of the inferred communication networks (Fig. 5h). The changes in the functional similarity between the two conditions were reflected by the Euclidean distance of each pathway in the learn joint manifold. The TGFβ pathway had the largest distance, as shown in Fig. 5i. In line with this, network centrality analysis confirmed that the TGFβ pathway greatly changed in senders and receivers in HCM (Fig. 5j), where the top sender changed from MAC2 in HEALTHY to effector T/NK cells in HCM, and the top receiver changed from CM1 to MAC3. Then, we found that TGFB1-(TGFBR1 + TGFBR2) was the ligand-receptor pair that contributed the most to the TGFβ signaling network in the HCM cardiac tissues (Fig. 5k). TGFB1-(TGFBR1 + TGFBR2) signaling was enhanced in HCM, and the paracrine signal of TGFB1 received by fibroblasts, cardiomyocytes, and vECs was predominately secreted by effector T/NK cells, naive T cells, and proinflammatory macrophages MAC5 (Fig. 5l, m).

### Spatially resolved determination of the expression of candidate genes, the activity of HCM-related pathways, and subpopulations by spatial transcriptomics

**Fig. 6:**
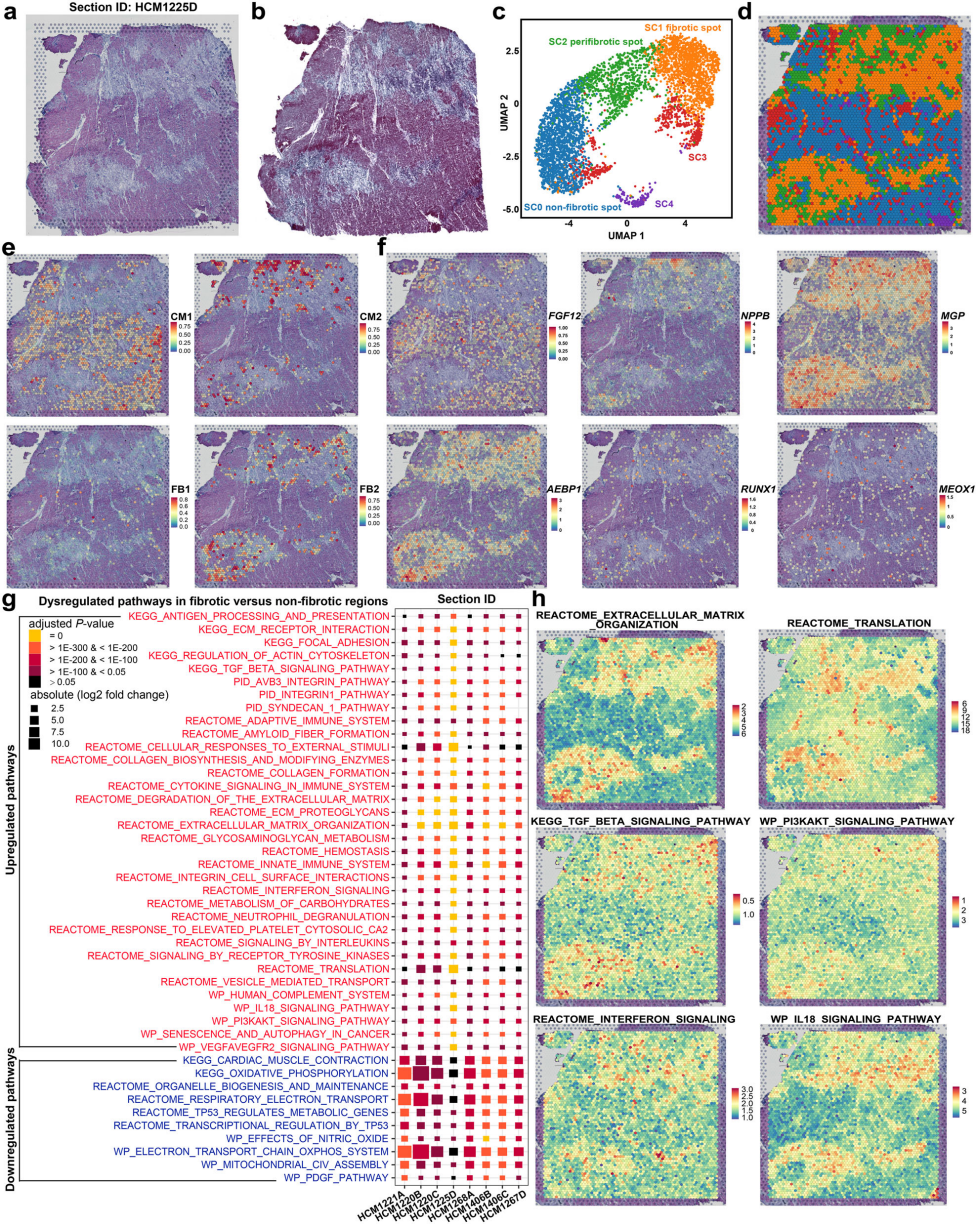
Spatially resolved determination of the expression of candidate genes, the activity of HCM-related pathways, and the distribution of subpopulations by spatial transcriptomics. **a** H&E staining image for the cardiac tissue section HCM1225D. **b** Masson’s trichrome staining image of a section adjacent to HCM1225D. **c** UMAP plot showing the spot clusters identified using unbiased clustering of the spots on HCM1225D. **d** Distribution of the spot clusters on the HCM1225D section. **e** Spatial location of the subpopulations FB1, FB2, CM1, and CM2 on the HCM1225D section predicted by integrating snRNA-seq data and spatial transcriptomic data. **f** Expression distribution of representative markers and candidate target genes on the section HCM1225D. **g** Dysregulated pathways in fibrotic versus non-fibrotic regions of the cardiac tissue sections of HCM. The dysregulated pathways were identified based on the pathway activity scores of each spot using the Wilcoxon rank-sum test. The significance threshold was set to a Bonferroni-adjusted *P*-value < 0.05 and an absolute of the log2 fold change > 0.25. HCM1220B and HCM1220C are sections from different samples of the same patient; HCM1406B and HCM1406C are neighboring sections from the same patient. **h** Activity of representative upregulated pathways in fibrotic regions on the HCM1225D section.

The eight tissue sections for spatial transcriptomic assays contained regions with replacement fibrosis and/or diffuse (interstitial or perivascular) fibrosis that commonly occur in HCM, as shown in Supplementary Figs. S15 and S16. For example, the HCM1225D section was characterized by extensive replacement fibrotic scars and interstitial fibrosis (also see Fig. 6a, b). Using unbiased clustering, spatial spots in fibrotic regions could be separated from those in non-fibrotic regions in all the sections (Supplementary Figs. S17–S23). For example, on the section HCM1225D, spot clusters SC0 and SC1 generally represented spots in fibrotic and non-fibrotic regions (Fig. 6c, d; Supplementary Fig. S24). We integrated the snRNA-seq data and the spatial transcriptomic data following the label transfer workflow of Seurat. CM1, the cardiomyocyte subpopulation in a homeostatic or compensatory hypertrophy state (marked by *FGF12*), was predicted to be located in non-fibrotic regions, whereas CM2, the cardiomyocyte subpopulation towards a failing state (marked by *NPPB*), was located close to the fibrotic regions (Fig. 6e, f; Supplementary Figs. S17–S23). The quiescent fibroblast subpopulation, FB1, was mostly in non-fibrotic regions, whereas the activated fibroblast subpopulation, FB2, was mostly found in fibrotic regions (Fig. 6e; Supplementary Figs. S17–S23). In fibrotic regions, the candidate genes *AEBP1*, *RUNX1*, and *MEOX1* were highly expressed (Fig. 6f; Supplementary Figs. S17–S23). Spatial cell-cell interaction (CCI) analysis by using stLearn (v0.3.1) revealed that intercellular communication hotspots were mainly localized in fibrotic or peri-fibrotic regions across all the sections (Supplementary Fig. S25). Moreover, the ligand-receptor pairs with enhanced communication activities in HCM that were predicted by CellChat based on the snRNA-seq data (Supplementary Table S13), such as GZMA-PARD3 and EFNA1-EPHA3, could be spatially verified through spatial CCI analyses (Supplementary Fig. S26). In addition, spatial pseudotime analysis through pseudo-temporal ordering of the spots using Monocle3 identified the transcriptomic dynamics during the change from non-fibrotic to fibrotic states of cardiac tissues in HCM (Supplementary Fig. S27 and Table S14). The expression patterns of representative markers or candidate genes along the pseudotime (e.g., *AEBP1*, *NPPB*, *FGF12*, and *RUNX1*) were consistent with those predicted based on the single-nucleus data. Therefore, the spatial transcriptomic data support the findings of our snRNA-seq analysis.

Next, for each section, the dysregulated genes and pathways in fibrotic versus non-fibrotic regions were identified (Supplementary Tables S15, S16). The upregulated pathways, as shown in Fig. 6g, were mainly involved in ECM remodeling (e.g., “REACTOME_EXTRACELLULAR_MATRIX_ORGANIZATION”), fibrosis-related signaling (e.g., “KEGG_TGF_BETA_SIGNALING_PATHWAY”), and immune response (e.g., “REACTOME_INTERFERON_SIGNALING”). The downregulated pathways were mainly involved in contraction (e.g., “KEGG_CARDIAC_MUSCLE_CONTRACTION”), energy metabolism (e.g., “KEGG_OXIDATIVE PHOSPHORYLATION”), and TP53-mediated stress response (e.g., “REACTOME_TRANSCRIPTIONAL_REGULATION_BY_TP53”). We also noticed that the identified dysregulated pathways were generally consistent among sections from different patients, between sections from different samples of the same patient (HCM1220B and HCM1220C), and between neighboring sections from the same patient (HCM1406B and HCM1406C; Fig. 6g). The spatial expression activity of representative pathways that exhibited upregulated activity in the fibrotic regions, such as the TGFβ signaling pathway, could be visualized on the tissue sections (Fig. 6h; Supplementary Fig. S28).

### In vitro knockdown of *AEBP1* promotes the activation of human cardiac fibroblasts

**Fig. 7:**
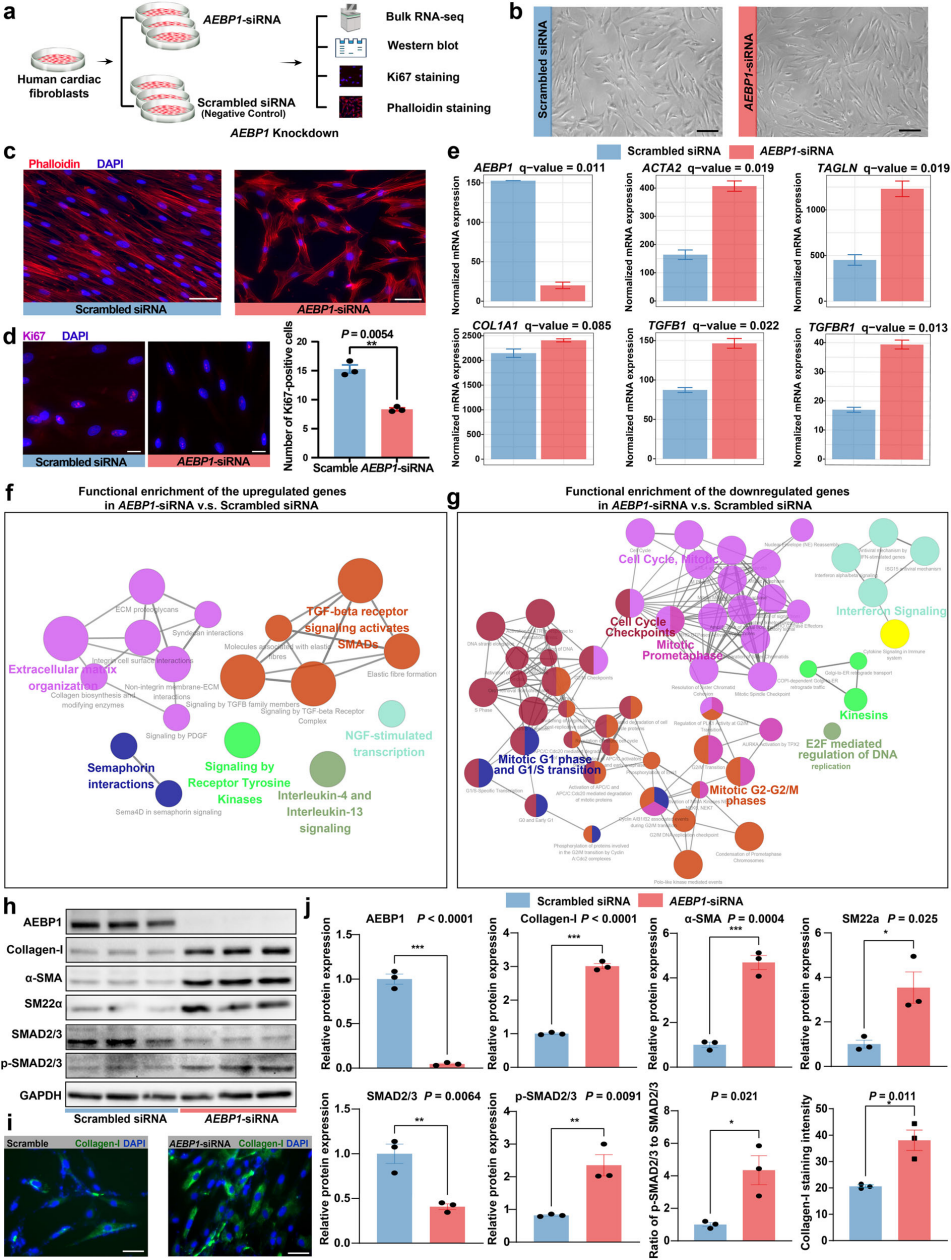
In vitro knockdown of *AEBP1* promotes the activation of human cardiac fibroblasts. **a** Schematic diagram showing the experimental procedure of *AEBP1* knockdown. Three independent experiments were performed for each group. Normal human ventricular cardiac fibroblasts (passage 5-7) were used. **b** Bright-field images showing the cardiac fibroblasts after 48 h of transfection. Scale bar: 200 μm. **c** Phalloidin staining of actin filaments showing the morphological changes of cardiac fibroblasts caused by *AEBP1* knockdown. Scale bar: 50 μm. **d** Ki67 staining showing that *AEBP1* knockdown reduced the proliferation of cardiac fibroblasts. In the bar plot, each value represents the mean number of Ki67-positive cells across five representative fields of view. Scale bar: 20 μm. ^**^P-value < 0.01, Student’s *t*-test. **e** mRNA expression of *AEBP1* and markers for fibroblast activation in both groups. The expression level is represented as tags per million reads. **f** Network plot showing the functional enrichment of the upregulated genes in *AEBP1*-siRNA versus scrambled siRNA. **g** Network plot showing the functional enrichment of the downregulated genes in *AEBP1*-siRNA versus scrambled siRNA. In **f** and **g**, each node denotes an over-represented Reactome pathway, and the node size reflects the statistical significance. Functionally associated terms were in the same color. The enrichment significant threshold was set to an adjusted *P*-value < 0.05. **h** Western blot assay showing the changes in protein expression of AEBP1 and representative fibroblast activation markers by the knockdown of *AEBP1*. **i** Immunofluorescence staining of cultured fibroblasts showing an increased protein expression level of Collagen-I by the knockdown of *AEBP1*. Scale bar: 50 μm. **j** Quantitative analysis of the western blot and staining results. For bar plots, the data are presented as means±standard error of the mean (SEM). For the staining results, each value represents the mean fluorescence intensity across five representative fields of view. ^*^P-value < 0.05, ^**^P-value < 0.01, ^***^P-value < 0.001. Student’s *t*-test.

To explore the role of *AEBP1*, one of the potential key TFs during cardiac fibroblast activation, we performed a siRNA-mediated knockdown of *AEBP1* in normal human cardiac fibroblasts (Fig. 7a, b). Phalloidin staining of actin filaments showed that the fibroblasts with *AEBP1* knockdown became triangular or polygonal, a morphology typical for activated fibroblasts, while the fibroblasts following scrambled siRNA transfection (negative control) remained a spindle-shaped morphology typical for unactivated fibroblasts (Fig. 7c). Ki67 (a nuclear antigen that marks cellular proliferation) staining showed that the proliferation of cardiac fibroblasts was significantly reduced by *AEBP1* knockdown (*P*-value < 0.05; Fig. 7d). Through bulk RNA-seq, we identified 484 significantly upregulated and 644 downregulated genes in *AEBP1*-siRNA versus scrambled siRNA (Supplementary Fig. S29 and Table S17). The knockdown of the mRNA expression of *AEBP1* was confirmed by bulk RNA-seq (Fig. 7e). Notably, *ACTA2* (encoding alpha-smooth muscle actin, αSMA) and *TAGLN* (encoding smooth muscle protein 22-alpha, SM22α), two markers for activated fibroblasts, were significantly upregulated in *AEBP1*-siRNA versus negative control (Fig. 7e). Genes encoding the ligand (*TGFB1*) and receptor (*TGFBR1*) of TGFβ signaling, the master pathway in fibroblast activation, were also significantly upregulated (Fig. 7e). Functional enrichment analysis revealed that the upregulated genes were mainly enriched for extracellular matrix organization and TGFβ signaling-related pathways (Fig. 7f), while the downregulated genes were mainly enriched for cell cycle-associated pathways (Fig. 7g). Furthermore, we examined the protein expression changes through western blot assays and immunofluorescence staining (Fig. 7h–j). The knockdown of *AEBP1* was confirmed at the protein level. Consistent with the mRNA expression changes, the protein levels of αSMA and SM22α were significantly increased. While the protein levels of SMAD2/3 were decreased by *AEBP1* knockdown, the phosphorylated SMAD2/3 (p-SMAD2/3, the key regulators in the canonical TGFβ pathway) and the ratio of p-SMAD2/3 to SMAD2/3 were significantly increased. Although the upregulation of mRNA expression of *COL1A1* did not reach statistical significance (Fig. 7e), the protein level of Collagen-I was significantly increased by *AEBP1* knockdown (Fig. 7g–i). Together, our results suggest that the knockdown of *AEBP1* can promote the activation of human cardiac fibroblasts, which implies that AEBP1 may function as a transcription repressor in cardiac fibroblast activation.

### In vitro overexpression of *AEBP1* attenuates TGFβ-induced activation of human cardiac fibroblasts

**Fig. 8:**
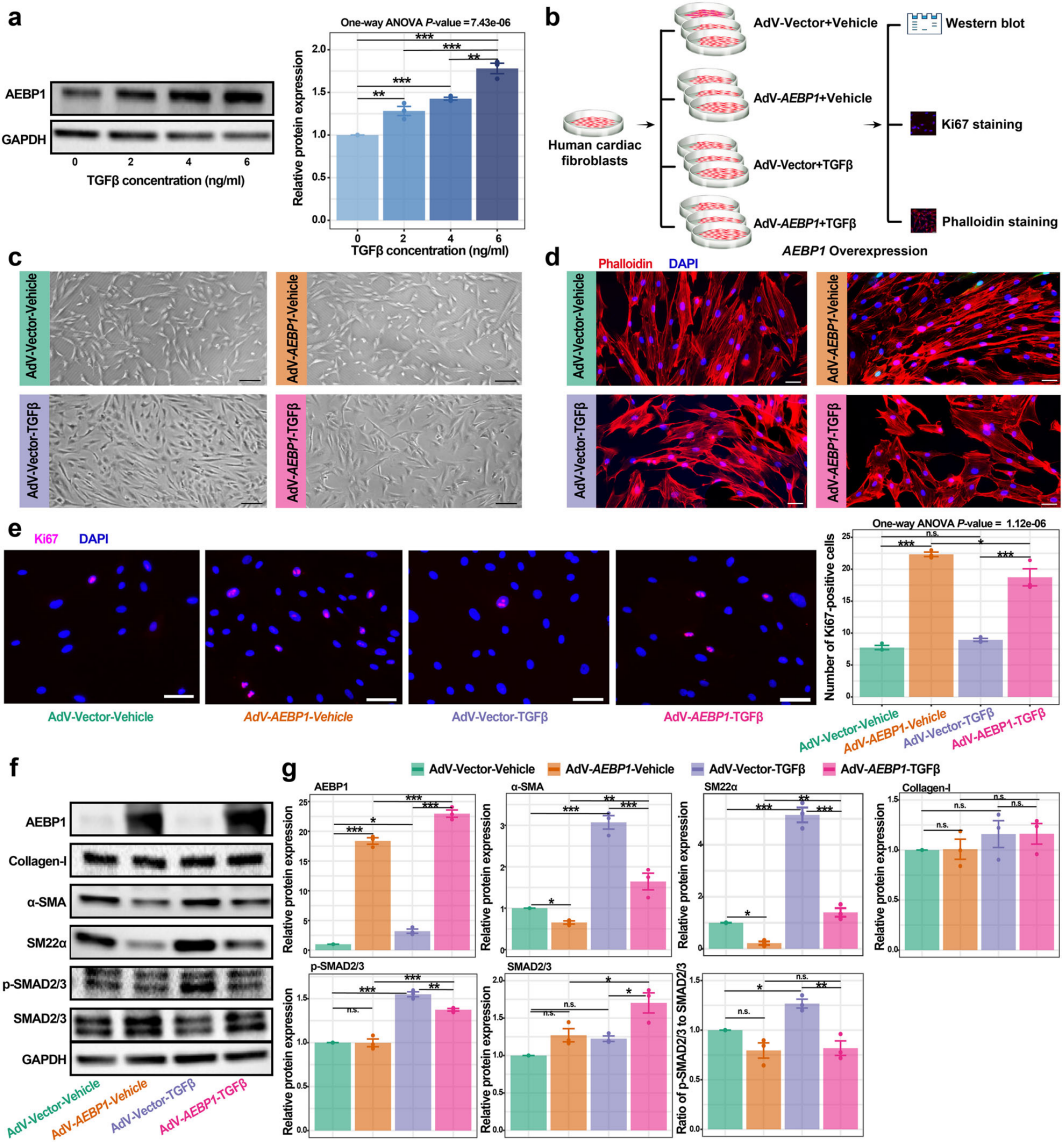
In vitro overexpression of *AEBP1* attenuates the activation of human cardiac fibroblasts induced by TGFβ. **a** The protein level of AEBP1 in cultured cardiac fibroblasts was significantly increased by TGFβ treatment. We chose 6 ng/mL TGFβ for the following experiments. **b** Schematic diagram showing the experimental design and procedure of *AEBP1* overexpression. AdV-Vector-Vehicle: fibroblasts transfected with adenovirus harboring empty vector and no TGFβ treatment was performed; AdV-*AEBP1*-Vehicle: fibroblasts transfected with adenovirus harboring *AEBP1* cDNA and no TGFβ treatment was performed; AdV-Vector*-*TGFβ: fibroblasts transfected with adenovirus harboring empty vector and followed by TGFβ treatment; AdV-*AEBP1-*TGFβ: fibroblasts transfected with adenovirus harboring *AEBP1* cDNA and followed by TGFβ treatment. Three independent experiments were performed for each group. Normal human ventricular cardiac fibroblasts (passages 5-6) were used. **c** Bright-field images of the cardiac fibroblasts in each group. Scale bar: 200 μm. **d** Phalloidin staining of actin filaments showing the cellular morphology of each group. Scale bar: 50 μm. **e** Ki67 staining showing that *AEBP1* overexpression enhanced the proliferation of cardiac fibroblasts. In the bar plot, each value represents the mean number of Ki67-positive cells across five representative fields of view. Scale bar: 50 μm. **f** Western blot assay showing the changes in protein expression of AEBP1 and representative fibroblast activation markers in each group. **g** Quantitative analysis of the western blot results. For bar plots, the data are presented as means ± SEM.^*^P < 0.05, ^**^P < 0.01, ^***^P < 0.001, n.s.: not significant, one-way ANOVA followed by Turkey post hoc tests.

The protein level of AEBP1 in cultured cardiac fibroblasts was significantly increased by TGFβ treatment (Fig. 8a), which is consistent with the upregulation of *AEBP1* in cardiac fibroblasts of HCM in vivo observed in our snRNA-seq dataset (Fig. 3l). Therefore, we applied TGFβ-induced activation of fibroblasts in vitro to further confirm the role of AEBP1 as a repressor in cardiac fibroblast activation. Adenoviral-mediated overexpression of *AEBP1* in cardiac fibroblasts was performed and followed by TGFβ treatment (Fig. 8b, c). Phalloidin staining of actin filaments showed that the fibroblasts with TGFβ treatment exhibited a typical morphology for activation (Fig. 8d). Ki67 staining showed that *AEBP1* overexpression greatly enhanced the proliferation of cardiac fibroblasts with or without TGFβ treatment (Fig. 8e), suggesting its pro-proliferation role. Western blot confirmed the overexpression of AEBP1 (Fig. 8f, g). Compared with the negative controls (adenovirus harboring empty vector), although no significant change was observed for Collagen-I, the protein levels of αSMA, SM22α, and pSMAD2/3 were significantly decreased by *AEBP1* overexpression, particularly with TGFβ treatment. The ratio of p-SMAD2/3 to SMAD2/3 was also significantly decreased by *AEBP1* overexpression with TGFβ treatment. Together, our results suggest that the overexpression of *AEBP1* can attenuate TGFβ-induced activation of human cardiac fibroblasts.

## Discussion

Understanding lineage-specific regulatory changes under diseased conditions is of fundamental importance for successful drug development. The present study provided a comprehensive analysis of lineage-specific changes in expression profile, subpopulation composition, and intercellular communication in the cardiac tissues of human HCM patients using snRNA-seq and spatial transcriptomic assays.

While cardiac remodeling is orchestrated by multiple lineages, cardiomyocytes act as the most important determinant of cardiac state. As such, pharmacological interventions that directly target cardiomyocytes may be the most promising strategy for alleviating pathological hypertrophy or mitigating the progression to heart failure in HCM. The single-nucleus resolution data allowed us to examine the cardiomyocyte-specific regulatory changes of HCM in vivo. The functional enrichment analysis (Fig. 2e) accurately reflected the features known for pathological cardiac hypertrophy^12^, such as increased protein translation, energy metabolism, stress response, immune response, cell death, and contraction. Some of the genes that were greatly changed in DRN centrality in HCM have been implicated in cardiac hypertrophy or heart failure. For example, *CRYAB* has been shown to suppress pressure overload-induced cardiac hypertrophy in mice^27^. *S100A1* has been suggested as a therapeutic target for heart failure^28^. However, the involvement of most genes in the pathogenesis of HCM, such as *FGF12* and *CREB5*, remains unknown. Cardiomyocytes were clustered into two subpopulations: *FGF12* ^high^ CM1 and *NPPB* ^high^ CM2 (Fig. 2a), which represented a homeostatic/compensatory hypertrophy state and a failing state, respectively. Intercellular communication analysis revealed that cardiomyocytes, particularly the failing subpopulation CM2, exhibited reduced communication between themselves (autocrine) and with some other lineages (paracrine) in HCM (Fig. 5c, d), indicating communication dysfunction of cardiomyocytes in HCM. Previous single-nucleus/cell studies on human cardiac tissues may report different numbers of cardiomyocyte subpopulations due to different resolutions applied for clustering across studies^9,18,29^. However, all these studies and ours suggested that the transcriptomic states of cardiomyocytes in human hearts were continuous rather than discrete, and an *NPPB*/*NPPA* ^high^ cardiomyocyte subpopulation existed especially under diseased conditions. Our spatial transcriptomics revealed that the *NPPB* ^high^ cardiomyocyte subpopulation was close to the fibrotic regions (Fig. 6e), reflecting the detrimental effects of cardiac fibrosis on cardiomyocytes.

Pathological cardiac hypertrophy is a common predecessor to heart failure^30^. A recent study discovered the transcriptomic differences in cardiomyocytes between early (hypertrophic cardiomyocytes) and maladaptive phage (failing cardiomyocytes) of cardiac remodeling in pressure overload-induced mouse models^31^. In the present study, the transcriptomic dynamics during the transition towards the failing state of cardiomyocytes in human HCM patients were identified using pseudo-temporal ordering (Fig. 2i). A list of potential key genes during the transition towards a failing state of cardiomyocytes was obtained based on multiple lines of evidence from independent analyses (Fig. 2j), the majority of which have not been implicated in heart failure or cardiac hypertrophy before, such as *FGF12* and *CREB5*. Notably, the expression of *FGF12* in HCM decreased along the trajectory towards the failing state (Fig. 2k). Recently, *FGF12* has been reported to inhibit the pathological remodeling of SMCs in pulmonary arterial hypertension^32^. Similarly, it may protect cardiomyocytes from aberrant remodeling and failure in HCM. A recently published single-nucleus atlas of pressure overload-induced cardiac hypertrophy (caused by aortic valve stenosis) reported that *FGF12* was downregulated in hypertrophic cardiomyocytes, and incoming signals from other lineages had been reduced in hypertrophied cardiomyocytes^29^. Our findings were consistent with these reports and implied that some conserved molecular alterations existed among different types of cardiac hypertrophy.

Cardiac fibrosis is a scarring process that occurs in the cardiac tissue characterized by excessive ECM deposition in response to pathophysiological stimuli^33^. HCM patients suffer from a high burden of cardiac fibrosis^34^ which leads to diastolic dysfunction. Cardiac fibrosis has been suggested to be an independent predictor of adverse outcomes in HCM patients, including SCD and heart failure^35^. Cardiac fibrosis is mediated by fibroblast activation. Understanding the regulatory mechanism underlying fibroblast activation in HCM is critical for developing effective medical therapies to alleviate cardiac fibrosis and, as a result, prevent adverse outcomes in HCM patients. The activated fibroblast subpopulation FB2 was found to be significantly expanded in HCM (Fig. 3e) and located in fibrotic regions (Fig. 6e) as expected. Furthermore, a total of 28 potential key genes were identified for anti-fibrosis medical development based on multiple lines of evidence from independent analyses (Fig. 3k). Some of the top-ranked TF genes may be major regulators implicated in cardiac fibroblast activation. For example, a recent study demonstrated that *MEOX1* regulates the pro-fibrotic function and is implicated in the fibrosis of multiple human organs, including the heart, liver, lung, and kidney^36^. *AEBP1* (also named as *ACLP*) was first identified as a transcriptional repressor that regulates adipogenesis^37^. *AEBP1* has been demonstrated to enhance lung myofibroblast differentiation in mice^38^. For cardiac fibrosis, the expression of *AEBP1* has been reported to be associated with fibrosis in DCM^39^. However, the roles of *AEBP1* in cardiac fibrosis have yet to be experimentally determined. In this study, we showed that in vitro knockdown of *AEBP1* could promote the activation of human cardiac fibroblasts (Fig. 7), while overexpression of *AEBP1* could attenuate the TGFβ-induced activation (Fig. 8). These results suggest that *AEBP1* may function as a transcription repressor in cardiac fibroblast activation, and reflect a protective role of the upregulation of *AEBP1* in HCM fibroblasts. Thus, overexpressing *AEBP1* in cardiac tissues may provide a novel therapeutic strategy to attenuate cardiac fibrosis. However, detailed regulatory mechanisms of *AEBP1* remain to be elucidated and in vivo studies are needed to confirm *AEBP1* as a potential therapeutic target. Moreover, we discovered an array of candidate genes that have not been explicitly implicated in cardiac fibrosis before. For example, *NRXN3*, which encodes a transmembrane receptor protein of the neurexin family that is predominantly expressed in neurons and mostly discussed in mental diseases^40^, was found to be highly expressed in activated fibroblasts (Fig. 3b), and its precise role in cardiac fibrosis warrants further investigation.

We made a comparison of the results of an snRNA-seq study on DCM^18^ and ours on HCM. Despite the large differences in clinical information of the enrolled patients (e.g., DCM vs HCM) and tissue sampling sites (left ventricle vs interventricular septum) between the two studies, we found similar results including the expanded/contracted subpopulations in diseased states and the key dysregulated genes. For example, ten fibroblast subpopulations (Fb1-Fb10) were reported in the DCM study, among which, Fb5 and Fb8 were reported to be expanded in DCM, while Fb3 and Fb4 were contracted^18^. We calculated the expression score of the molecular signature for each fibroblast subpopulations in our HCM dataset as a proxy of relative proportion. We found that these DCM-associated subpopulations were also expanded/contracted in HCM (Supplementary Fig. S7d). In addition, the expression of the representative dysregulated genes in DCM fibroblasts was found to be also significantly changed in HCM (Supplementary Fig. S7e). We also observed that the expression of the potential key genes in cardiac fibrosis that we prioritized were significantly altered in DCM (Supplementary Fig. S7f), for example, the top candidate gene *AEBP1*. Likewise, cardiomyocytes exhibited similar changes between HCM and DCM (Supplementary Fig. S7a–c). These results reflect convergent changes in cellular states across different types of cardiomyopathies, particularly at an advanced stage.

Increasing evidence suggests that immune cells coordinate the responses of cardiomyocytes (e.g., hypertrophy) and other noncardiomyocytes (e.g., fibroblast activation) during pathological cardiac remodeling^41^. Therefore, identifying disease-associated immune cell subpopulations and developing therapeutics to regulate the phenotype of cardiac immune cells, for example, targeting cardiac fibrosis with engineered T cells^42^, represent another important treatment option. We investigated the alterations in the immune microenvironment of HCM cardiac tissue and found the activation of both innate (e.g., tissue-resident macrophages) and adaptive (e.g., T/NK cells) immunity (Fig. 4). Meanwhile, immune response-related pathways, for example, antigen processing and presentation, were found to be upregulated in all the nonimmune cell types, indicating an enhanced immune response in HCM. TGFβ signaling has several pleiotropic effects not only in disease, for example, promoting cardiac hypertrophy and fibrosis in pathological cardiac remodeling, but also in tissue homeostasis^43^. While TGFβ blockade may be a promising therapeutic strategy, direct and excessive TGFβ inhibition may lead to matrix degradation, cardiac dilation, and dysfunction^44^. Through intercellular communication analysis, we found that the top sender of TGFβ changed from MAC2 in HEALTHY to effector T/NK cells in HCM (Fig. 5j), implying that inhibiting T/NK cell activation may attenuate TGFβ signaling and thus alleviate pathological remodeling in HCM while avoiding the deleterious effects of direct TGFβ blockade.

The HEALTHY group (*n* = 2; 3 samples) had fewer subjects than the HCM group (*n* = 10; 10 samples) for snRNA-seq in the current study, which may reduce the power of comparative analyses if the HEALTHY group had much fewer nuclei. To overcome this limitation, we increased the number of sequenced nuclei (15,939) in the HEALTHY group and expanded the sample size of the HEALTHY group (*n* = 5) in the western blot validation. Nevertheless, given the heterogeneity of human tissue samples, the current study was still limited by a relatively small sample size. Although the transcriptomic dynamics during the progression of pathophysiological changes could be inferred through pseudotime ordering, the information derived in this study was mainly related to the advanced stage of HCM. Only TFs, ligands, and receptors were considered in the prioritization of potential key genes for subsequent functional studies in our lab. However, other types of molecules may also serve as ideal targets for drug development. Analysis results for all the genes are supplied in supplementary tables for further prioritization. In addition, extensive in vivo and in vitro experiments are needed to confirm the roles of the prioritized key genes.

In conclusion, the present study provides a comprehensive analysis of the lineage-specific regulatory changes in HCM. Our analysis identified potential key genes during the transition towards a failing state of cardiomyocytes or during the activation of fibroblasts in HCM. We showed experimental evidence supporting that AEBP1 functions as a transcription repressor in cardiac fibroblast activation. The datasets constitute a valuable resource to investigate cell type-specific expression in HCM at single-nucleus and spatial resolution.

## Materials and methods

### Ethics approval

All study procedures complied with the ethical regulations approved by the Ethics Committee of Fuwai Hospital, the Chinese Academy of Sciences (No. 2020-1315). Written informed consent was received from each patient.

### Study subjects and cardiac tissue collection

We enrolled HCM patients (*n* = 16) who had undergone surgical myectomy in Fuwai Hospital between 2015 and 2021. The inclusion criteria were as follows: (i) patients who met the diagnostic criteria^45^ for HCM with a maximal left ventricular wall thickness ≥15 mm or ≥13 mm in patients with a family history of HCM; (ii) patients with the basal septum subtype, the most common and severe morphological subtype^4^, in which cardiac hypertrophy mainly confines to the basal IVS adjacent to the aortic valve; (iii) patients exhibited left ventricular outflow tract (LVOT) obstruction (LVOT gradient ≥ 30 mm Hg at rest or on provocation). The exclusion criteria were as follows: (i) patients with cardiac hypertrophy caused by secondary factors, including systemic hypertension, myocardial infarction, valvular disease, or hemodynamic obstruction ascribed to left-sided obstructive lesions (e.g., valvular stenosis); (ii) patients with myocarditis and systemic disorders such as RASopathies, mitochondrial myopathies and storage diseases. For snRNA-seq, cardiac IVS tissues isolated from HCM patients (*n* = 10) during surgical resection were immediately frozen and stored in liquid nitrogen until use for nuclei isolation. For spatial transcriptomic assays, fresh cardiac IVS tissues from HCM patients (*n* = 6) were concurrently frozen in isopentane precooled by liquid nitrogen and embedded in the optical cutting tissue (OCT) compound. Cardiac IVS tissues obtained from healthy donors of heart transplants (*n* = 2) were used as a control for snRNA-seq.

### Human cardiac fibroblast culture

Normal Human Ventricular Cardiac Fibroblasts (NHCF-V, CC-2904, Lonza) were cultured according to the recommended protocol. In brief, cells were grown in FBM^TM^ Basal Medium (CC-3131, Lonza) supplemented with FGM^TM^-3 SingleQuot (CC-4525, Lonza) and 1× penicillin-streptomycin (Life Technologies) at 37 °C in a humidified atmosphere containing 5% CO_2_.

### Nucleus isolation

Frozen myocardial tissue was thawed on ice and dissected into small pieces, and washed once using cold PBS (20012050, Gibco). Tissue pieces were then transferred into a 50 mL Falcon tube containing 30 mL of lysis buffer (0.32 M sucrose, 5 mM CaCl_2_, 3 mM C_4_H_6_MgO_4_, 0.5 mM EGTA, 10 mM Tris-HCl 8.0, 2 mM EDTA, 1 mM PMSF, 1 mM DTT, and 80 U/mL RI). Tissues were homogenized with a T-25 Ultra-Turrax probe homogenized (IKA) at 24,000 rpm for 15 sec. A glass douncer (40 mL) with a tight pestle was used to homogenize the tissue with 10 strokes. After a 10 min-incubation on ice, the crude nucleus suspension was passed through a 100 μm and 70 μm nylon mesh cell strainer (BD Biosciences) and subsequently spun down with a centrifuge (700× *g* for 10 min at 4 °C). The supernatant was removed carefully. The crude nucleus isolated was suspended in 30 mL sucrose buffer (2.1 M Sucrose, 3 mM C_4_H_6_MgO_4_, 10 mM Tris-HCl 8.0, 1 mM PMSF, 1 mM DTT, and 80 U/mL RI), and centrifuged (13,000 rpm for 60 min) at 4 °C (Beckman Avanti S-25). The supernatant was carefully removed, and the nucleus pellet was dissolved in 10 mL nucleus suspension buffer (1% BSA, 200 U/mL RI in PBS), and centrifuged (500× *g* for 10 min) at 4 °C. The nucleus pellet was suspended in nucleus suspension buffer (1 mL) and used in further experiments. All procedures were performed at 4 °C. RNA inhibitor (80 U/mL, 2313B, Takara) was added to all buffers.

### Library preparation for snRNA-seq

The prepared single nuclei suspension was loaded on the Chromium Controller (10× Genomics). We prepared 3' gene expression libraries using Chromium Next GEM Single Cell 3' GEM, Library & Gel Bead Kit v3.1 according to the manufacturer’s protocol. The libraries were sequenced on an Illumina NovaSeq 6000 system.

### Preprocessing and quality control of the snRNA-seq data

To count the exonic and intronic reads captured by snRNA-seq, we built a custom “pre-mRNA” reference package based on the human reference genome dataset (version: refdata-gex-GRCh38-2020-A) following the protocol for 10× Genomics. The raw sequencing reads were aligned to the “pre-mRNA” reference using the official toolkit Cell Ranger (v4.0.0). For further data preprocessing, the output nucleus-gene expression matrix was imported into Seurat (v3.2.3)^46^. Genes with counts in fewer than 3 nuclei were filtered out to exclude genes likely detected due to random noise. Nuclei were filtered for unique molecular identifier (UMI) counts (500 < nCount_RNA < 50,000), genes (300 < nFeature_RNA < 7000), the proportion of mitochondrial genes (percent.mito < 0.05) and the proportion of ribosomal genes (percent.ribo < 0.05) to remove poor-quality nuclei potentially ascribed to doublets or other technical noise. To further remove possible doublets, nuclei with the doublet scores > 0.35 predicted by Scrublet^47^ were filtered out. In addition, nuclei enriched in the expression of marker genes for multiple lineages were excluded from further analyses.

### Normalization, feature selection, integration, scaling, and clustering of the snRNA-seq data

For each sample, the sum of the UMI counts for each nucleus was normalized to 10,000, and then log-transformed. Using the “FindVariableFeatures” function of Seurat, we selected 2000 genes for each sample. Nuclei of all samples were integrated via canonical correlation analysis implemented in Seurat to correct for potential batch effects and identify shared cell states across samples. We also mitigated the effects of unwanted sources of variation by regressing out the proportion of mitochondrial genes, UMI count, gene number, the proportion of mitochondrial genes, and the proportion of ribosomal genes with linear models using the “ScaleData” function. Subsequently, the data were centered for each gene by subtracting the average expression of that gene across all nuclei and then scaled by dividing the centered expression by the standard deviation. The scaled data were subjected to linear dimensional reduction through principal component analysis (PCA). Using the first 30 PCA components, we computed a shared nearest neighbor graph of the nuclei. The SNN graph was embedded in two-dimensional space using a non-linear dimensional reduction method, that is, UMAP. All nuclei were clustered using the Louvain algorithm.

### Identification of the differentially expressed genes in a specific cell type based on the snRNA-seq data

The differentially expressed genes in a specific cell type between HCM and HEALTHY were detected through differential expression analysis using a method implemented in the R package DEsingle^13^ employing a zero-inflated negative binomial model to estimate the fraction of dropout and real zeros in snRNA-seq data. A gene was significantly differentially expressed if it met the following criteria: the absolute of log2 fold change > 1, adjusted *P*-value < 0.05, and being categorized as “general differential expression”, which means that the gene is significantly different in both the expression abundance and the fraction of real zeros between HCM and HEALTHY.

### Pseudobulk RNA-seq analysis

For each cell type, the raw UMI count matrix of the snRNA-seq data was summed per gene for each sample into a pseudobulk RNA-seq dataset. Differential expression analysis of the pseudobulk RNA-seq dataset was performed using the R package DESeq2 under default settings. The statistical significance was set to a *P*-value adjusted for multiple tests < 0.05.

### GSEA based on the snRNA-seq data

Before GSEA, all the genes expressed in the snRNA-seq data were pre-ranked by Signal2Noise (the difference of means between HCM and HEALTHY scaled by the standard deviation). The ranked gene list was imported into the GSEA software (version: 4.0.1). An FDR *q*-value < 0.05 were set to be statistically significant. The precompiled canonical pathway gene sets (“c2.cp”) in MSigDB (version: 7.2) were used in this analysis.

### Differential gene regulatory network analysis based on the snRNA-seq data

GRNs in a specific lineage were built based on the single-nucleus datasets and a comparative analysis of the GRNs between HCM and HEALTHY was performed using the method implemented in bigScale2^14^. Briefly, the GRN of a specific lineage was inferred with the ‘compute.network’ function separately for each condition. The ‘homogenize.networks’ function was applied to homogenize the number of edges of the inferred GRNs throughout the networks. Changes in node centralities (the relative importance of genes in the network) in HCM versus HEALTHY were identified using the ‘compare.centrality’ function. Gene rankings based on the changes in centrality were output separately for each of the four measures of centrality (degree, betweenness, closeness, and pagerank). The networks were visualized with Cytoscape (version: 3.7.1).

### Trajectory inference based on the snRNA-seq data

Trajectory inference was performed to order the nuclei along a biological process of interest, e.g., fibroblast activation, using Slingshot (v1.4.0)^15^ under default settings. The combined snRNA-seq data of HCM and HEALTHY was considered to increase the robustness of the inference. Using the Kolmogorov–Smirnov test, we assessed whether differences existed between the pseudotime distributions of the two conditions. Subsequently, genes with different expression patterns along the trajectory between the two conditions were identified by tradeSeq (v1.6.0)^16^. For each condition, a negative binomial generalized additive model (the function “fitGAM”, nknots = 5) was applied to estimate a smoothed expression profile along the inferred trajectory for each gene. The fitted model acted as an input to the “conditionTest” function to test whether genes exhibited different expression patterns along the trajectory between conditions (referred to as differential expression pattern analysis). The significance threshold was set to a *P*-value adjusted for multiple testing < 0.05.

### Ligand-receptor interaction analysis based on the snRNA-seq data

CellChat (v0.5.5)^25^ was used to infer ligand-receptor interactions among subpopulations separately for each condition and to identify the signaling changes in HCM via comparative analysis by following tutorials of the software. Briefly, following the detection of overexpressed ligands or receptors for each subpopulation, each potential communication between any two subpopulations was quantified using a communication probability (interaction strength) value, modeled by the law of mass action. Significant interactions (*P*-value < 0.05) were determined via a permutation test by randomly permuting the subpopulation labels and recalculating the communication probability. Through leveraging pattern recognition approaches, dominant incoming and outgoing signal patterns for each subpopulation were detected in HCM or HEALTHY. Major signaling sources and targets of the signaling network for a specific pathway were inferred through network centrality analysis. Joint manifold learning of the communication networks of HCM and HEALTHY was performed to group the signaling pathways according to functional similarity (a high degree of functional similarity implied that the major senders and receivers are similar). Signaling pathways with pronounced changes in terms of functional similarity in HCM versus HEALTHY were identified based on the Euclidean distance in the learned joint manifold. The conserved or greatly changed signaling pathways in HCM were identified by comparing the overall information flow (the sum of communication probability among all pairs of cell groups in the inferred network) of each signaling pathway in HCM versus HEALTHY.

### Sample preparation for spatial transcriptomic assays

The RNA quality of the OCT-embedded cardiac IVS tissue block was evaluated using an Agilent 2100 bioanalyzer. Tissue blocks with an RNA integrity number greater than 6 were used for 10× Visium spatial transcriptomic assays. Cryosectioning was performed on a Leica CM3050S cryostat to generate 10 μm tissue sections. Brightfield images were captured using a Leica Aperio VERSA whole-slide scanner at 20× resolution.

### Tissue optimization for spatial transcriptomic assays

The conditions for tissue permeabilization were optimized according to the 10× Visium Spatial Tissue Optimization User Guide (CG000238, 10× Genomics). Briefly, tissue sections placed on the capture areas of a tissue optimization slide were fixed, stained, and permeabilized. mRNA released during permeabilization bound to oligonucleotides on the capture areas. Images of the fluorescent cDNA synthesized on the slide were taken. The optimal permeabilization time contributed to the maximum fluorescence signal and the lowest signal diffusion.

### Preparation of sequencing libraries for spatial transcriptomic assays

Sequencing libraries were constructed using the Visium Spatial Gene Expression Slide & Reagent kit (1000187, 10× Genomics) following the manufacturer’s instructions. Briefly, a frozen tissue section (10 μm) was placed on a capture area (6.5 × 6.5 mm with ~5000 barcoded spots) of a gene expression slide, then stained with hematoxylin and eosin (H&E). Brightfield images were taken. The tissue was permeabilized for the optimal time as described above. Then, reverse transcription was done and sequencing libraries were prepared. Sequencing was conducted using an Illumina NovaSeq 6000 system.

### Processing of the spatial transcriptomic data

Sequencing read alignment, fiducial/tissue detection, and spot barcode/UMI counting of the spatial transcriptomic data were performed separately for each section using the 10× Genomics official tool kit Space Ranger (v1.2.2) with an H&E-stained brightfield image and fastq files as inputs. The same version of the human reference genome dataset as that used to process snRNA-seq data was applied, i.e., refdata-gex-GRCh38-2020-A. The output gene-spot matrix was imported to Seurat for downstream analysis and visualization. Only spots that have been determined to be over tissue were retained. To suppress technical artifacts while preserving biological variance in UMI counts across spots, data were normalized using the method sctransform^48^. Linear dimensional reduction with PCA was performed using the “RunPCA” function. An SNN graph was constructed according to the first 30 PCA components using the “FindNeighbors” function. The SNN graph allowed for the clustering of spots by the Louvain algorithm (resolution: 0.4) using the “FindClusters” function. Lastly, UMAP dimensional reduction was performed using the “RunUMAP” function to visualize the spots in a 2D space.

### Identification of the molecular signature for each nucleus cluster/spot cluster

The molecular signature of each nucleus/spot cluster was obtained by comparing the transcriptome of each cluster with that of the other clusters using a likelihood-ratio test (test.use: “bimod”) implemented in the “FindMarkers” function of Seurat. The significance threshold was set to an adjusted *P*-value < 0.05 and a log2 fold change > 0.25.

### Pathway activity scoring in each nucleus/spatial transcriptomic spot

The expression activity of a given gene set/pathway in each nucleus/ST spot was quantified by calculating the gene set/pathway activity score for each nucleus or spot using the method implemented in Single Cell Signature Explorer^49^. The precompiled canonical pathway gene sets (“c2.cp”) in MsigDB were applied in this analysis. In each sample, the expression activity of a pathway was represented by the mean of the computed scores across the nuclei/spots in the sample. According to the calculated pathway activity score of each spatial transcriptomic spot, the differentially regulated pathways of the spots in fibrotic versus non-fibrotic regions of cardiac tissue sections were detected using the Wilcoxon rank-sum test implemented in the “FindMarkers” function of Seurat under default settings. The significance threshold was set to a Bonferroni-adjusted *P*-value < 0.05 and an absolute of the log2 fold change > 0.25.

### Integration of the spatial transcriptomic data with the snRNA-seq data

To integrate the spatial transcriptomic data with the snRNA-seq data and predict the underlying cellular composition for each spot that contained multiple nuclei, we applied the label transfer workflow of Seurat to assign each spot prediction score for the subpopulations obtained from the snRNA-seq data analysis.

### Spot-level cellular composition visualization

Following the label transfer, the spot-level cellular composition of the spatial transcriptomic data was visualized with the st.pl.deconvolution_plot function of stLearn (v0.3.1, https://stlearn.readthedocs.io/en/latest/index.html). Noise labels were filtered for better visualization based on quantile (threshold = 0.5).

### Spatial pseudotime analysis

To decipher the transcriptomic dynamics during the change from non-fibrotic to fibrotic states of cardiac tissues in HCM, pseudotime ordering of the spots of the spatial transcriptomic data was performed using Monocle3 (https://cole-trapnell-lab.github.io/monocle3/). Spatial spot cluster enriched with hemoglobin genes was excluded from this analysis. The spots were subjected to dimension reduction with UMAP, and trajectory inference using the order_cells function. Then, the spots were ordered in pseudotime with the order_cells function. The plotSurface function of SPATA2 (https://themilolab.github.io/SPATA2/index.html) was used to visualize the pseudotime in a spatial context. Lastly, the graph_test of Monocle3 was used to identify genes that significantly change as a function of the pseudotime. Only genes that met the following threshold were considered as significantly changed genes: *q* value < 0.05 and morans I > 0. Only the section HCM1225D was considered for spatial pseudotime analysis because it represents a typical section with a clear separation of fibrotic and non-fibrotic regions.

### Spatial cell-cell interaction analysis

CCI analysis was performed using stLearn (v0.3.1) by integrating known ligand-receptor pair information, spatial cell-type distribution, and spatial gene expression. Tissue regions with high cellular diversity and ligand-receptor co-expression activities were considered as hotspots with high CCI activities. A CCI score was calculated (the “st.tl.cci.merge“ function) to measure the CCI activity of each spot, which combines cellular diversity and ligand-receptor co-expression among neighboring spots (between-spots mode) or within spots (within-spots mode). To find hotspots, all 475 ligand-receptor pairs that were expressed in the cardiac tissues of HCM predicted by CellChat were considered.

### Regulon analysis

Regulon analysis was performed using the R package SCENIC under default settings (https://github.com/aertslab/SCENIC). Briefly, gene co-expression modules in a specific cell type were detected. Then, only the modules with significant motif enrichment of TFs were retained and referred to as regulons. For the TF motif enrichment analysis, two databases were used: “hg38__refseq-r80__500bp_up_and_100bp_down_tss.mc9nr.feather” and “hg38__refseq-r80__10kb_up_and_down_tss.mc9nr.feather”. Lastly, the activity of each regulon was scored for each nucleus. Activity scores were compared between HCM and HEALTHY for each regulon, and the statistical significance threshold was set to a Bonferroni-adjusted *P*-value of Wilcoxon rank-sum test < 0.05.

### Functional enrichment analysis

Functional enrichment analyses of a list of genes were performed using ClueGO with a Bonferroni corrected *P*-value threshold of 0.05. Databases, including Gene ontology biological process, REACTOME, and KEGG were considered in this analysis.

### Bulk RNA-seq analysis

Bulk RNA-seq data analysis was performed by following the procedure described in a previous study^50^. Briefly, the differential expression analysis was performed using the R package sleuth. The statistical significance threshold was set to a *q*-value < 0.05, and the biological significance threshold was set to an absolute log2 fold-change > 0.5.

### Masson’s trichrome staining

To histologically assess cardiac fibrosis, Masson’s trichrome staining was performed on the tissue sections adjacent to those used for spatial transcriptomic assays. Masson’s Trichrome stain kit (NO.850, Beijing Yili Fine Chemicals Co., Ltd.) was used by following the manufacturer’s protocol. Briefly, the frozen sections were incubated in 0.5% hydrochloric acid alcohol for 5 s and then stained in Weigert’s Iron Hematoxylin Solution for 2 min. Next, the sections were stained in Ponceau Fuchsin for 5 min and incubated in the phosphomolybdic acid solution for 2 min. Subsequently, the sections were stained in Brilliant Green solution for 2 s, incubated in 1% Glacial acetic acid for 1 min, and then placed in xylene for 5 min. Images were captured using a Pannoramic SCAN II scanner (3DHISTECH).

### Western blot assays

The myocardium tissue in liquid nitrogen was ground into powder and then transferred to a 1.5 mL centrifuge tube. After that, four volumes (μL/mg) of lysis buffer (1% Triton X-100, 1% protease inhibitor, and 1% phosphatase inhibitor) were added to the powder and incubated for 1 h on Rotater at 4 °C. For cultured cells, cells were lysed in lysis buffer for 30 min. Supernatants were collected by centrifugation (12,000× *g* × 15 min at 4 °C). Then, the protein concentration was determined with BCA Protein Assay Kit according to the manufacturer’s instructions (23225, Thermo Fisher Scientific). Protein samples (20 µg/lane) were electrophoresed on a 4%–20% SDS polyacrylamide gel (P0468S and P0469S, Beyotime), and transferred onto a nitrocellulose membrane (66485, PALL). Membranes were blocked with 5% milk in TBST for 1.5 h and incubated with primary antibody (Supplementary Table S18) overnight at 4 °C. Membranes were washed 3 times with TBST and then incubated with HRP-labeled species-specific secondary antibodies (Supplementary Table S18) for 1 h. Then, membranes were washed 3 times with TBST. Blots were developed using BeyoECL HRP substrate (P0018AM, Beyotime) in a ChemiDoc XRS (Bio-Rad) image acquisition system. Quantitative densitometry analysis of each band using Image J processing software.

### Immunofluorescence staining

Myocardial tissue was fixed in 10% formalin, and processed for paraffin sectioning. 4 µm sections were dewaxed by immersion in xylene and hydrated by serial immersion in ethanol and deionized water. Antigen retrieval was performed by incubating sections in a pressure cooker for 15 min in Antigen Retrieval Buffer (ZLI-9069, ZSGB-BIO) at pH 9.0. Sections were washed with PBS (three times for 5 min), and then a blocking buffer (ZLI-9056, ZSGB-BIO) was added. Primary antibody dilutions were prepared in antibody diluent (ZLI-9028, ZSGB-BIO) and incubated overnight at 4 °C in a moist chamber. Secondary antibodies (Supplementary Table S18) diluted in 1% PBS were then added (1:200 dilution). Sections were mounted with a fluorescent mounting medium with DAPI (ZLI-9557, ZSGB-BIO). Slides were viewed with a Pannoramic SCAN II scanner (3DHISTECH) using suitable filter combinations provided by the manufacturer.

For cultured cells, cells were fixed in 4% paraformaldehyde and then permeabilized in 0.2% Triton-1× PBS. Subsequently, cells were blocked with 3% BSA in PBS for 1 h at room temperature and then incubated overnight at 4 °C with primary antibodies. Then the cells were stained with primary antibodies targeting Collagen-I (ab138492, Abcam). Nuclei were stained with DAPI (4083S, Cell Signaling Technology). Fluorescence intensity was analyzed by ImageJ.

### siRNA transfection

Human cardiac fibroblasts (passage 5–7, 8 × 10^4^) were seeded before the day of transfection in 6-well plates. On the day of transfection, cells were transfected with 50 nM human *AEBP1* siRNA or 50 nM scramble siRNA as a negative control according to the manufacturer’s instruction (stB0002601A, Ribobio). Briefly, 6 μL Dharmafect 1 transfection reagent (T-2001-01, GE Healthcare Dharmacon) was used for each well. Six hours after transfection, the cells were cultured in a maintenance medium with 2% FBS. Total RNA was isolated 48 h post-transfection and mRNA was prepared for bulk RNA-Seq analysis. Proteins were extracted 72 h post-transfection and the protein levels were analyzed by western blot.

### Adenoviral-mediated gene overexpression

Human cardiac fibroblasts (passage 5-6, 8 × 10^4^/well) were seeded before the day of transfection in 6-well or 24-well plates. Cells (30%–50% confluent) were infected with adenovirus-vector-GFP or adenovirus-*AEBP1*-GFP (HanBio, HH20220630GX-AD02) in half volume medium at a multiplicity of infection (MOI) 400 or 300, respectively at 37 °C for 4 h. Then, the medium was changed to a full-volume fresh culture medium. Eight hours later, cells were starved in 0.5% FBS for 12 h. Subsequently, cells were treated with TGFβ (6 ng/L, 100-21C-50UG, Peprotech) or vehicle for 48 h. Then protein was extracted and the protein levels were analyzed by western blot assay.

### Phalloidin staining

Cells were treated with rhodamine-phalloidin (1:400, R415, Thermo Fisher) for 40 min at room temperature. Nuclei were stained with DAPI (4083S, Cell Signaling Technology).

## Supplementary information

Supplementary Information

Table S1

Table S2

Table S3

Table S4

Table S5

Table S6

Table S7

Table S8

Table S9

Table S10

Table S11

Table S12

Table S13

Table S14

Table S15

Table S16

Table S17

Table S18

## Data Availability

The datasets generated and/or analyzed during the current study are available in Figshare (10.6084/m9.figshare.c.5777948.v2).

## References

[1] Semsarian C, Ingles J, Maron MS, Maron BJ (2015). New perspectives on the prevalence of hypertrophic cardiomyopathy. J. Am. Coll. Cardiol..

[2] Wasfy MM, Hutter AM, Weiner RB (2016). Sudden cardiac death in athletes. Methodist Debakey Cardiovasc. J.

[3] Wolf CM (2019). Hypertrophic cardiomyopathy: genetics and clinical perspectives. Cardiovasc. Diagn. Ther..

[4] Marian AJ, Braunwald E (2017). Hypertrophic cardiomyopathy: Genetics, pathogenesis, clinical manifestations, diagnosis, and therapy. Circ. Res..

[5] Burchfield JS, Xie M, Hill JA (2013). Pathological ventricular remodeling: Mechanisms: Part 1 of 2. Circulation.

[6] Santini L, Palandri C, Nediani C, Cerbai E, Coppini R (2020). Modelling genetic diseases for drug development: Hypertrophic cardiomyopathy. Pharmacol. Res..

[7] Liu X (2019). Long non-coding and coding RNA profiling using strand-specific RNA-seq in human hypertrophic cardiomyopathy. Sci. Data.

[8] Ren CW (2016). RNA-seq profiling of mRNA associated with hypertrophic cardiomyopathy. Mol. Med. Rep..

[9] Litviňuková M (2020). Cells of the adult human heart. Nature.

[10] Marx V (2021). Method of the Year: spatially resolved transcriptomics. Nat. Methods.

[11] Feng W, Chen L, Nguyen PK, Wu SM, Li G (2019). Single cell analysis of endothelial cells identified organ-specific molecular signatures and heart-specific cell populations and molecular features. Front. Cardiovasc. Med.

[12] Nakamura M, Sadoshima J (2018). Mechanisms of physiological and pathological cardiac hypertrophy. Nat. Rev. Cardiol..

[13] Miao Z, Deng K, Wang X, Zhang X (2018). DEsingle for detecting three types of differential expression in single-cell RNA-seq data. Bioinformatics.

[14] Iacono G, Massoni-Badosa R, Heyn H (2019). Single-cell transcriptomics unveils gene regulatory network plasticity. Genome Biol..

[15] Street K (2018). Slingshot: Cell lineage and pseudotime inference for single-cell transcriptomics. BMC Genomics.

[16] Van den Berge K (2020). Trajectory-based differential expression analysis for single-cell sequencing data. Nat. Commun..

[17] Aibar S (2017). SCENIC: Single-cell regulatory network inference and clustering. Nat. Methods.

[18] Koenig AL (2022). Single-cell transcriptomics reveals cell-type-specific diversification in human heart failure. Nat. Cardiovasc. Res..

[19] Ivey MJ, Tallquist MD (2016). Defining the cardiac fibroblast. Circ. J..

[20] Hocker JD (2021). Cardiac cell type-specific gene regulatory programs and disease risk association. Sci. Adv..

[21] Hu L, Lin X, Lu H, Chen B, Bai Y (2015). An overview of hedgehog signaling in fibrosis. Mol. Pharmacol..

[22] Salazar NC, Chen J, Rockman HA (2007). Cardiac GPCRs: GPCR signaling in healthy and failing hearts. Biochim. Biophys. Acta - Biomembr..

[23] Lim HY (2018). Hyaluronan receptor LYVE-1-expressing macrophages maintain arterial tone through hyaluronan-mediated regulation of smooth muscle cell collagen. Immunity.

[24] Yang Q (2021). Single-cell RNA sequencing reveals the heterogeneity of tumor-associated macrophage in non-small cell lung cancer and differences between sexes. Front. Immunol.

[25] Jin S (2021). Inference and analysis of cell-cell communication using CellChat. Nat. Commun..

[26] Liu X (2022). Single-cell RNA-sequencing reveals lineage-specific regulatory changes of fibroblasts and vascular endothelial cells in keloids. J. Invest. Dermatol.

[27] Kumarapeli ARK (2008). αB-crystallin suppresses pressure overload cardiac hypertrophy. Circ. Res.

[28] Ritterhoff J, Most P (2012). Targeting S100A1 in heart failure. Gene Ther..

[29] Nicin L (2022). A human cell atlas of the pressure-induced hypertrophic heart. Nat. Cardiovasc. Res..

[30] Ren Z (2020). Single-cell reconstruction of progression trajectory reveals intervention principles in pathological cardiac hypertrophy. Circulation.

[31] Vigil-Garcia M (2021). Gene expression profiling of hypertrophic cardiomyocytes identifies new players in pathological remodelling. Cardiovasc. Res..

[32] Yeo Y (2020). FGF12 (Fibroblast Growth Factor 12) inhibits vascular smooth muscle cell remodeling in pulmonary arterial hypertension. Hypertension.

[33] Travers JG, Kamal FA, Robbins J, Yutzey KE, Blaxall BC (2016). Cardiac fibrosis: The fibroblast awakens. Circ. Res..

[34] Galati G (2016). Histological and histometric characterization of myocardial fibrosis in end-stage hypertrophic cardiomyopathy. Circ. Hear. Fail..

[35] O’Hanlon R (2010). Prognostic significance of myocardial fibrosis in hypertrophic cardiomyopathy. J. Am. Coll. Cardiol..

[36] Alexanian M (2021). A transcriptional switch governs fibroblast activation in heart disease. Nature.

[37] He GP, Muise A, Wu Li A, Ro HS (1995). A eukaryotic transcriptional represser with carboxypeptidase activity. Nature.

[38] Tumelty KE, Smith BD, Nugent MA, Layne MD (2014). Aortic carboxypeptidase-like protein (ACLP) enhances lung myofibroblast differentiation through transforming growth factor β receptor-dependent and -independent pathways. J. Biol. Chem..

[39] Rao M (2021). Resolving the intertwining of inflammation and fibrosis in human heart failure at single-cell level. Basic Res. Cardiol..

[40] Tromp A, Mowry B, Giacomotto J (2021). Neurexins in autism and schizophrenia—a review of patient mutations, mouse models and potential future directions. Mol. Psychiatry.

[41] Frieler RA, Mortensen RM (2015). Immune cell and other noncardiomyocyte regulation of cardiac hypertrophy and remodeling. Circulation.

[42] Aghajanian H (2019). Targeting cardiac fibrosis with engineered T cells. Nature.

[43] Bujak M, Frangogiannis NG (2007). The role of TGF-β signaling in myocardial infarction and cardiac remodeling. Cardiovasc. Res..

[44] Dobaczewski M, Chen W, Frangogiannis NG (2011). Transforming growth factor (TGF)-β signaling in cardiac remodeling. J. Mol. Cell. Cardiol..

[45] Ommen SR (2020). 2020 AHA/ACC guideline for the diagnosis and treatment of patients with hypertrophic cardiomyopathy. Circulation.

[46] Stuart T (2019). Comprehensive integration of single-cell data. Cell.

[47] Wolock SL, Lopez R, Klein AM (2019). Scrublet: computational identification of cell doublets in single-cell transcriptomic data. Cell Syst.

[48] Hafemeister C, Satija R (2019). Normalization and variance stabilization of single-cell RNA-seq data using regularized negative binomial regression. Genome Biol..

[49] Pont F, Tosolini M, Fournié JJ (2019). Single-cell signature explorer for comprehensive visualization of single cell signatures across scRNA-seq datasets. Nucl Acids Res.

[50] Liu X (2022). Single-cell RNA sequencing of subcutaneous adipose tissues identifies therapeutic targets for cancer-associated lymphedema. Cell Discov..

[51] Guo X (2018). Global characterization of T cells in non-small-cell lung cancer by single-cell sequencing. Nat. Med..

