# Supplementary material for: Lineage-specific regulatory changes in hypertrophic cardiomyopathy unraveled by single-nucleus RNA-seq and spatial transcriptomics
Source: Cell Discov. 2023 Jan 17;9:6. doi: 10.1038/s41421-022-00490-3 (PMC9842679; doi:10.1038/s41421-022-00490-3)
Supplement: Supplementary file 1 — Supplementary Information [file 41421_2022_490_MOESM1_ESM.pdf]

Supplementary Information

Supplementary figures

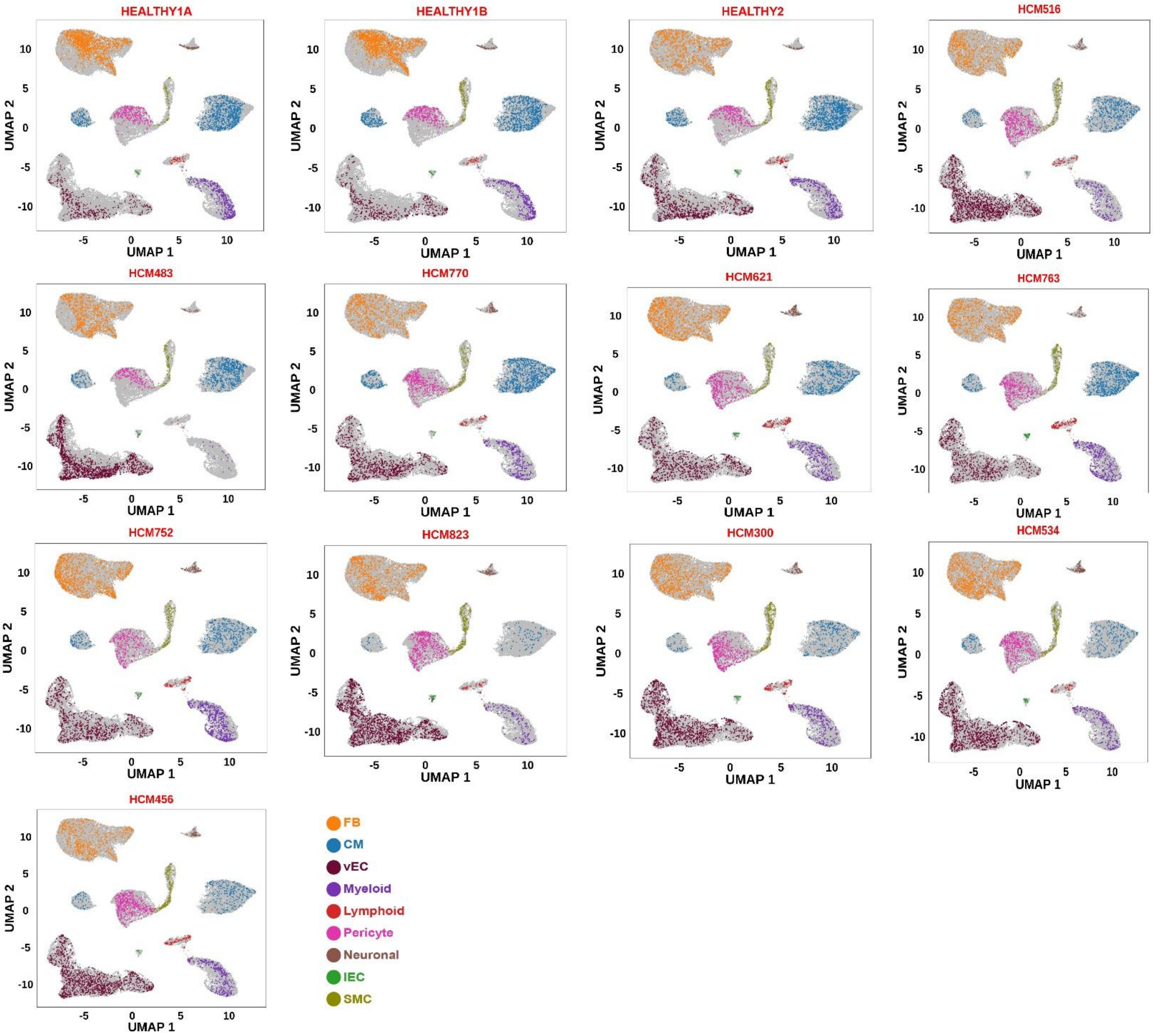

Fig. S1 Distribution of each cell type in the UMAP space for each sample. For comparison, an equal number of cells (3,169) are shown for each sample.

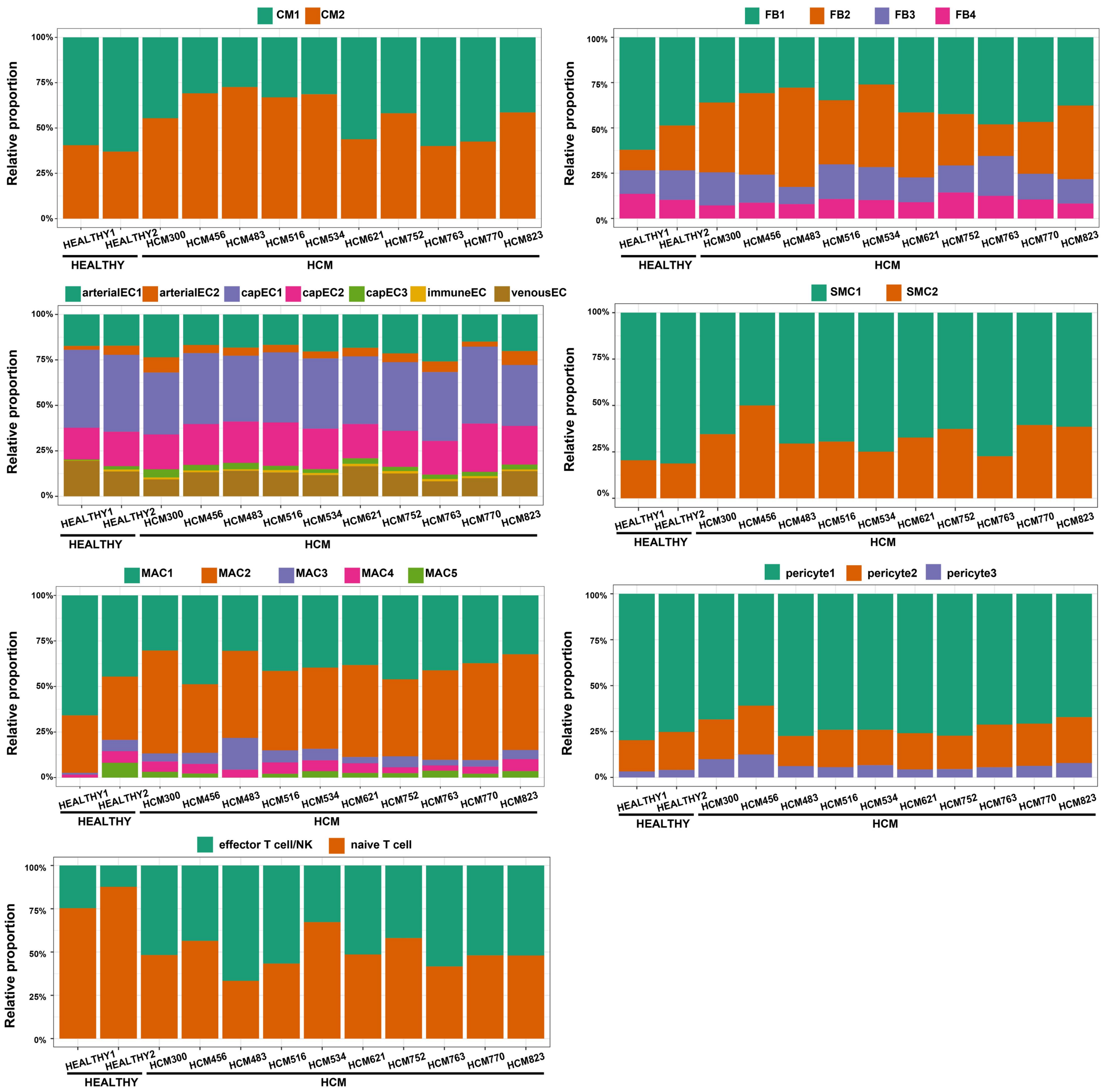

Fig. S2 The relative proportion of each subcluster in each individual for each major cell type.

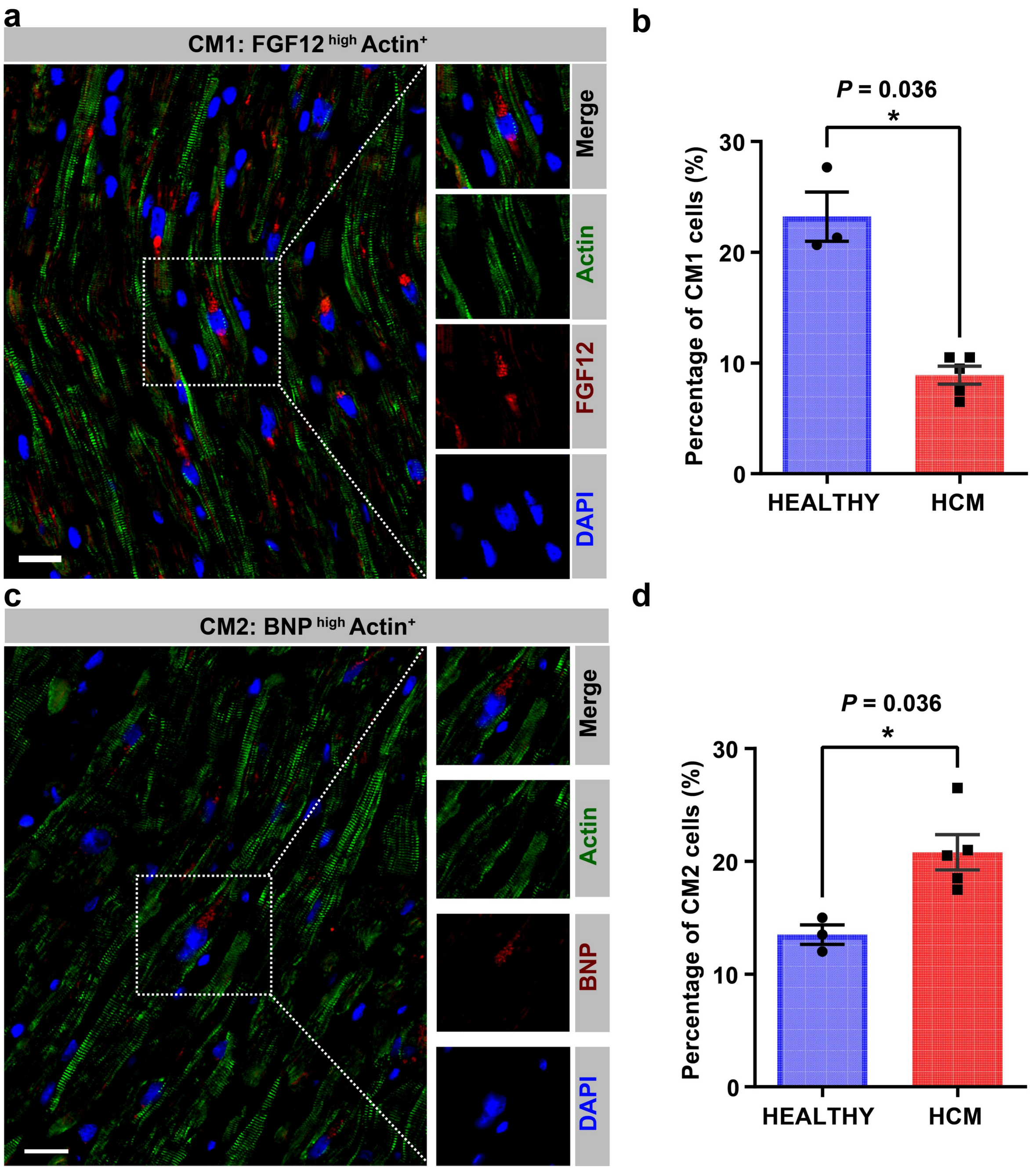

Fig. S3 Immunofluorescence staining confirmed the presence of cardiomyocyte subpopulations in cardiac tissues from healthy donors. a Immunofluorescence staining for CM1 (FGF12 ^{high}). Cardiomyocytes are marked by Actin. **b** Percentage of CM1 cells in all cells based on the staining on cardiac tissue sections from healthy donors (n=3) and HCM patients (n=5). **c** Immunofluorescence staining for CM2 (BNP ^{high}). Cardiomyocytes are marked by Actin. **d** Percentage of CM2 cells in all cells according to the staining on cardiac tissue sections from healthy donors (n=3) and HCM patients (n=5). In **b** and **d**, each value represents the mean percentage of positive cells in five representative fields of view. The data are presented as mean ± standard error of the mean (SEM) *: *P*-value < 0.05, Wilcoxon rank-sum test. Scale bar: 20 μm.

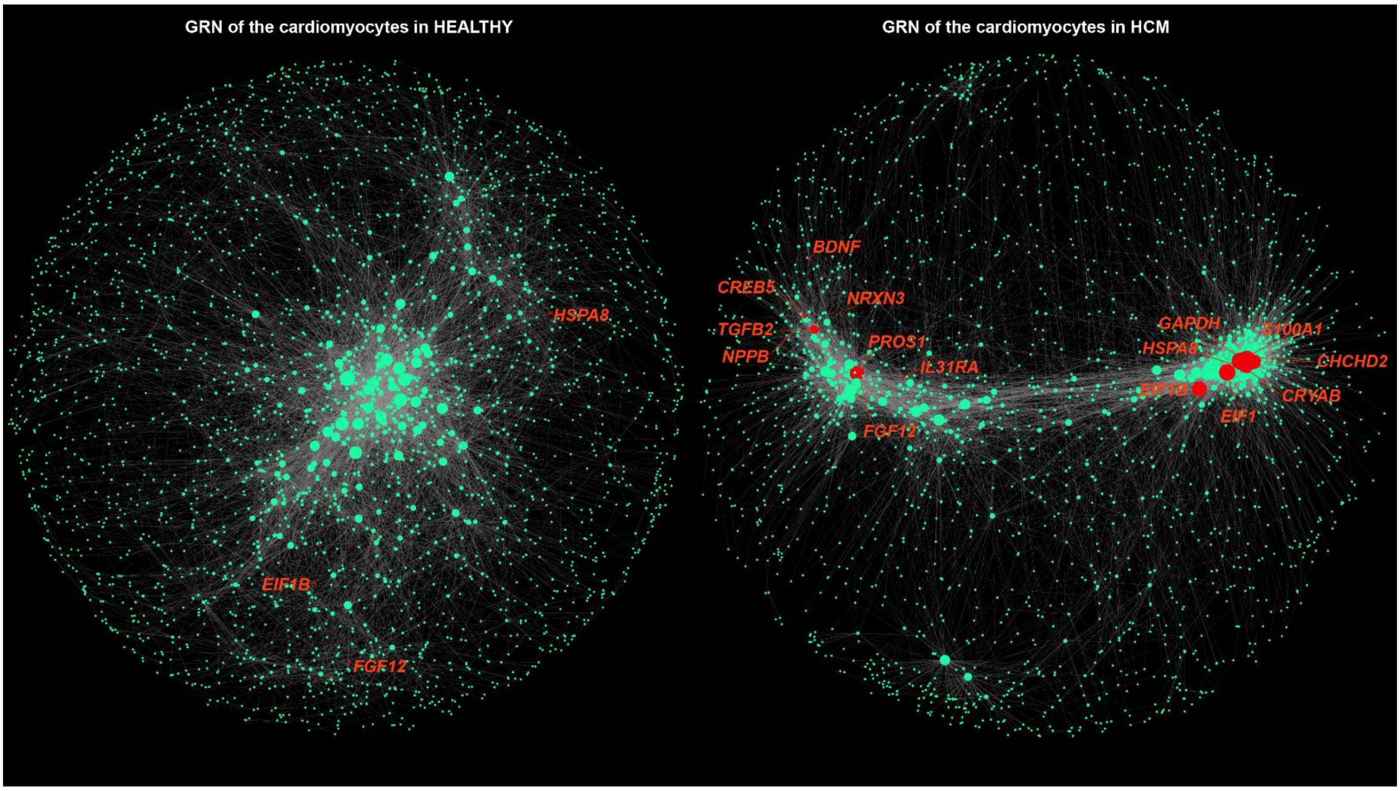

Fig. S4 Comparative analysis of the GRNs of the cardiomyocytes between HEALTHY (left panel) and HCM (right panel). The node size reflects the degree centrality. Nodes in red are representative genes with increased biological importance in the network of HCM compared with that of HEALTHY.

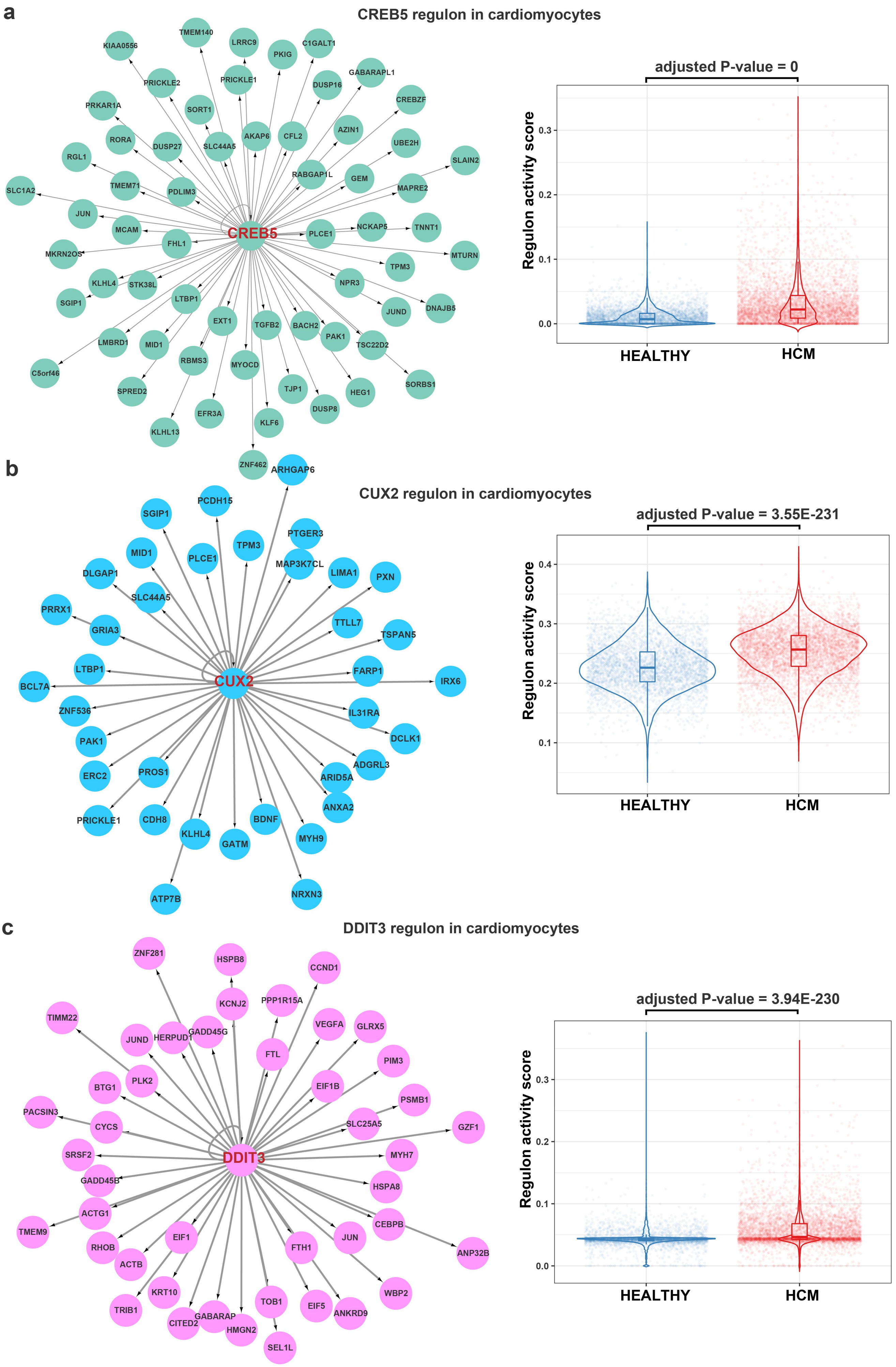
a
CREB5 regulon in cardiomyocytes
CREB5
TMEM140
LRRC9
C1GALT1
KIAA0556
PKIG
PRICKLE1
GABARAPL1
PRICKLE2
DUSP16
PRKAR1A
SORT1
CREBZF
AZIN1
RORA
AKAP6
CFL2
UBE2H
DUSP27
SLC44A5
RGL1
SLAIN2
RABGAP1L
GEM
TMEM71
MAPRE2
SLC1A2
PDLIM3
JUN
NCKAP5
TNNT1
MCAM
PLCE1
FHL1
MKRN2OS
MTURN
TPM3
KLHL4
STK38L
NPR3
SGIP1
LTBP1
JUND
DNAJB5
EXT1
TGFB2
BACH2
LMBRD1
PAK1
MID1
TSC22D2
C5orf46
RBMS3
MYOCD
SORBS1
SPRED2
TJP1
HEG1
DUSP8
EFR3A
KLHL13
KLF6
ZNF462
adjusted P-value = 0
Regulon activity score
0.0
0.1
0.2
0.3
HEALTHY
HCM
b
CUX2 regulon in cardiomyocytes
CUX2
ARHGAP6
PCDH15
SGIP1
PTGER3
TPM3
MID1
PLCE1
MAP3K7CL
LIMA1
DLGAP1
PXN
SLC44A5
PRRX1
TTLL7
GRIA3
TSPAN5
LTBP1
FARP1
BCL7A
IRX6
ZNF536
IL31RA
DCLK1
PAK1
ADGRL3
ERC2
PROS1
ARID5A
ANXA2
PRICKLE1
CDH8
BDNF
KLHL4
GATM
MYH9
ATP7B
NRXN3
adjusted P-value = 3.55E-231
Regulon activity score
0.1
0.2
0.3
0.4
HEALTHY
HCM
c
DDIT3 regulon in cardiomyocytes
DDIT3
ZNF281
HSPB8
CCND1
KCNJ2
PPP1R15A
TIMM22
VEGFA
GLRX5
JUND
HERPUD1
GADD45G
FTL
PIM3
PLK2
EIF1B
BTG1
PSMB1
PACSIN3
CYCS
SLC25A5
GZF1
SRSF2
MYH7
GADD45B
HSPA8
ACTG1
CEBPB
TMEM9
EIF1
JUN
RHOB
FTH1
ANP32B
ACTB
KRT10
WBP2
TRIB1
TOB1
EIF5
GABARAP
ANKRD9
CITED2
HMGN2
SEL1L
adjusted P-value = 3.94E-230
Regulon activity score
0.0
0.1
0.2
0.3
HEALTHY
HCM

Fig. S5 Regulon analysis by using SCENIC confirmed that the regulon activities of three prioritized transcription factors were significantly higher in cardiomyocytes from HCM than HEALTHY. a Network plot showing *CREB5* and its predicted targets (left panel) and violin plot showing the distributions of the regulon activity score in HCM and HEALTHY (right panel). **b** Network plot showing *CUX2* and its predicted targets (left panel) and violin plot showing the distributions of the regulon activity score in HCM and HEALTHY (right panel). **c** Network plot showing *DDIT3* and its predicted targets (left panel) and violin plot showing the distributions of the regulon activity score in HCM and HEALTHY (right panel). For regulon activity comparison, the statistical significance threshold was set to a Bonferroni-adjusted *P*-value of Wilcoxon rank-sum test < 0.05.

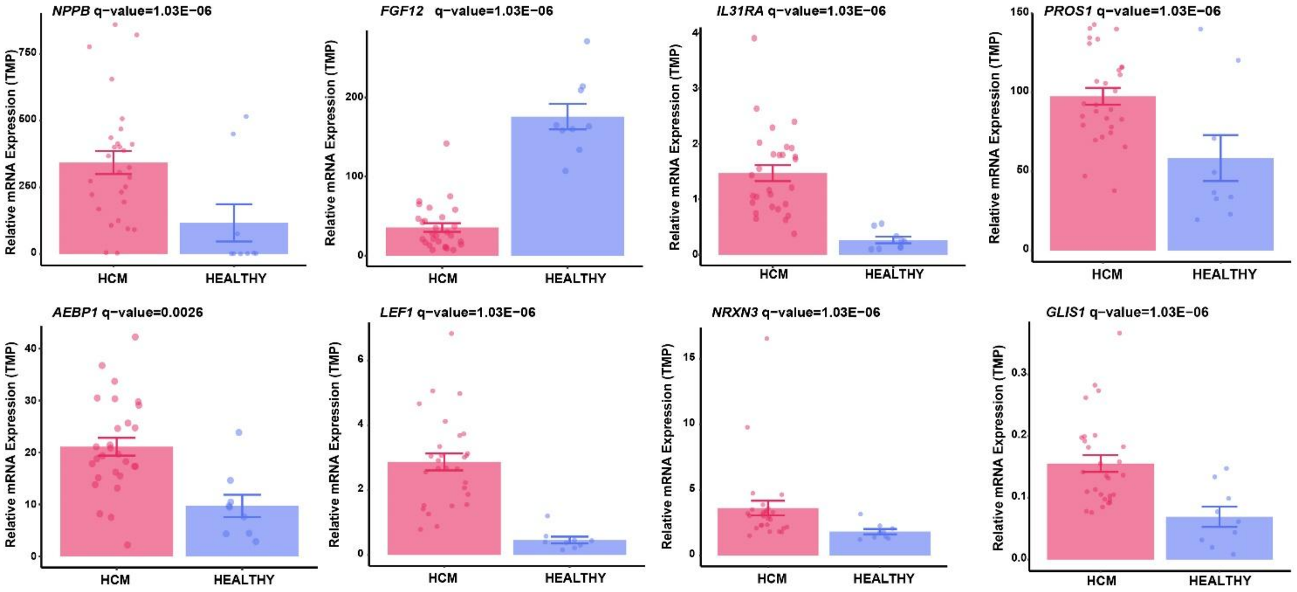

Fig. S6 Relative mRNA expression of representative candidate target/marker genes in HCM (n=28) and HEALTHY (n=9) determined by bulk RNA-seq.

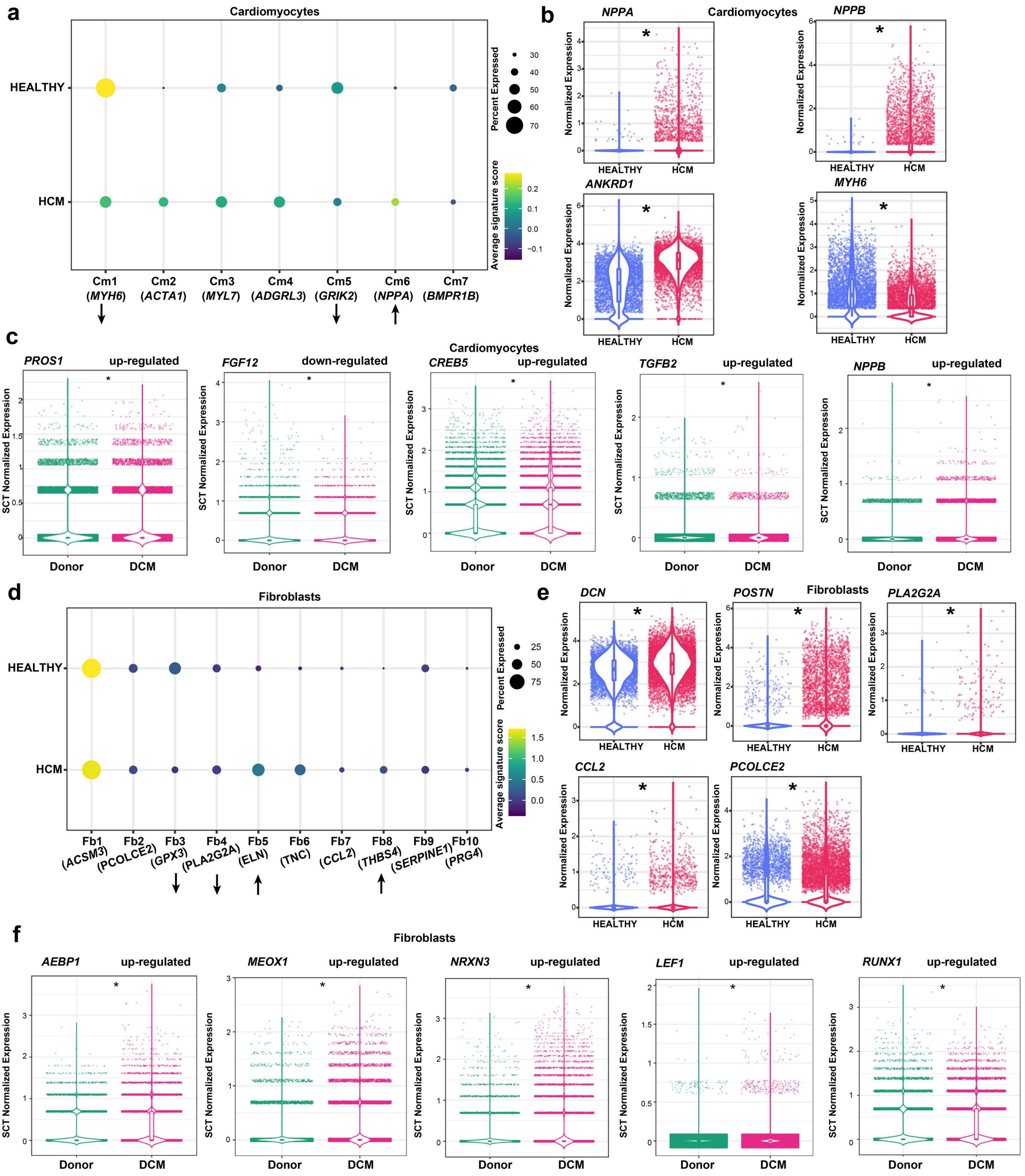

Fig. S7 Comparative analysis of the snRNA-seq dataset generated by Koenig et al. (2022, Nature Cardiovascular Research) on dilated cardiomyopathy (DCM) and ours on HCM with an emphasis on cardiomyocytes and fibroblasts. a Dotplot showing the average expression of the molecular signatures of the cardiomyocyte subclusters (Cm1-Cm7) reported by Koenig et al. in our dataset (cardiomyocytes from healthy control and HCM patients). ↑: expanded subpopulations reported in DCM. ↓: contracted subpopulation reported in DCM. The "AddModuleScore" function of Seurat was used to score the molecular signature of each subcluster in each nucleus. The molecular signatures reported by Koenig et al. are as follows: Cm1 (*MYH6, FGF12, TMEM178B*), Cm2 (*ACTA1, ANKRD1, FLNC, NRAP, XIRP2*), Cm3 (*MYL7, TPM2, NMRK2, MYL4*), Cm4 (*ADGRL3, MID1, EDIL3, PLCE1*), Cm5 (*GRIK2, BRINP3, HS6ST3*), Cm6

(*NPPA, NPPB*), and Cm7 (*BMPR1B, COL25A1, PDE1A, SLIT2, ANKRD45*). **b** The expression of the representative dysregulated genes in DCM cardiomyocytes (shown in Fig.3f of Koenig et al.'s paper) in our dataset. *: adjusted *P*-value < 0.05. **c** The expression of the potential key genes during the transition towards a failing state of cardiomyocytes we prioritized in Koenig et al.'s dataset (cardiomyocytes from healthy donors and DCM). *: P-value < 0.05, Wilcoxon rank-sum test. **d** Dotplot showing the average expression of the molecular signatures of the fibroblast subclusters (Fb1-Fb10) reported by Koenig et al. in our dataset (fibroblasts from healthy control and HCM patients).). ↑: expanded subpopulations reported in DCM. ↓: contracted subpopulation reported in DCM. The molecular signatures reported by Andrew et al. are as follows: Fb1 (*ACSM3, SCN7A, ABCA10, NEGR1, ABCA9*), Fb2 (*PCOLCE2, IGFBP6, MFAP5, S100A10, FGFBP2*), Fb3 (*GPX3, APOD, C3, HSPA1A, GLUL*), Fb4 (*PLA2G2A, MID1, RARRES1, IGFBP4, FGF7*), Fb5 (*ELN, GPC6, FGF14, ITGA1*), Fb6 (*TNC, FN1, MEOX1*), Fb7 (*CCL2, THBS1, CYR61, NR4A1*), Fb8 (*THBS4, AEBP1, POSTN, CLU, COMP*), Fb9 (*SERPINE1, CYR61, NFATC2, LRRFIP1*), and Fb10 (*PRG4, SLC39A3, NAMPT, PKHD1L1*). **e** The expression of the representative dysregulated genes in DCM fibroblasts (shown in Fig.6i of Koenig et al.'s paper) in our dataset. *: adjusted P-value < 0.05. **f** The expression of the potential key genes in cardiac fibrosis we prioritized in Koenig et al.'s dataset (fibroblasts from healthy donors and DCM). *: *P*-value < 0.05, Wilcoxon rank-sum test.

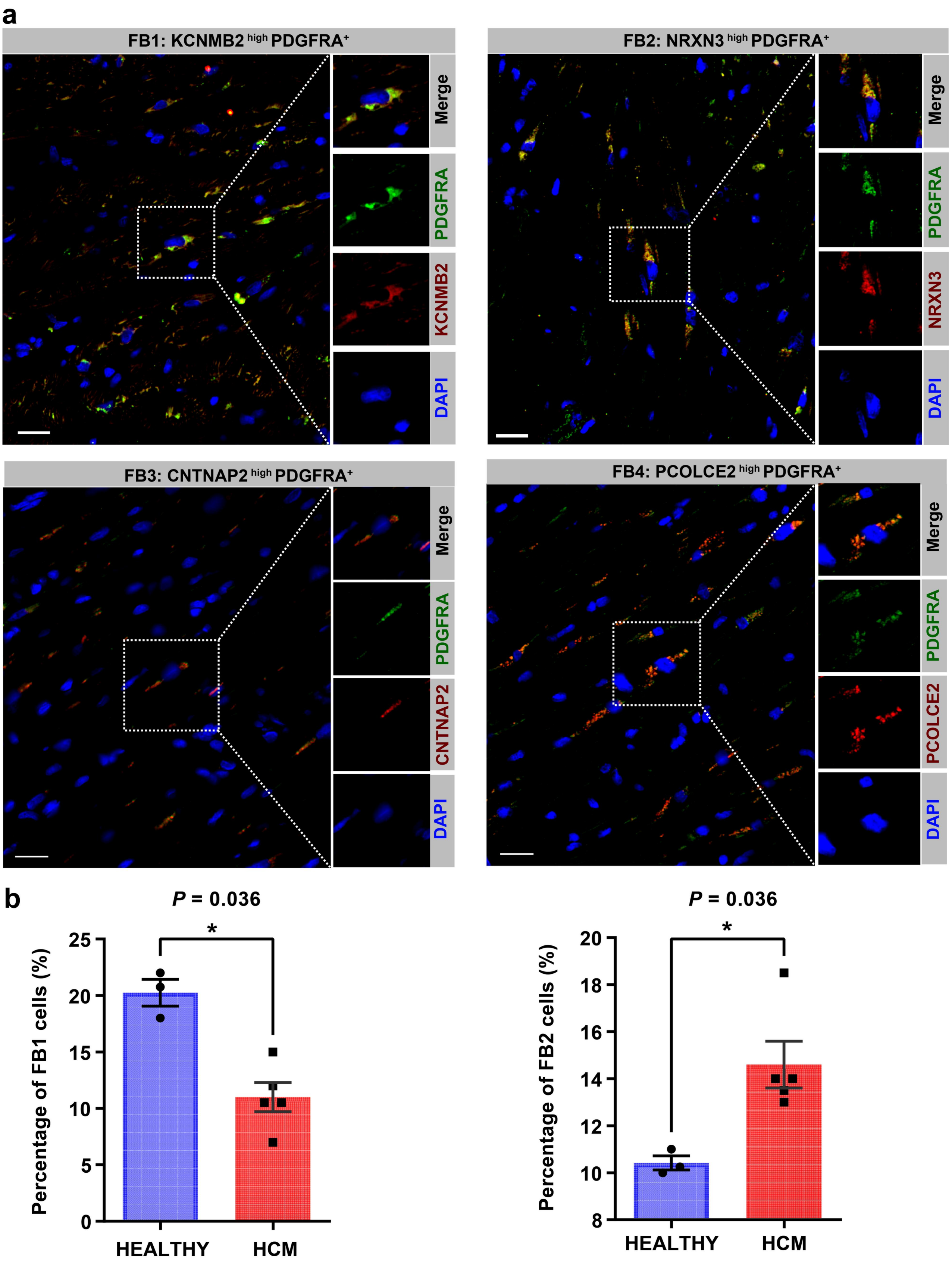

Fig. S8 Immunofluorescence staining confirmed the presence of fibroblast subpopulations in cardiac tissues from healthy donors. a Immunofluorescence staining for the four subpopulations of fibroblasts. Fibroblasts are marked by PDGFRA. **b** Percentage of FB1 (left) or FB2 (right) cells in all cells according to the staining on the cardiac tissue sections from healthy donors (n=3) and HCM patients (n=5). Each value represents the mean percentage of positive cells in five representative fields of view. The data are presented as mean ± standard error of the mean (SEM) *: *P*-value < 0.05, Wilcoxon rank-sum test. Scale bar: 20 μm.

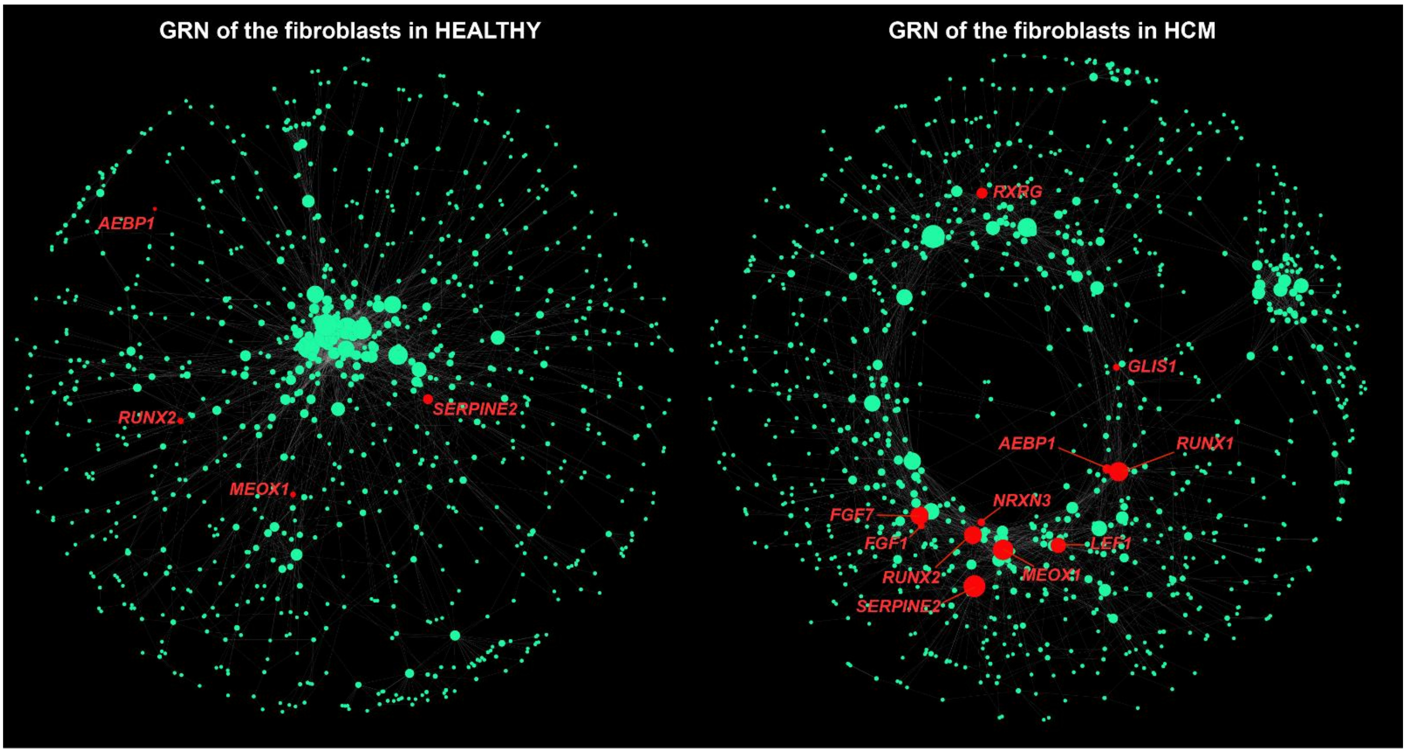

Fig. S9 Comparative analysis of the GRNs of the fibroblasts between HEALTHY (left panel) and HCM (right panel). The node size reflects the degree centrality. Nodes in red are representative genes with increased biological importance in the network of HCM compared with that of HEALTHY.

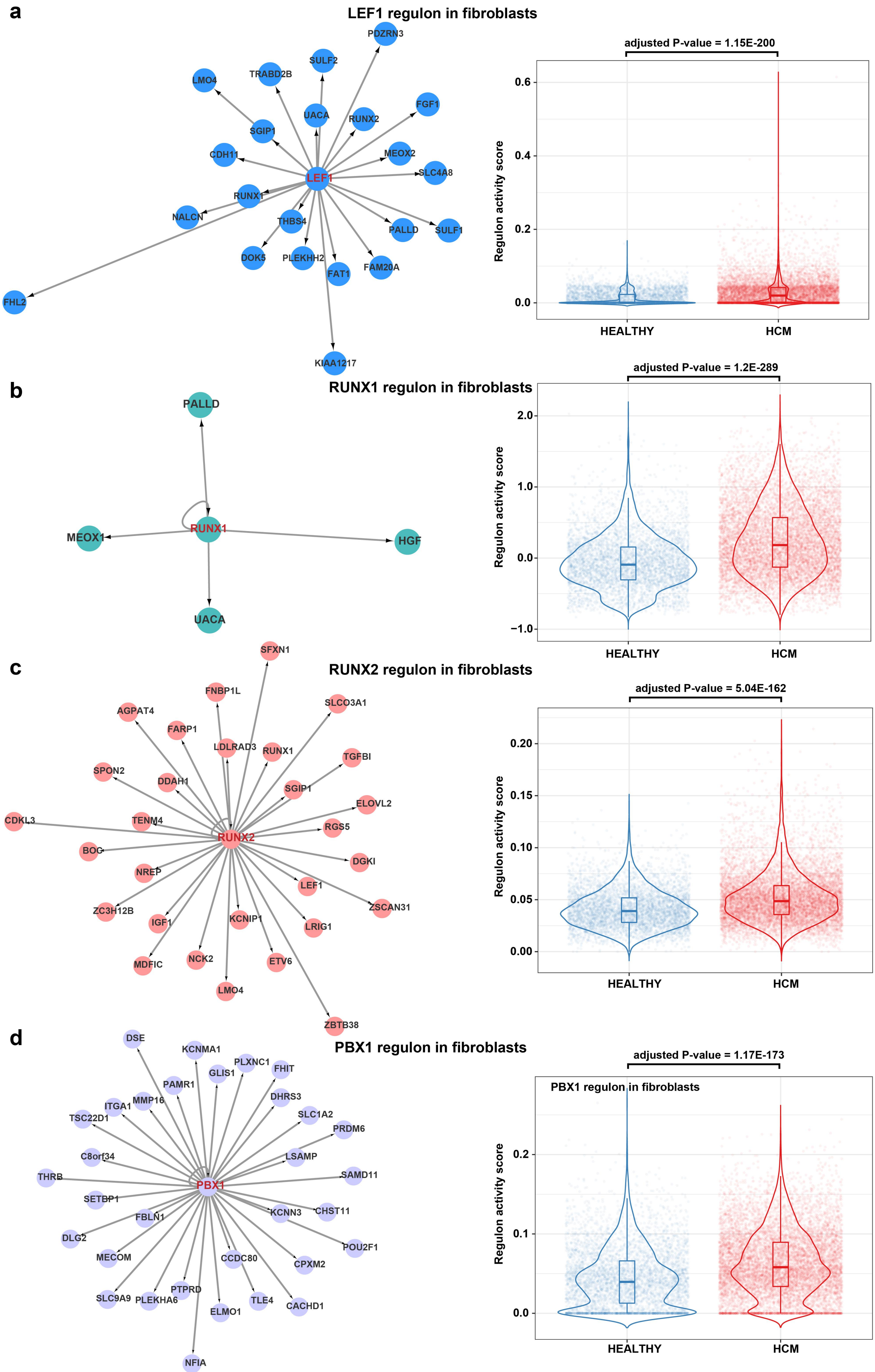
a
LEF1 regulon in fibroblasts
PDZRN3
SULF2
TRABD2B
LMO4
UACA
FGF1
RUNX2
SGIP1
CDH11
MEOX2
SLC4A8
LEF1
RUNX1
NALCN
THBS4
PALLD
SULF1
DOK5
PLEKHH2
FAT1
FAM20A
FHL2
KIAA1217
adjusted P-value = 1.15E-200
Regulon activity score
0.6
0.4
0.2
0.0
HEALTHY
HCM
b
RUNX1 regulon in fibroblasts
PALLD
MEOX1
RUNX1
HGF
UACA
adjusted P-value = 1.2E-289
Regulon activity score
2.0
1.0
0.0
−1.0
HEALTHY
HCM
c
RUNX2 regulon in fibroblasts
SFXN1
FNBP1L
SLCO3A1
AGPAT4
FARP1
LDLRAD3
RUNX1
TGFBI
SPON2
DDAH1
SGIP1
ELOVL2
CDKL3
TENM4
RGS5
RUNX2
BOC
DGKI
NREP
LEF1
ZSCAN31
ZC3H12B
IGF1
KCNIP1
LRIG1
MDFIC
NCK2
ETV6
LMO4
ZBTB38
adjusted P-value = 5.04E-162
Regulon activity score
0.20
0.15
0.10
0.05
0.00
HEALTHY
HCM
d
PBX1 regulon in fibroblasts
DSE
KCNMA1
PLXNC1
FHIT
GLIS1
PAMR1
DHRS3
ITGA1
MMP16
SLC1A2
TSC22D1
PRDM6
LSAMP
C8orf34
SAMD11
THRB
PBX1
SETBP1
CHST11
KCNN3
FBLN1
DLG2
POU2F1
MECOM
CCDC80
CPXM2
PTPRD
SLC9A9
PLEKHA6
TLE4
CACHD1
ELMO1
NFIA
adjusted P-value = 1.17E-173
PBX1 regulon in fibroblasts
Regulon activity score
0.2
0.1
0.0
HEALTHY
HCM

Fig. S10 Regulon analysis by using SCENIC confirmed that the regulon activities of four prioritized transcription factors were significantly higher in fibroblasts from HCM than HEALTHY. a Network plot showing LEF1 and its predicted targets (left panel) and violin plot showing the distributions of the regulon activity score in HCM and HEALTHY (right panel). **b** Network plot showing *RUNX1* and its predicted targets (left panel) and violin plot showing the distributions of the regulon activity score in HCM and HEALTHY (right panel). **c** Network plot showing *RUNX2* and its predicted targets (left panel) and violin plot showing the distributions of the regulon activity score in HCM and HEALTHY (right panel). **d** Network plot showing *PBX1* and its predicted targets (left panel) and violin plot showing the distributions of the regulon activity score in HCM and HEALTHY (right panel). For regulon activity comparison, the statistical significance threshold was set to a Bonferroni-adjusted *P*-value of Wilcoxon rank-sum test < 0.05.

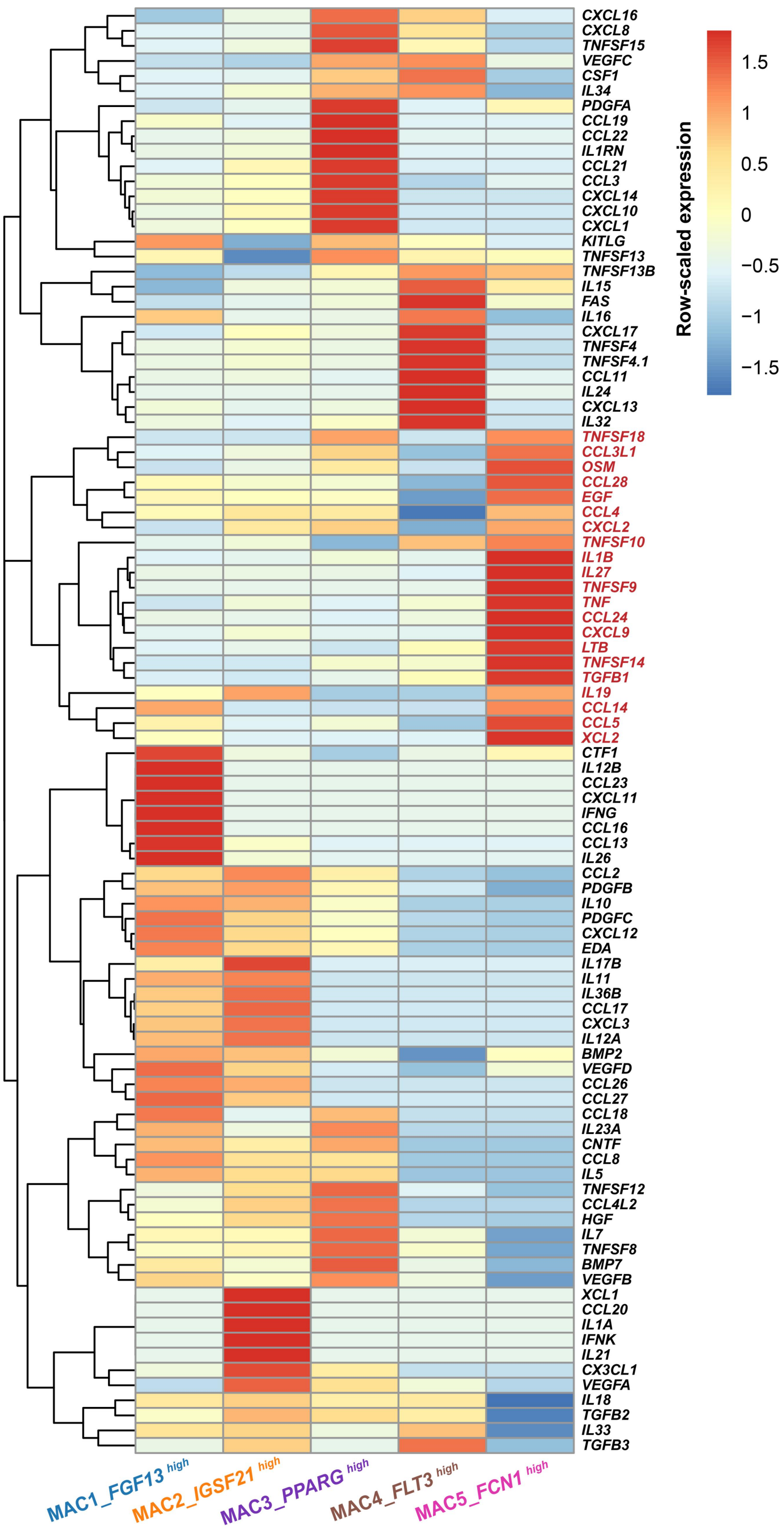

Fig. S11 Heatmap showing the expression profile of the cytokines expressed across the five macrophage subclusters.

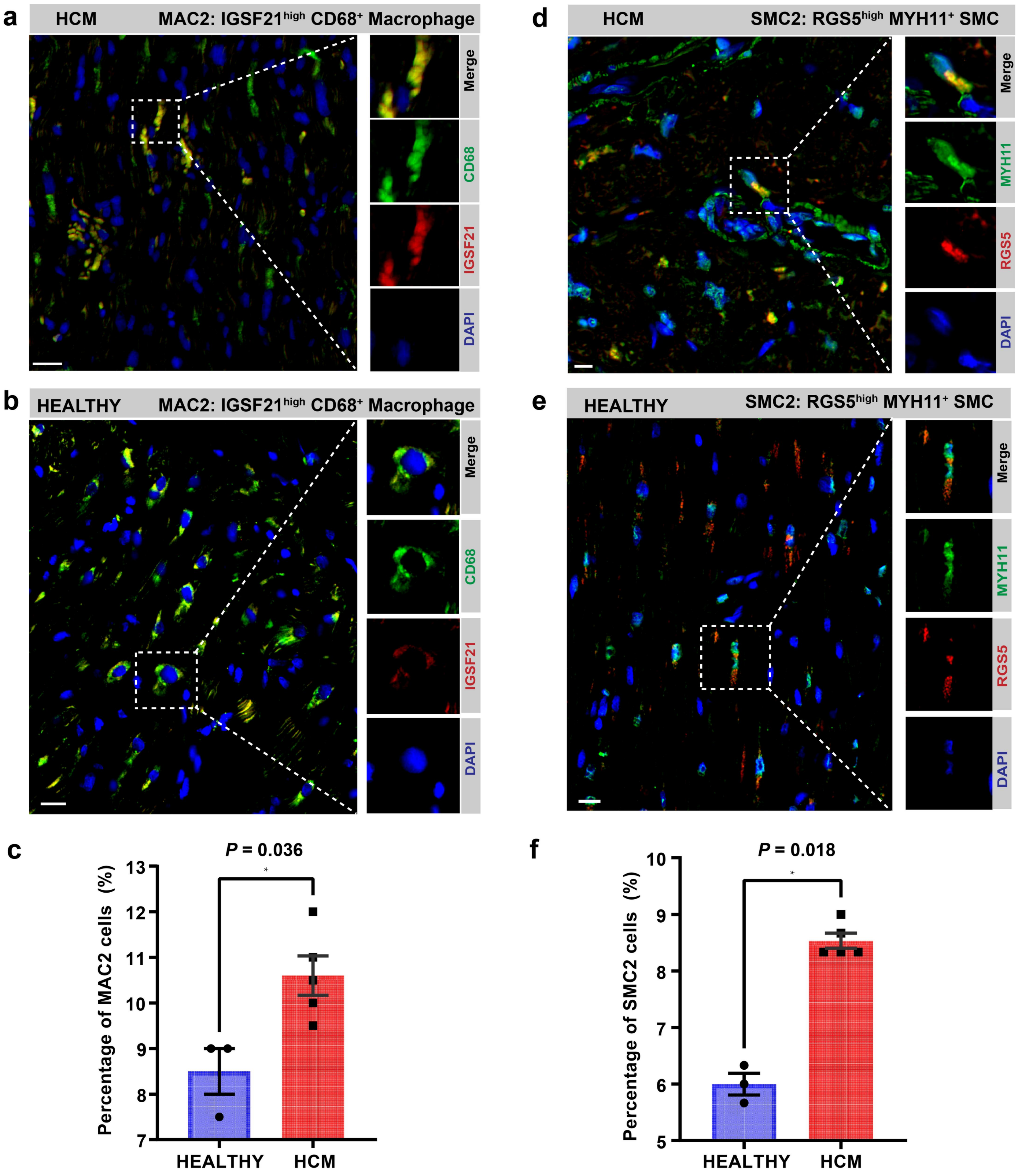

Fig. S12 Immunofluorescence staining confirmed the presence of the subpopulations MAC2 and SMC2 in cardiac tissues from HCM patients or healthy donors. a Immunofluorescence staining for MAC2 ($IGSF21^{high}$) in cardiac tissues from HCM patients. Macrophages are marked by CD68. **b** Immunofluorescence staining for MAC2 in cardiac tissues from healthy donors. **c** Percentage of MAC2 cells in all cells according to the staining on cardiac tissue sections from healthy donors (n=3) or HCM patients (n=5). **d** Immunofluorescence staining for SMC2 ($RGS5^{high}$) in cardiac tissues from HCM patients. SMCs are marked by MYH11. **e** Immunofluorescence staining for SMC2 in cardiac tissues from healthy donors. **f** Percentage of SMC2 cells in all cells according to the staining on cardiac tissue sections from healthy donors (n=3) or HCM patients (n=5). In c and f, each value represents the mean percentage of positive cells

in five representative fields of view. The data are presented as mean ± standard error of the mean (SEM) *: *P*-value < 0.05, Wilcoxon rank-sum test. Scale bar: 10 μm.

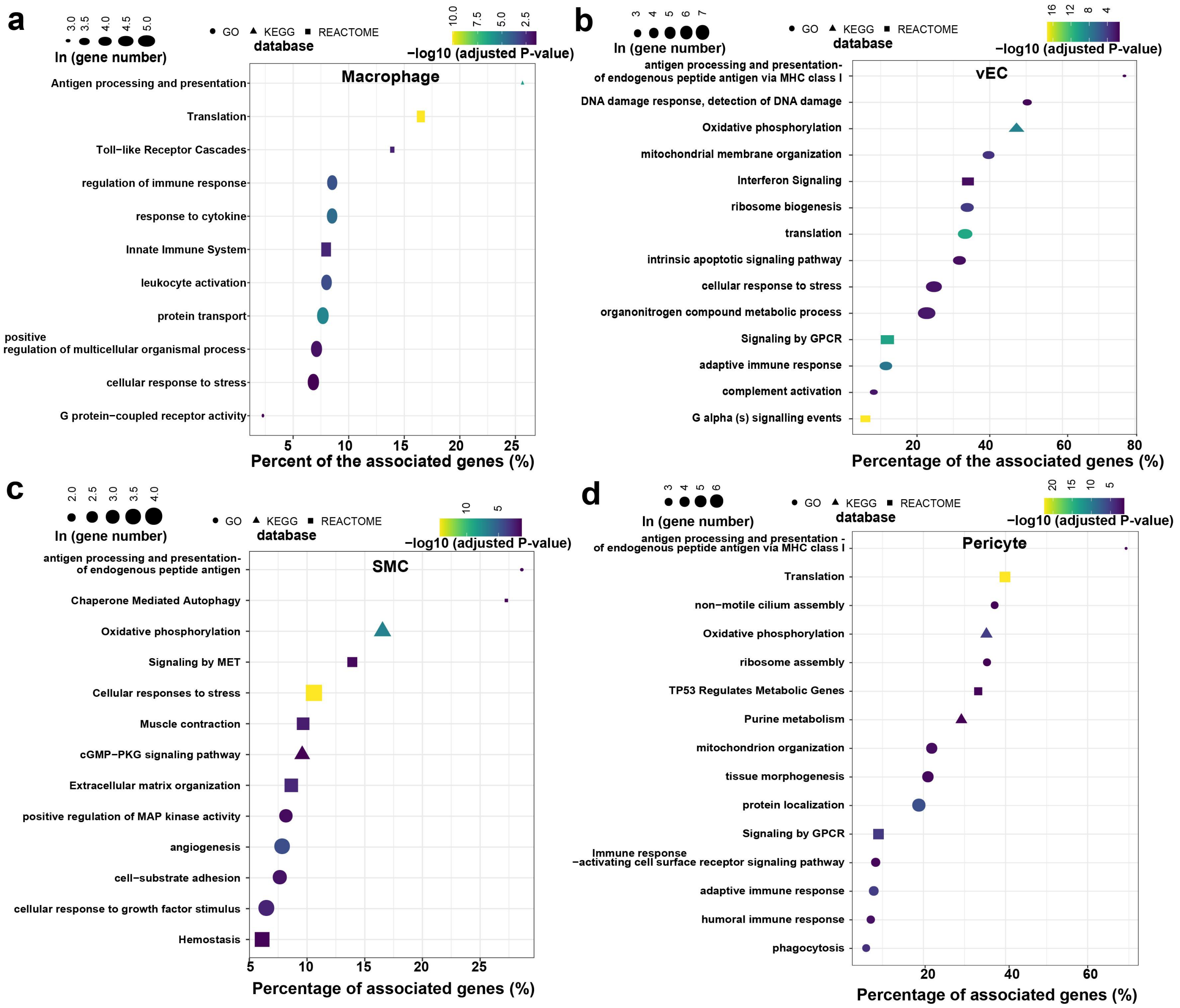

Fig. S13 Functional enrichment of the upregulated genes in macrophages (a), vascular endothelial cells (b), smooth muscle cells (c), and pericytes (d).

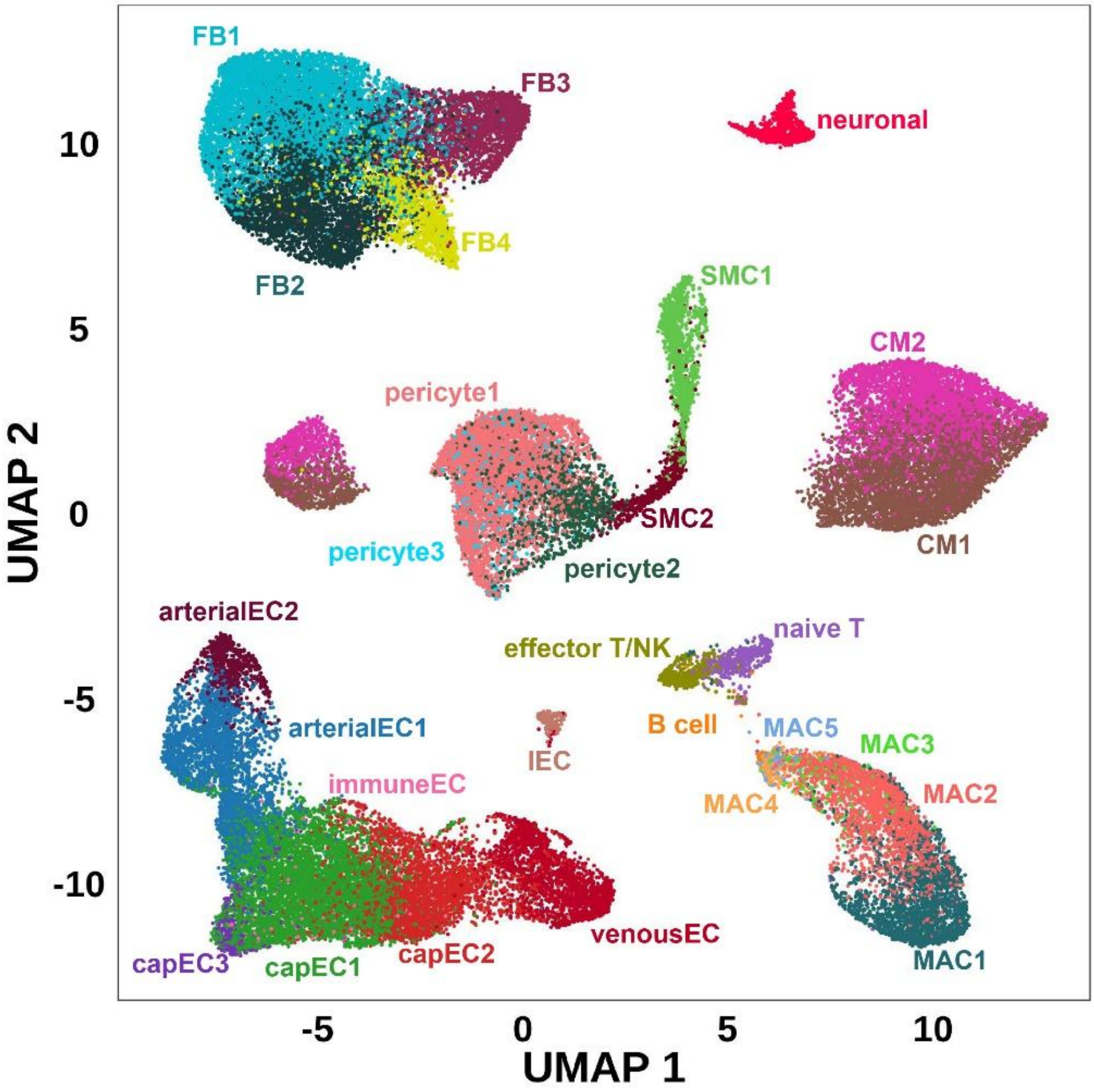

Fig. S14 The distribution of each subpopulation in the UMAP space.

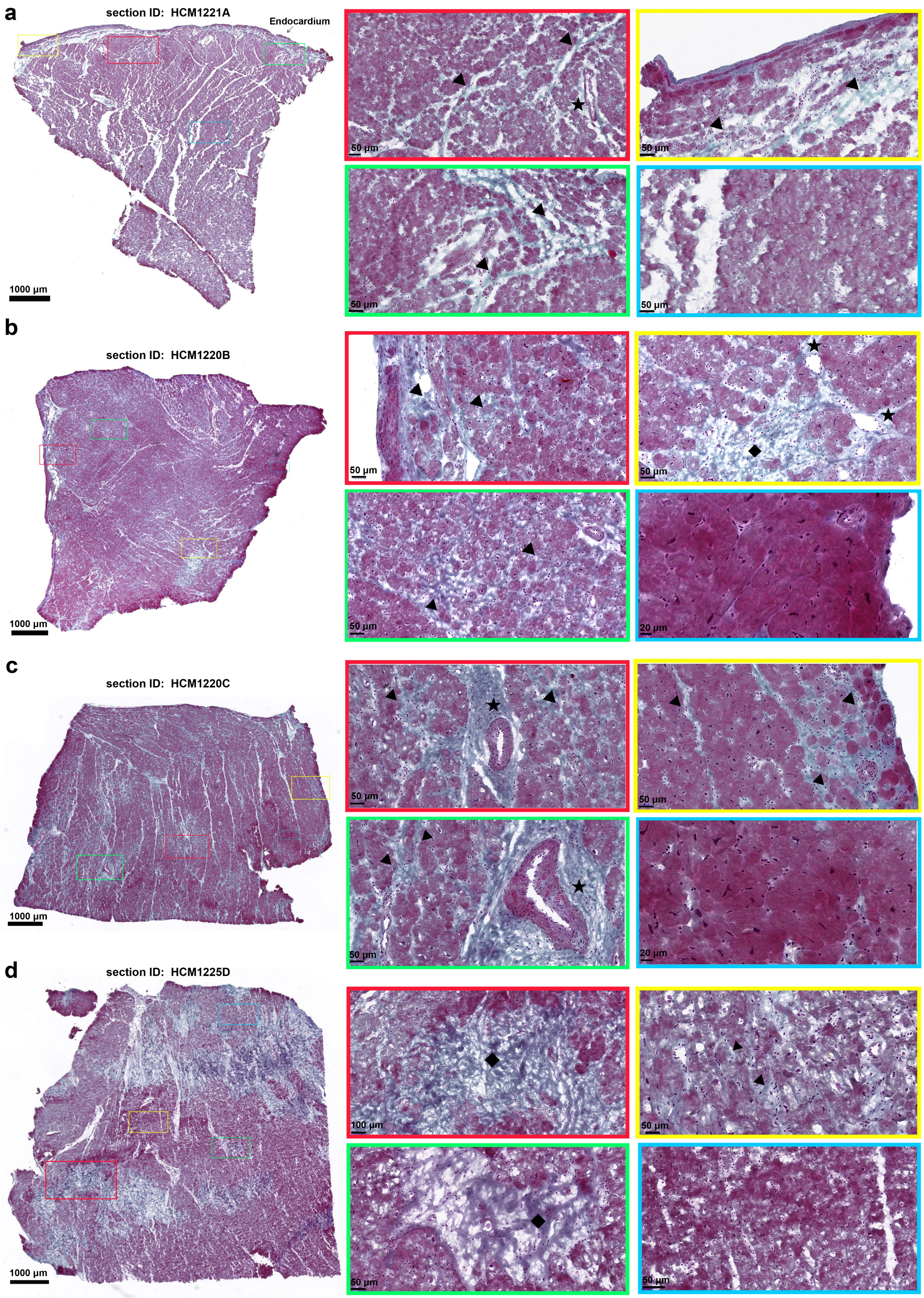

Fig. S15 Masson's trichrome staining showing fibrosis on the cardiac sections adjacent to the four sections (HCM1221A, HCM1220B, HCM1220C, and HCM1225D) selected for spatial transcriptomic

assays. a Masson's trichrome staining for the section adjacent to HCM1221A. **b** Masson's trichrome staining for the section adjacent to HCM1220B. **c** Masson's trichrome staining for the section adjacent to HCM1220C. **d** Masson's trichrome staining for the section adjacent to HCM1225D. Fibrotic regions are indicated by collagens stained in blue. ◆: replacement fibrotic scar; ★ perivascular fibrosis; ▲: diffuse interstitial fibrosis including fibrous bands surrounding the cardiac muscle bundles or individual cardiomyocytes. The small panels with cyan borders show cells in non-fibrotic regions.

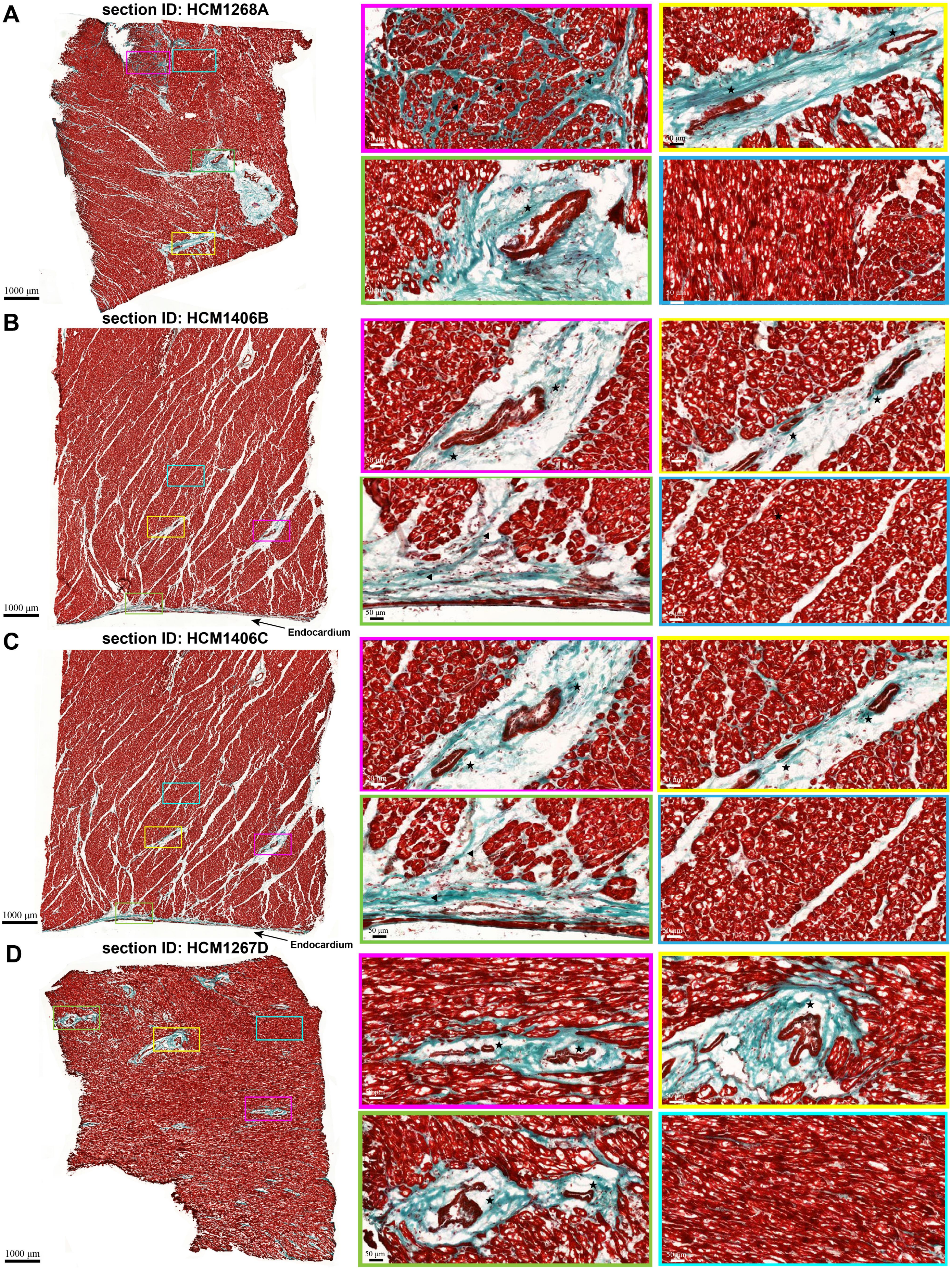

Fig. S16 Masson's trichrome staining showing fibrosis on the cardiac sections adjacent to the four sections (HCM1268A, HCM1406B, HCM1406C, and HCM1267D) selected for spatial transcriptomic assays. a Masson's trichrome staining for the section adjacent to HCM1268A. **b** Masson's trichrome

staining for the section adjacent to HCM1406B. **c** Masson's trichrome staining for the section adjacent to HCM1406C. **d** Masson's trichrome staining for the section adjacent to HCM1267D. Fibrotic regions are indicated by collagens stained in blue. ★ perivascular fibrosis; ▲: diffuse interstitial fibrosis including fibrous bands surrounding the cardiac muscle bundles or individual cardiomyocytes. The small panels with cyan borders show cells in non-fibrotic regions.

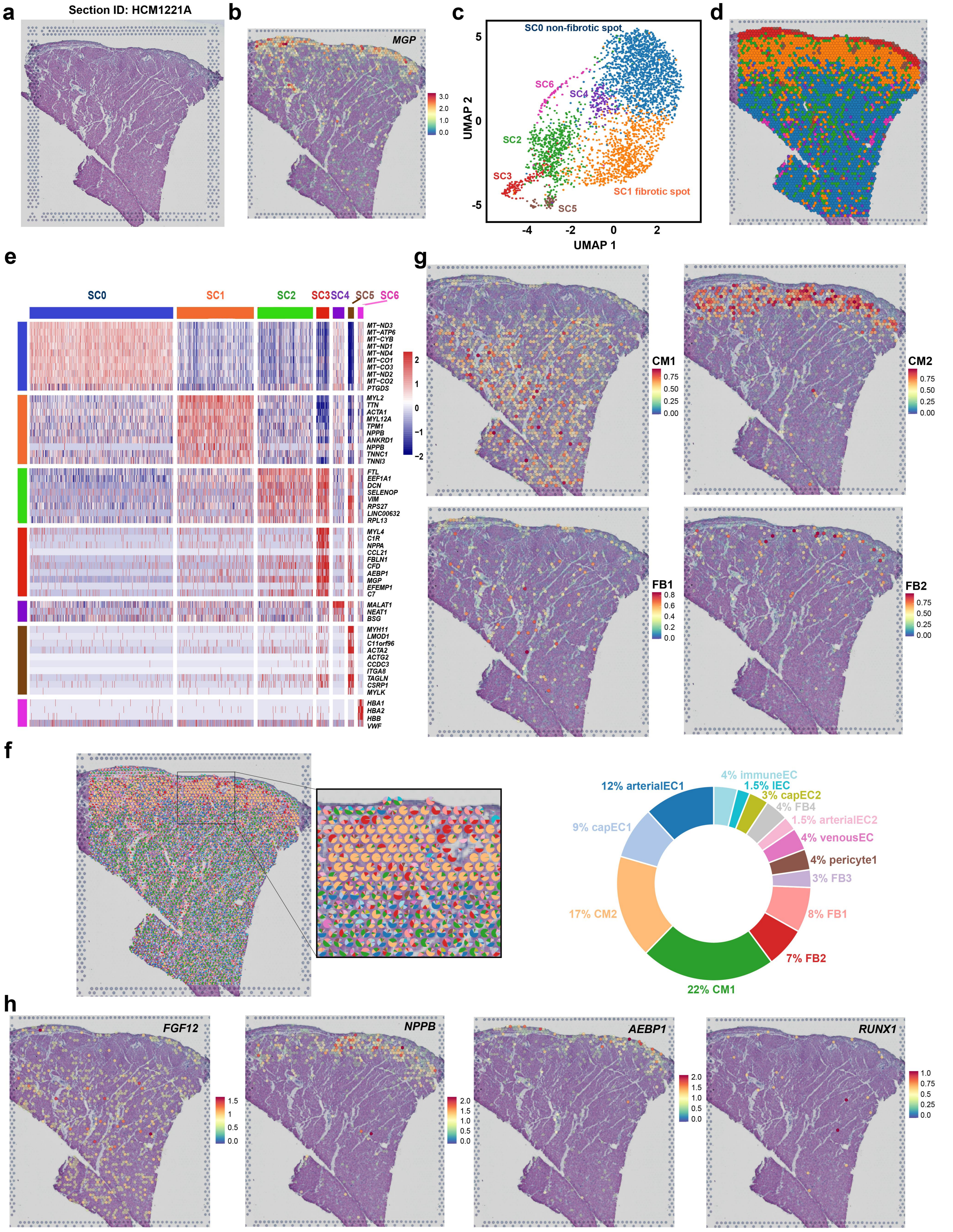

Fig. S17 Spatial transcriptomic analysis of the cardiac tissue section HCM1221A. **a** H&E staining image. **b** Expression distribution of the fibrosis marker MGP. **c** Clustering of the spots. Spot clusters that correspond to fibrotic and non-fibrotic regions are labeled. **d** Spots color-coded by spot clusters. **e** Heatmap

showing the expression signature of each spot cluster. **f** Spot-level cellular subpopulation composition. The pie chart on the right shows the overall composition of the section. Potential noise labels were filtered based on quantile (threshold=0.5). The integration of snRNA-seq and ST data was performed by following the label transfer workflow of Seurat. **g** Probabilistic classification of each spot for the cellular subpopulation CM1, CM2, FB1, and FB2. **h** Expression distribution of representative markers and candidate genes on the section.

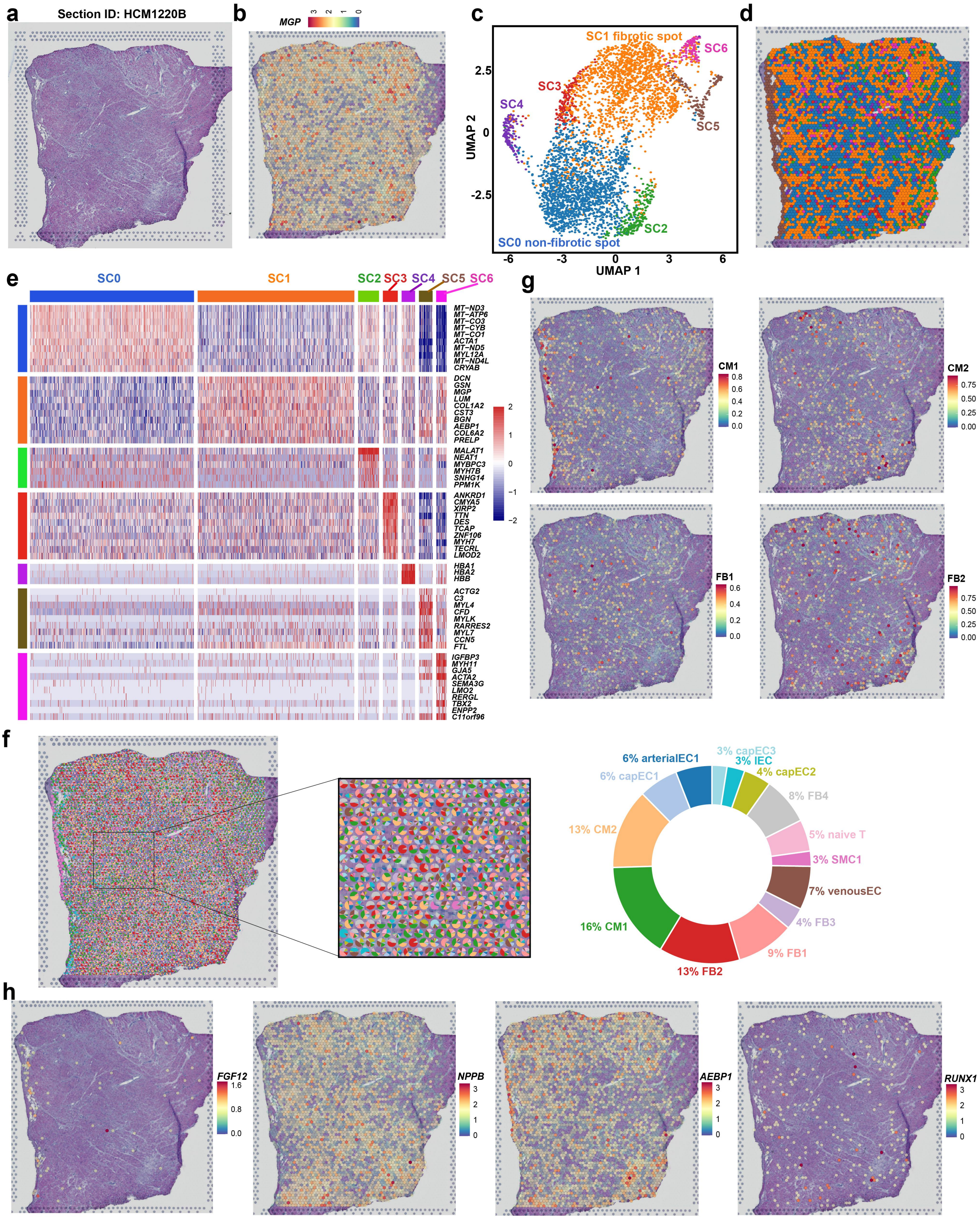

Fig. S18 Spatial transcriptomic analysis of the cardiac tissue section HCM1220B. a H&E staining image. **b** Expression distribution of the fibrosis marker *MGP*. **c** Clustering of the spots. Spot clusters that correspond to fibrotic and non-fibrotic regions are labeled. **d** Spots color-coded by spot clusters. **e** Heatmap showing the expression signature of each spot cluster. **f** Spot-level cellular subpopulation composition. The pie chart on the right shows the overall composition of the section. Potential noise labels were filtered based

on quantile (threshold=0.5). The integration of snRNA-seq and ST data was performed by following the label transfer workflow of Seurat. **g** Probabilistic classification of each spot for the cellular subpopulation CM1, CM2, FB1, and FB2. **h** Expression distribution of representative markers and candidate genes on the section.

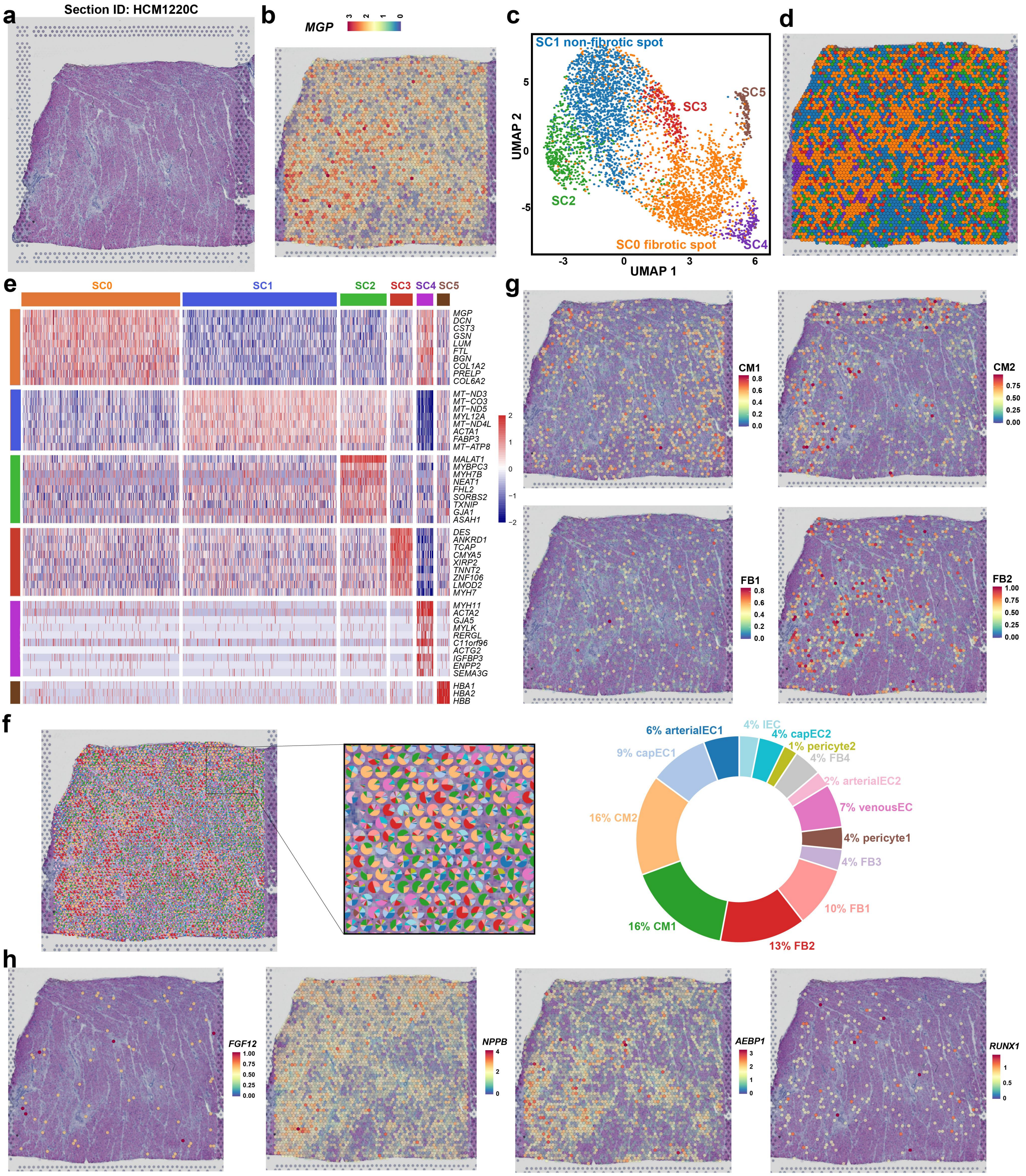

Fig. S19 Spatial transcriptomic analysis of the cardiac tissue section HCM1220C. **a** H&E staining image. **b** Expression distribution of the fibrosis marker MGP. **c** Clustering of the spots. Spot clusters that correspond to fibrotic and non-fibrotic regions are labeled. **d** Spots color-coded by spot clusters. **e** Heatmap showing the expression signature of each spot cluster. **f** Spot-level cellular subpopulation composition. The

pie chart on the right shows the overall composition of the section. Potential noise labels were filtered based on quantile (threshold=0.5). The integration of snRNA-seq and ST data was performed by following the label transfer workflow of Seurat. **g** Probabilistic classification of each spot for the cellular subpopulation CM1, CM2, FB1, and FB2. **h** Expression distribution of representative markers and candidate genes on the section.

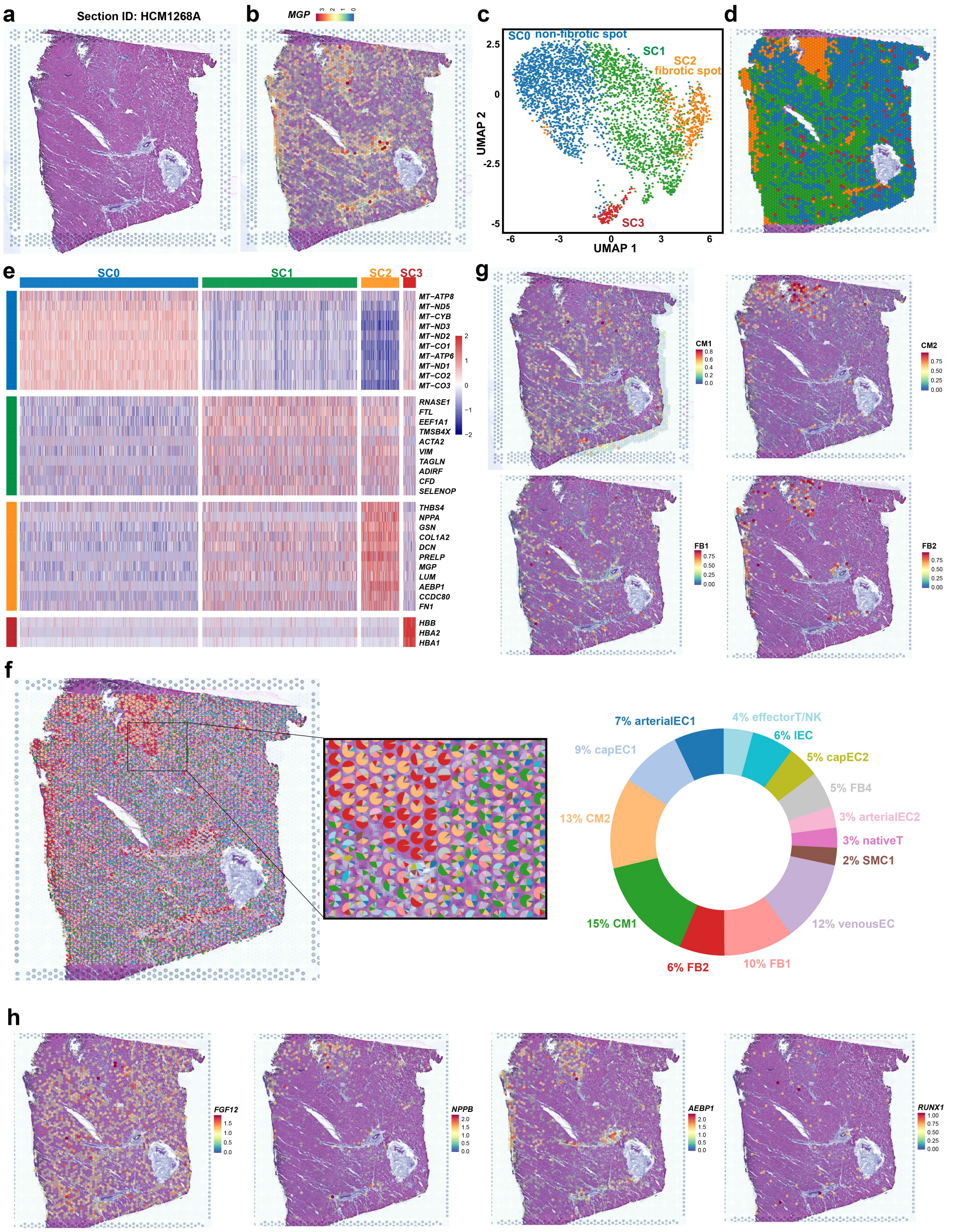

Fig. S20 Spatial transcriptomic analysis of the cardiac tissue section HCM1268A. **a** H&E staining image. **b** Expression distribution of the fibrosis marker *MGP*. **c** Clustering of the spots. Spot clusters that correspond to fibrotic and non-fibrotic regions are labeled. **d** Spots color-coded by spot clusters. **e** Heatmap showing the expression signature of each spot cluster. **f** Spot-level cellular subpopulation composition. The

pie chart on the right shows the overall composition of the section. Potential noise labels were filtered based on quantile (threshold=0.5). The integration of snRNA-seq and ST data was performed by following the label transfer workflow of Seurat. **g** Probabilistic classification of each spot for the cellular subpopulation CM1, CM2, FB1, and FB2. **h** Expression distribution of representative markers and candidate genes on the section.

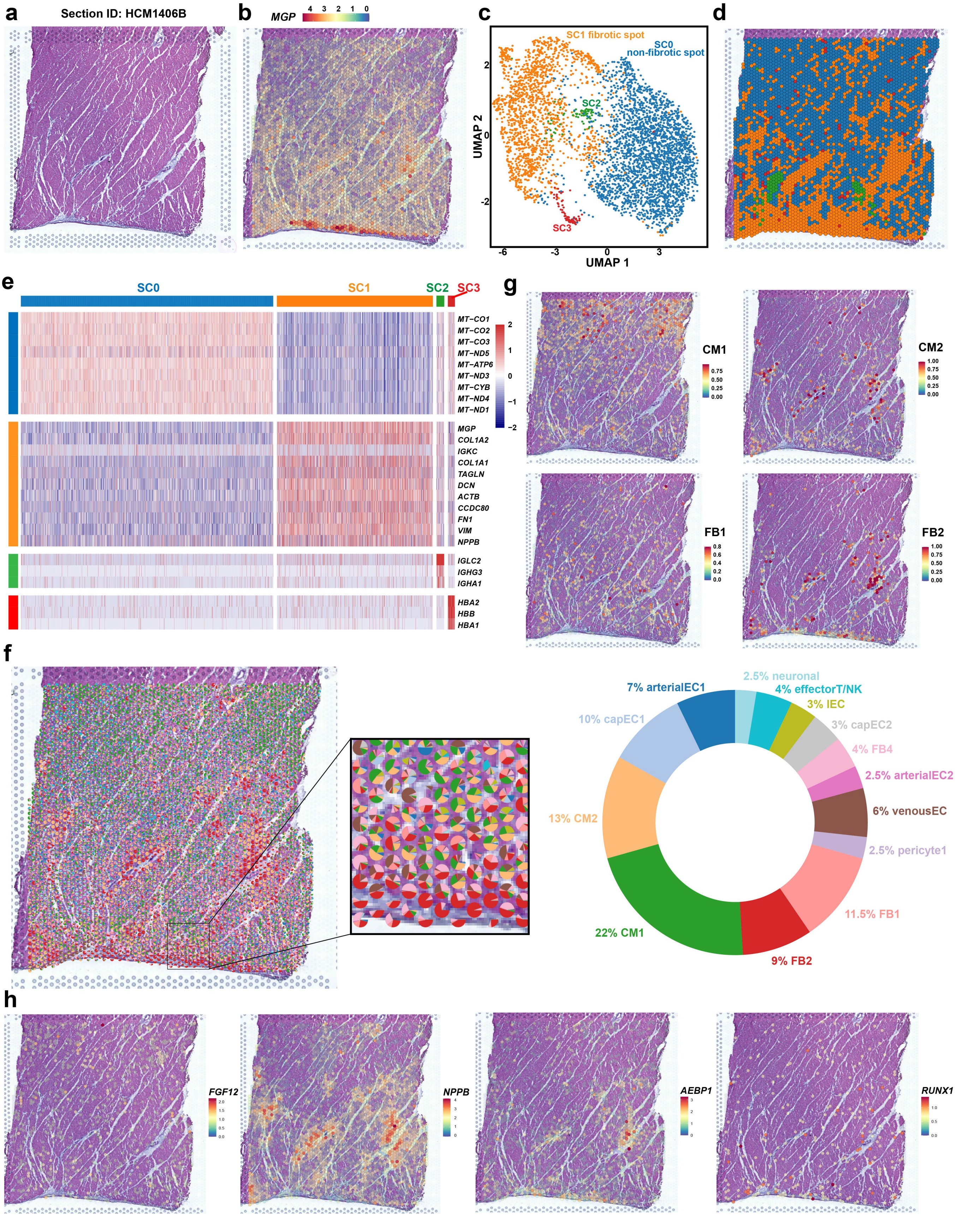

Fig. S21 Spatial transcriptomic analysis of the cardiac tissue section HCM1406B. a H&E staining

image. **b** Expression distribution of the fibrosis marker *MGP*. **c** Clustering of the spots. Spot clusters that correspond to fibrotic and non-fibrotic regions are labeled. **d** Spots color-coded by spot clusters. **e** Heatmap showing the expression signature of each spot cluster. **f** Spot-level cellular subpopulation composition. The pie chart on the right shows the overall composition of the section. Potential noise labels were filtered based on quantile (threshold=0.5). The integration of snRNA-seq and ST data was performed by following the label transfer workflow of Seurat. **g** Probabilistic classification of each spot for the cellular subpopulation CM1, CM2, FB1, and FB2. **h** Expression distribution of representative markers and candidate genes on the section.

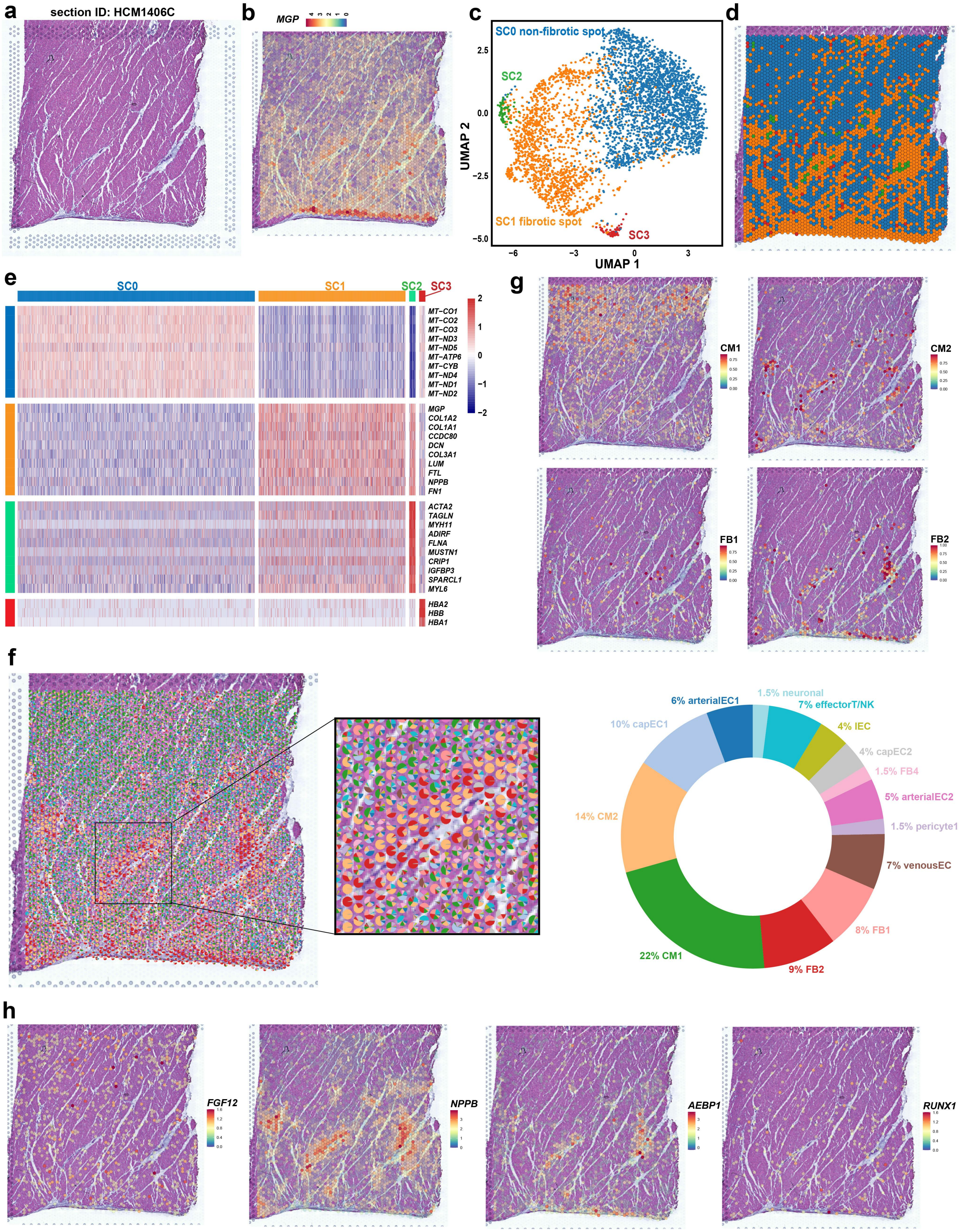

Fig. S22 Spatial transcriptomic analysis of the cardiac tissue section HCM1406C. a H&E staining image. **b** Expression distribution of the fibrosis marker *MGP*. **c** Clustering of the spots. Spot clusters that correspond to fibrotic and non-fibrotic regions are labeled. **d** Spots color-coded by spot clusters. **e** Heatmap showing the expression signature of each spot cluster. **f** Spot-level cellular subpopulation composition. The pie chart on the right shows the overall composition of the section. Potential noise labels were filtered based

on quantile (threshold=0.5). The integration of snRNA-seq and ST data was performed by following the label transfer workflow of Seurat. **g** Probabilistic classification of each spot for the cellular subpopulation CM1, CM2, FB1, and FB2. **h** Expression distribution of representative markers and candidate genes on the section.

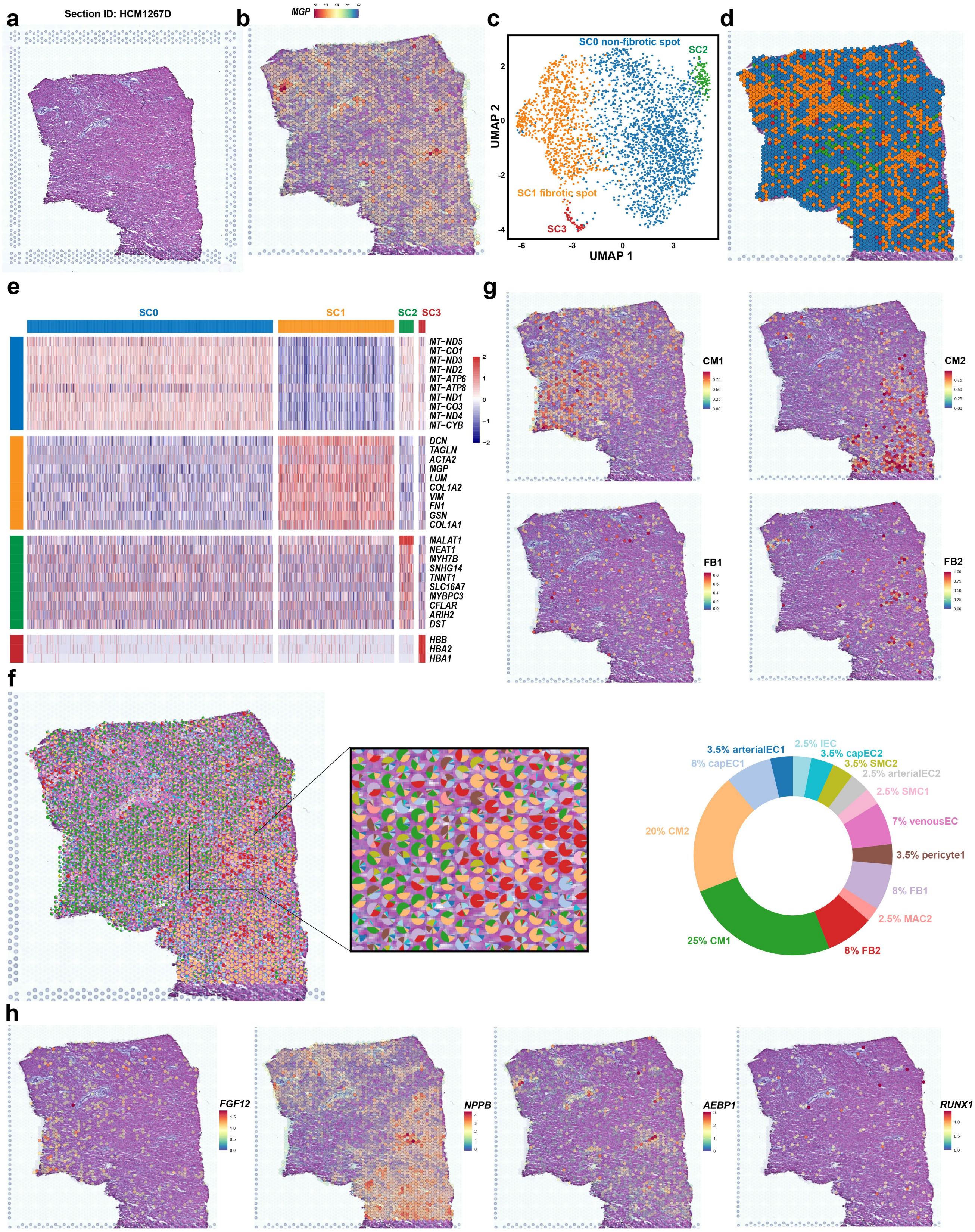

Fig. S23 Spatial transcriptomic analysis of the cardiac tissue section HCM1267D. a H&E staining image. **b** Expression distribution of the fibrosis marker *MGP*. **c** Clustering of the spots. Spot clusters that correspond to fibrotic and non-fibrotic regions are labeled. **d** Spots color-coded by spot clusters. **e** Heatmap

showing the expression signature of each spot cluster. **f** Spot-level cellular subpopulation composition. The pie chart on the right shows the overall composition of the section. Potential noise labels were filtered based on quantile (threshold=0.5). The integration of snRNA-seq and ST data was performed by following the label transfer workflow of Seurat. **g** Probabilistic classification of each spot for the cellular subpopulation CM1, CM2, FB1, and FB2. **h** Expression distribution of representative markers and candidate genes on the section.

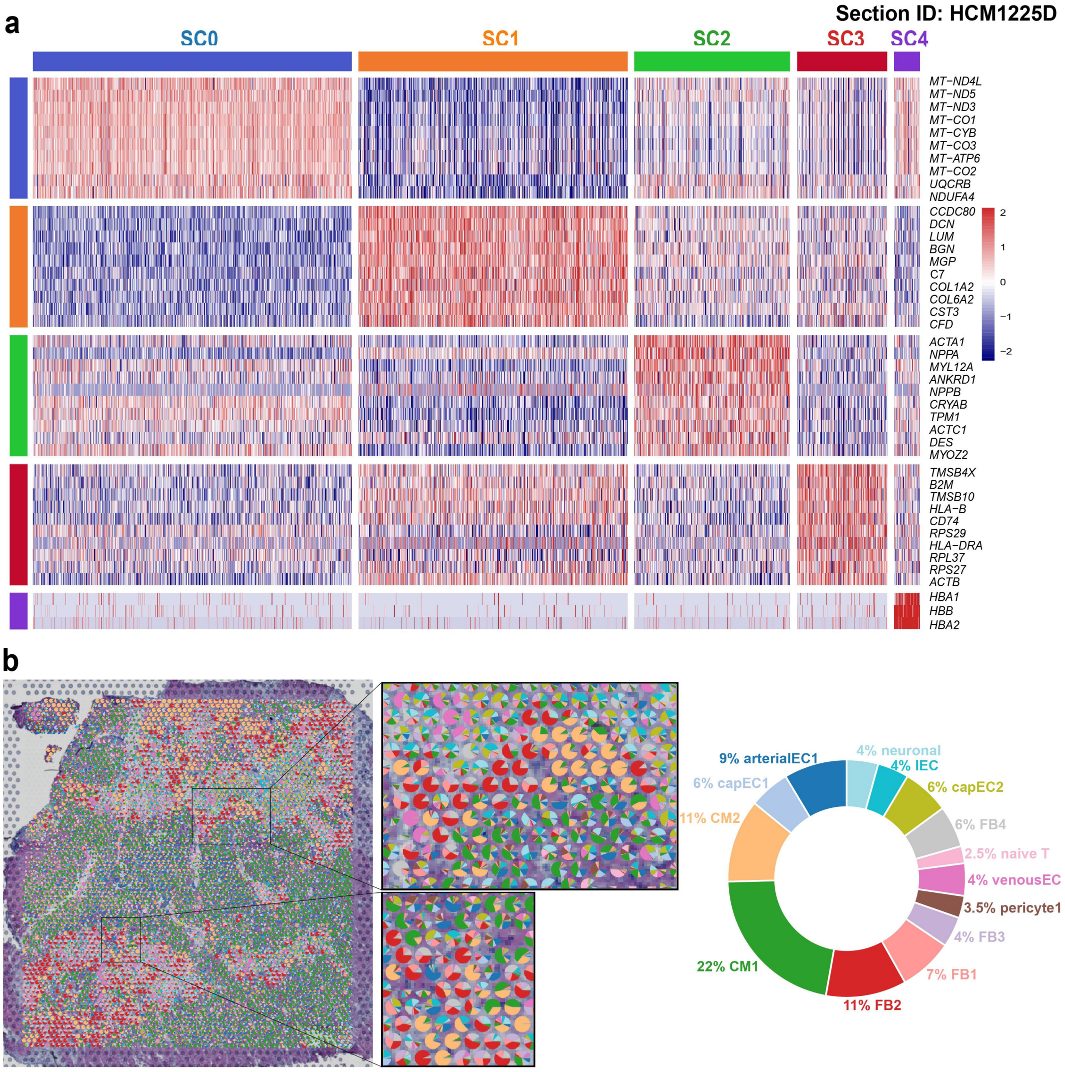

Fig. S24 Expression signature of each spot cluster and spot-level cellular subpopulation composition for the cardiac tissue section HCM1225D. a Expression signature of each spot cluster. **b** Spot-level cellular subpopulation composition for the cardiac tissue section HCM1225D. The pie chart on the right shows the overall composition of the section. Potential noise labels were filtered based on quantile (threshold=0.5). The integration of snRNA-seq and ST data was performed by following the label transfer workflow of Seurat.

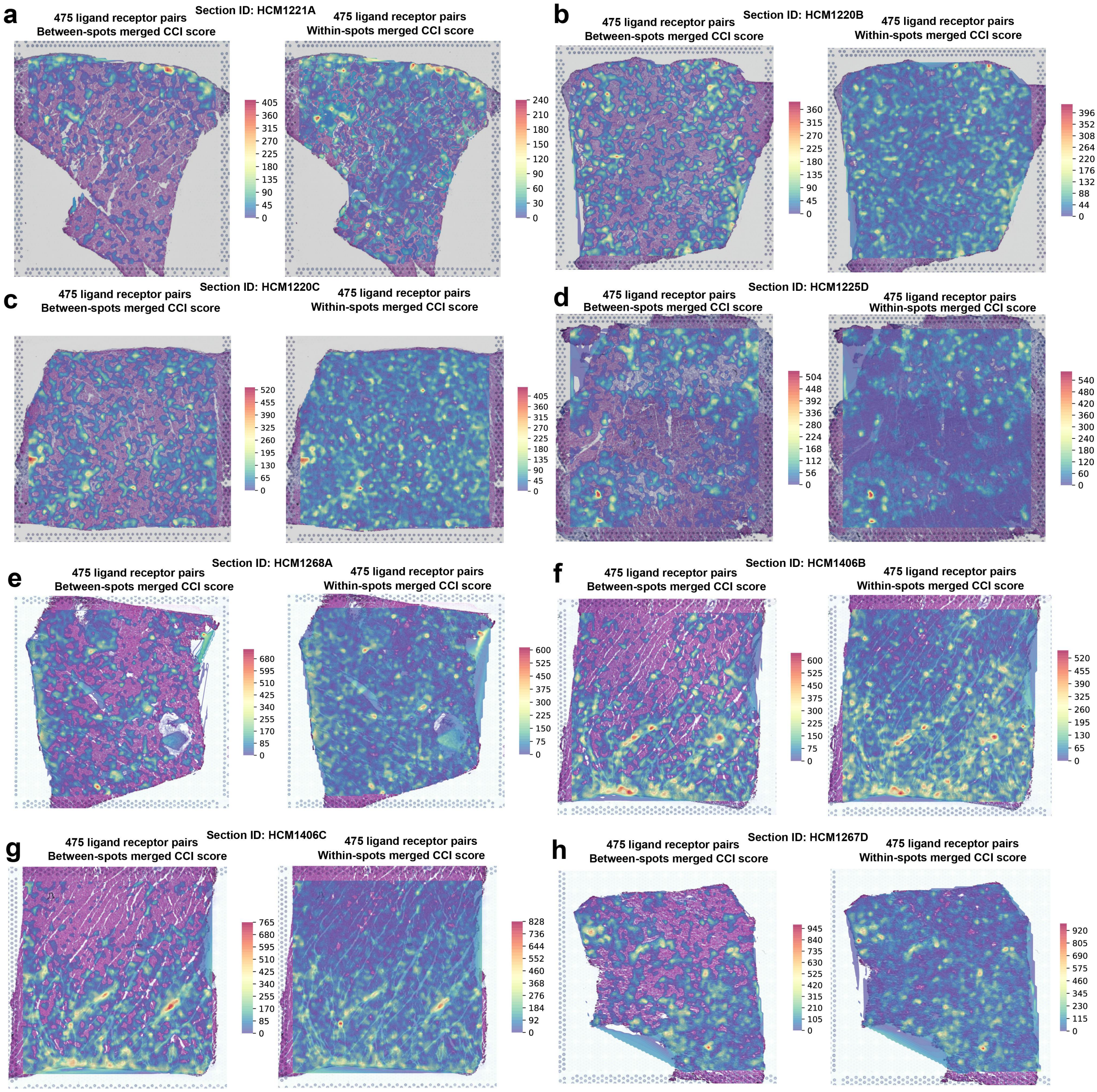

Fig. S25 Spatial CCI analysis by using stLearn revealed that intercellular communication hotspots were mainly localized in fibrotic or peri-fibrotic regions in HCM cardiac tissues. Contour plot showing the spatial distribution of the merged CCI scores in a between-spots mode (left panel) and a within-spots mode (right panel) for the section HCM1221A **(a)**, HCM1220B **(b)**, HCM1220C **(c)**, HCM1225D **(d)**, HCM1268A **(e)**, HCM1406B **(f)**, HCM1406C **(g)**, and HCM1267D **(h)**. All the 475 ligand-receptor pairs that were expressed in the cardiac tissues of HCM predicted by CellChat were considered.

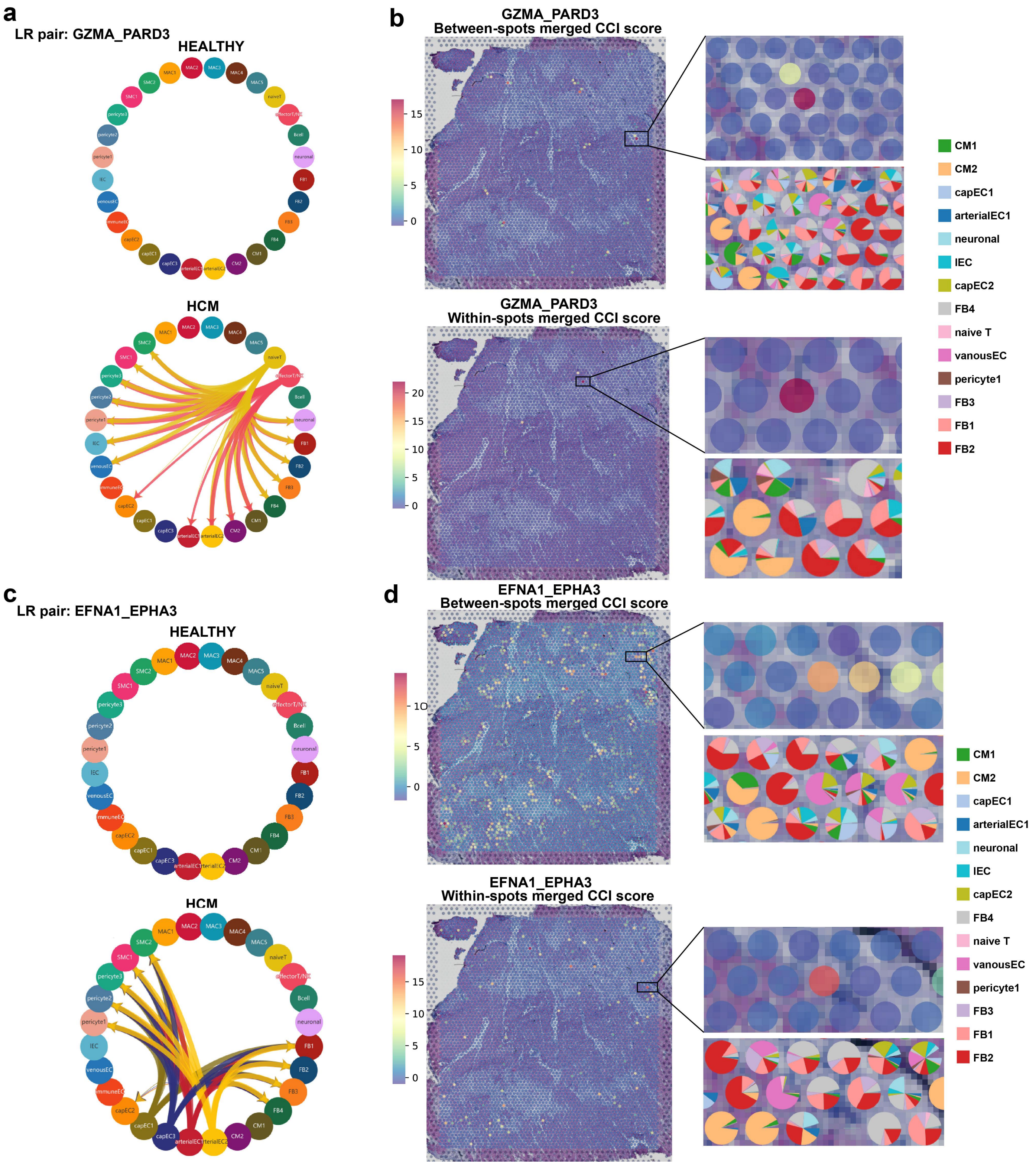

Fig. S26 Representative ligand-receptor pair interactions could be verified by spatial CCI analysis. a Chord plot showing that significant GZMA-PARD3 interaction appeared only in HCM between T cells and other lineages. This result is predicted by CellChat. **b** Contour plot showing the spatial distribution of the merged CCI scores of GZMA-PARD3 in a between-spots mode (upper panel) and a within-spots mode (lower panel). **c** Chord plots showing that significant EFNA1-EPHA3 interaction appeared only in HCM between vascular endothelial cells and other lineages. This result is predicted by CellChat. **d** Contour plot showing the spatial distribution of the merged CCI scores of EFNA1-EPHA3 in a between-spots mode (upper panel) and a within-spots mode (lower panel). In **b** and **d**, the magnified region shows the spots with high CCI scores of the ligand-receptor pair. Pie charts show the spot-level cellular composition which supports the spatial interaction of related cell lineages.

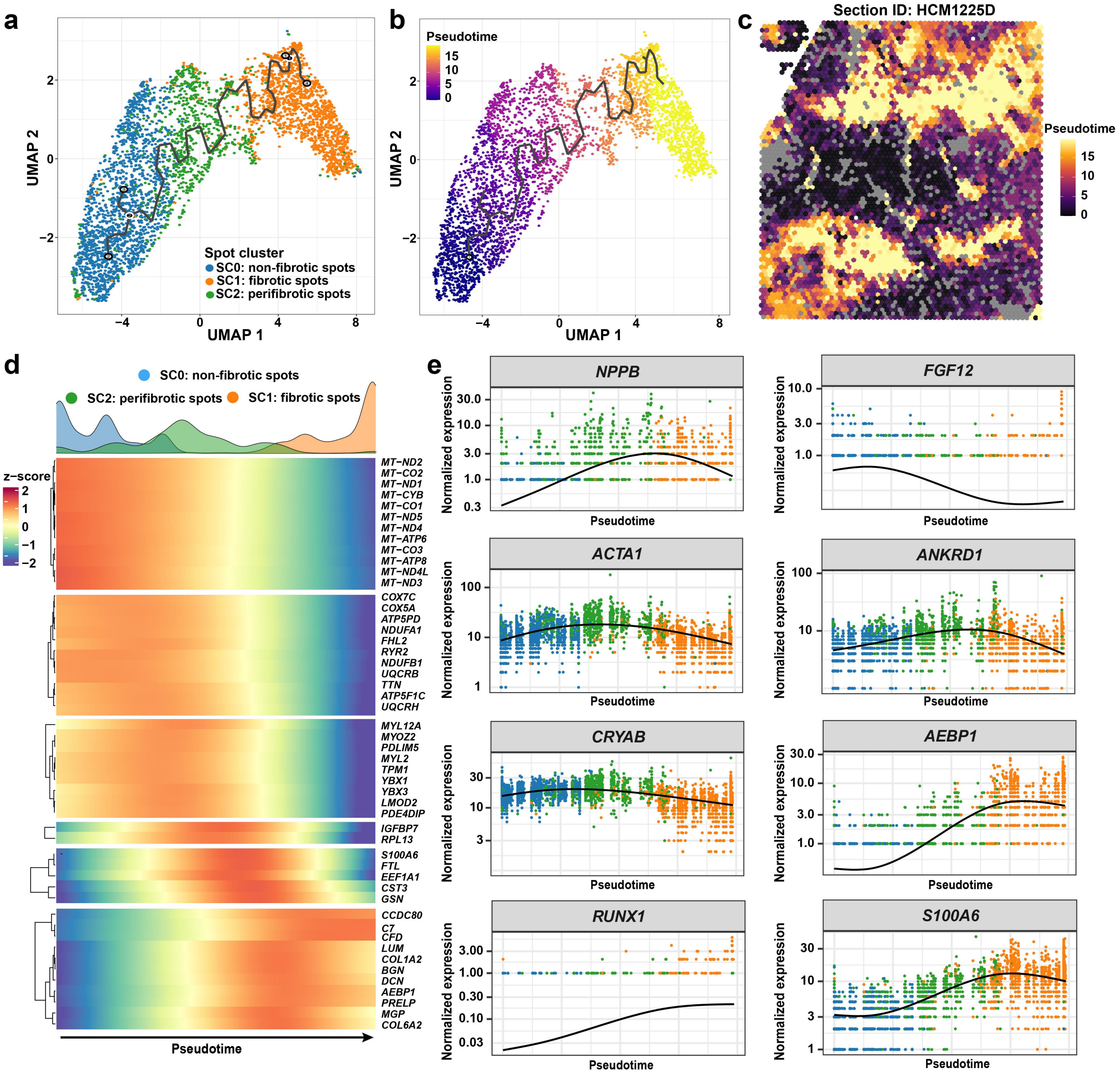

Fig. S27 Spatial pseudotime analysis identified the transcriptomic dynamics during the change from non-fibrotic to fibrotic states of cardiac tissues in HCM. a UMAP plot showing spot clusters and the inferred trajectory from non-fibrotic to fibrotic spots. Spatial trajectory (indicated by a black curve) was inferred by pseudotime ordering of the spatial spots using Monocle3. Only the three major clusters were considered: SC0: non-fibrotic spots. SC1: fibrotic spots. SC2: peri-fibrotic spots. **b** UMAP plot showing the pseudotime of each spot. **c** Visualization of the pseudotime in a spatial context. Spots that were not considered (e.g., blood spots) in this analysis are colored in grey. **d** Heatmap showing the transcriptomic dynamics during the change from non-fibrotic to fibrotic states of cardiac tissue in HCM. Only genes that met the following threshold were considered as significantly changed genes: q value < 0.05 and morans I > 0. The top 50 significantly changed genes are shown. K-means clustering was performed on the genes. **e** Smoothed expression of representative markers and candidate genes along the pseudotime. Only the section HCM1225D was considered for spatial pseudotime analysis because it represents a typical section with a clear separation of fibrotic and non-fibrotic regions.

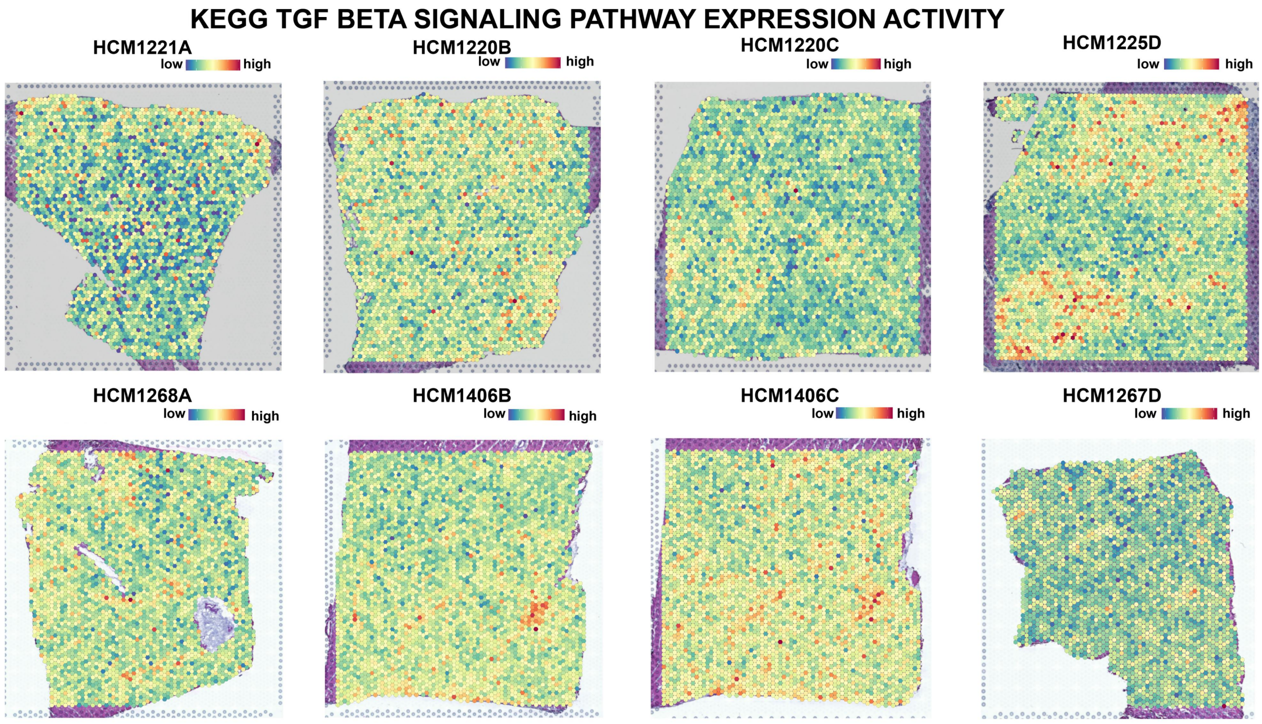

Fig. S28 The spatial distribution of expression activity of the TGFβ signaling pathway.

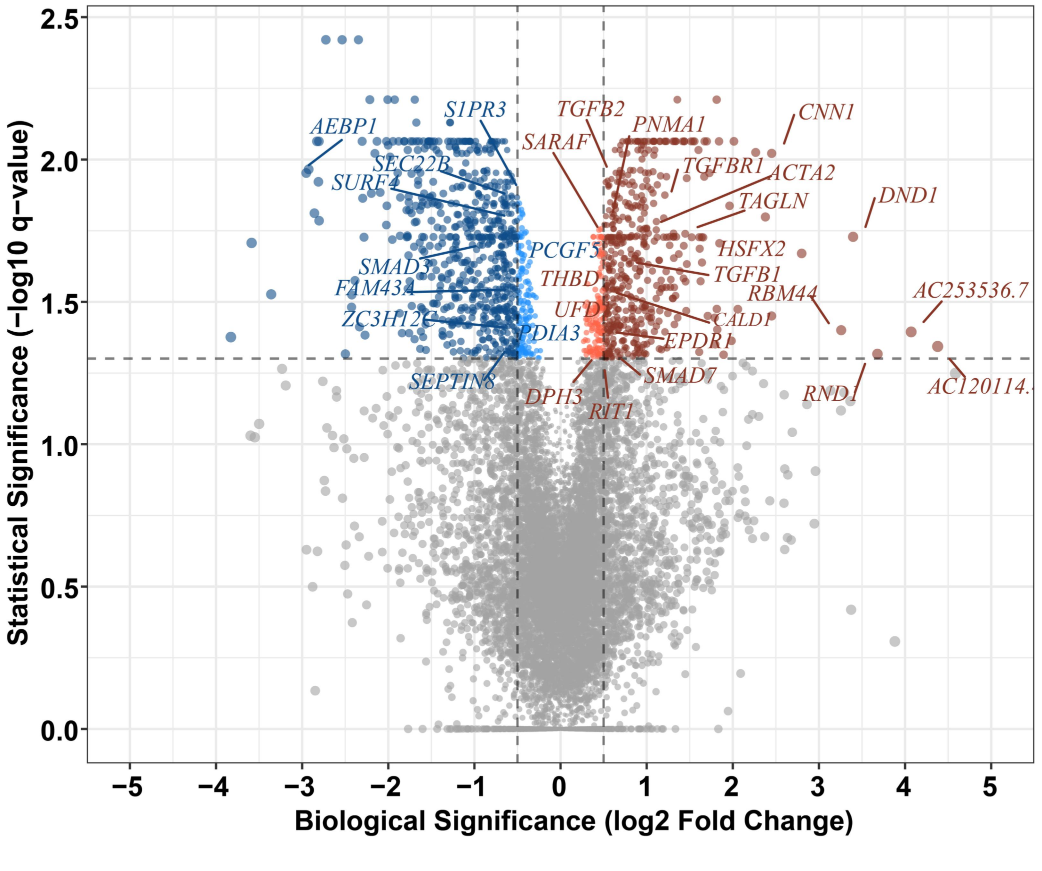

Fig. S29. Volcano plot showing the differentially expressed genes obtained by bulk RNA-seq in *AEBP1*-siRNA versus scrambled siRNA. The statistical significance threshold was set to a q-value < 0.05, and the biological significance threshold was set to an absolute log2 fold-change > 0.5

Supplemental Table Titles

Table S1. Demographic and clinical information of subjects recruited in this study.

Table S2. Quality metrics of the snRNA-seq dataset.

Table S3. Quality metrics of the spatial transcriptome dataset.

Table S4. Gene signature of each subpopulation in each cell type (multiple sheets).

Table S5. Differentially expressed genes in each cell type between HCM and HEALTHY (multiple sheets).

Table S6. Functional enrichment analysis of the upregulated genes in each cell type between HCM and HEALTHY (multiple sheets). Neuronal cells, lymphatic endothelial cells, and T/NK cells are not considered in this analysis due to the limited number of cells.

Table S7. Differentially regulated pathways in each cell type between HCM and HEALTHY detected by gene set enrichment analysis (multiple sheets). Neuronal cells, lymphatic endothelial cells, and T/NK cells are not considered in this analysis due to the limited number of cells.

Table S8. Differential regulatory network analysis in each cell type between HCM and HEALTHY (multiple sheets).

Table S9. Genes with differential expression patterns along the pseudotime between HCM and HEALTHY in cardiomyocytes (sheet1) and fibroblasts (sheet2) detected by tradeSeq.

Table S10. Cell-type specific differentially expressed genes through pseudobulk analysis.

Table S11. Regulons in cardiomyocytes and fibroblasts predicted by SCENIC and their mean activity scores in HCM and HEALTHY (multiple sheets).

Table S12. Ligand-receptor interactions in cardiac tissues of HCM (sheet1) and HEALTHY (sheet2) inferred by CellChat.

Table S13. Ligand-receptor pairs expressed in the cardiac tissues of HCM that were predicted by CellChat and their accumulated communication activities in the cardiac tissues of HCM and HEALTHY.

Table S14 Transcriptomic dynamics during the change from non-fibrotic to fibrotic states of cardiac tissues in HCM identified by spatial pseudotime analysis.

Table S15. Differentially expressed genes of spatial transcriptomic spots in fibrotic versus non-fibrotic regions of cardiac tissue sections from HCM patients (multiple sheets).

Table S16. Differentially regulated pathways of spatial transcriptomic spots in fibrotic versus non-fibrotic regions of cardiac tissue sections from HCM patients.

Table S17. Differential expression analysis of human cardiac fibroblasts in *AEBP1* knockdown versus negative control through bulk RNA-seq.

Table S18. Information of the antibodies used in western blot assays and immunofluorescence staining.
